# Cyclic changes in gene expression induced by Peg-interferon alfa-2b plus ribavirin in peripheral blood monocytes (PBMC) of hepatitis C patients during the first 10 weeks of treatment

**DOI:** 10.1186/1479-5876-6-66

**Published:** 2008-11-05

**Authors:** Milton W Taylor, Takuma Tsukahara, Jeanette N McClintick, Howard J Edenberg, Paul Kwo

**Affiliations:** 1 Department of Biology, Indiana University, Bloomington, IN. 47401, USA; 2 Department of Biochemistry and Molecular Biology and Center for Medical Genomics, Indiana University School of Medicine, Indianapolis, IN 46202, USA; 3 Department of Medicine, Hepatology Unit, Indiana University School of Medicine, Indianapolis, IN 46202, USA

## Abstract

**Background and Aims:**

This study determined the kinetics of gene expression during the first 10 weeks of therapy with Pegylated-interferon-alfa2b (PegIntron™) and ribavirin (administered by weight) in HCV patients and compared it with the recently completed Virahep C study [1,2] in which Peginterferon-alfa2a (Pegasys™) and ribavirin were administered.

**Methods:**

RNA was isolated from peripheral blood monocytes (PBMC) from twenty treatment-naïve patients just before treatment (day 1) and at days 3, 6, 10, 13, 27, 42 and 70 days after treatment. Gene expression at each time was measured using Affymetrix microarrays and compared to that of day 1.

**Results:**

The expression of many genes differed significantly (p ≤ 0.001 and changed at least 1.5-fold) at days 3 (290 probes) and 10 (255 probes), but the number dropped at days 6 (165) and 13 (142). Most genes continued to be up regulated throughout the trial period. A second group of genes, including *CXCL10*, *CMKLR1 *(chemokine receptor 1), *TRAIL*, IL1Rα and genes associated with complement and lipid metabolism, was transiently induced early in treatment. *CDKN1C *(cyclin kinase inhibitor 1) was induced early but repressed at later times. Genes induced at later times were mostly related to blood chemistry and oxygen transport. By week 10, 11 of the patients demonstrated a positive response to therapy, and the final sustained viral response (SVR) was 35%. The levels of gene induction or decrease was very similar to that previously reported with Pegasys/ribavirin treatment.

**Conclusion:**

The response to Pegintron/ribavirin was similar to that reported for Pegasys/ribavirin despite some differences in the amount administered. We did not detect major differences at the genomic level between patients responding to treatment or non-responders, perhaps because of limited power. Gene induction occurred in a cyclic fashion, peaking right after administration of interferon and declining between administrations of the drug. Our data suggest that more than once a week dosing might be desirable early during treatment to maintain high levels of response as measured by gene expression.

## Background

Hepatitis C virus (HCV) infection is a significant global public health problem, affecting approximately 200 million individuals in the world and over 4 million in the United States alone, where it is the most prevalent blood-borne infection [3]. It is currently the leading indication for a liver transplant and is responsible for 8,000–10,000 deaths annually. Interferon (IFN) has formed the backbone of therapy against HCV, first as monotherapy, then in combination with the nucleoside analogue ribavirin [4]. Current standard of care for chronic HCV infection consists of a regimen of pegylated interferon-α in combination with ribavirin. The addition of the polyethylene glycol (PEG) moiety (pegylation) increases the half-life of the IFN molecule and has facilitated once per week dosing instead of the two or three doses per week previously required with non-pegylated forms of IFN [5,6]. The combination of pegylated IFN-α and ribavirin successfully eradicates the virus from 50–60% of those treated [7,8].

Two different pegylated molecules of IFN have been approved for clinical use in the U.S. The size and position of the PEG moiety differs between pegylated-interferon-α-2a (Pegasys™) and Pegylated-interferon-α-2b (PegIntron™) [5,9,10]. Although pegylation improves the pharmacokinetic properties of the core IFN protein [11], it decreases the *in vitro *biological activity [12,13]. PegIntron™ has higher *in vitro *anti-viral activity than Pegasys™ [11,14,15] (Taylor, unpublished data).

Type I IFNs do not directly inactivate the virus, but exert their effects through binding to specific receptors on the cell surface, IFNAR1 and IFNAR2 [16]. This results in a cascade of gene activation through the Jak-STAT pathway [17-19] and perhaps other transcription pathways [20,21]. Large number of genes are induced or down regulated by non-pegylated IFNα *in vitro *[22-25]. Previous work [15] has shown very similar *in vitro *profiles of gene induction in monocytes (PBMC) treated with either pegylated or the non-pegylated version of IFNα. Virtually all of the changes in gene expression were due to the IFN, rather than the ribavirin [23]. We have recently reported that the expression of many hundreds of genes are significantly modified, both up and down, *in vivo *following treatment of hepatitis C patients with pegylated-IFNα-2a (Pegasys™) and ribavirin [2]. Using a mathematical model we identified a core set of genes that appear to be related to the anti-viral effects. These include *OAS2*, *MX1*, *MX2*, *RIG1*, genes associated with ubiquitination, and many other genes previously shown to be associated with interferon treatment [26].

In this report we analyze the response of patients to combination treatment with pegylated-interferon-α_2b _(PegIntron™) and ribavirin during the first 10 weeks of treatment. Unlike the previous report from the Virahep C study [2], which used a constant dose of 180 ug of pegylated-interferon-α-2a, in the present study the PegIntron™ was administered in an amount related to the body weight of the patients. Blood samples were collected before treatment initiation (day 1) and at days 3, 6, 10, 13, 27, 42 and 70 after treatment. Interferon injections were weekly at day 1, 7, 14 etc. The selection of days was based on times just before interferon injection (days 6,13, 27, 42 and 70) in order to analyze whether there was a trough in gene expression at the end of the weekly period. Affymetrix microarrays were used to detect genes up- or down- regulated during treatment. Viral assays for the presence of HCV in serum were performed at the same time points. In this study we report that changes in gene expression levels are high 3 days after IFN injection and return toward baseline before the next injection; the return toward baseline is accompanied in many cases by a slight increase in virus titer. This pattern continues for the first few weeks. Genes induced by the treatment fall into three classes, genes that are up regulated throughout the treatment, immediate expressed genes with only transient expression, and late genes in which expression is elevated only after day 27. Fifty percent of the patients showed an antiviral response during the first 10 weeks, but the final SVR was 35%.

## Materials and methods

### Subjects

Twenty (16 M, 4 F) genotype 1 hepatitis C patients who gave informed consent were entered into this trial. All subjects were previously untreated, and had no other cause of chronic liver disease, ALT levels above the upper limit of normal, compensated liver disease with minimal hematological parameters including hemoglobin values of 12 gm/dL for females and 13 gm/dL for males, WBC > 3,000/mm^3^, neutrophil count > 1,500/mm^3^, platelets > 70,000/mm^3 ^and no evidence of decompensation in those with cirrhosis. All patients had liver biopsies within 3 years of enrolling, with fibrosis graded by the Metavir scoring system. Patients were excluded if they had decompensated cirrhosis, serum α-fetoprotein concentration above 50 ng/L, HIV infection, previous organ transplantation, other causes of liver disease, pre-existing psychiatric disease, seizure disorders, cardiovascular disease, haemoglobinopathies, haemophilia, poorly controlled diabetes, or autoimmune-type disease, or if they were unable to use contraception. Table 1 presents the demographics of the population used in this study. This study was approved by the institutional review board.

**Table 1:** Pretreatment characteristics of the patients

Patient ID	Age	Weight (kg)	Genotype	Gender	Fibrosis Score Metavir	ALT (IU/L)	Day 1 HCV RNA level (IU/Ml)
1	49	76	1a	1	4	74	1.7E+06
2	52	92	1a	0	0	20	7.0E+05
3	52	92	1a	1	4	57	3.4E+06
4	38	90	1a	1	0	52	1.1E+07
5	56	80	1a	1	2	68	3.5E+06
6	47	106	1a	1	3	146	1.1E+07
7	44	65	1b	1	2	127	1.1E+07
8	42	112	1a	1	1	67	7.2E+06
9	52	78	1a	1	4	71	2.3E+06
10	57	76	1b	0	2	68	7.1E+06
11	59	70	1b	0	3	62	2.4E+06
12	56	109	1a	1	4	74	5.8E+06
13	49	116	1a	1	2	44	5.8E+06
14	53	90	1b	1	4	98	3.7E+05
15	51	61	1a	0	3	73	1.8E+06
16	50	78	1a	1	3	99	1.6E+06
17	60	106	1a	1	2	37	4.1E+05
18	45	114	1a	1	4	161	9.0E+05
19	47	74	1b	1	3	117	4.0E+05
20	61	83	1b	1	2	181	8.1E+05

Patients received PegIntron™ at 1.5 μg/kg (based upon weight at initial visit) administered subcutaneously once a week for 10 weeks (days 1, 7, 14, 21, 28 ...), plus ribavirin (13 ± 2 mg/kg/day). Patients had blood drawn for analysis on day 1 prior to first injection of interferon (base line) and at days 3, 6, 10, 13, 27, 42 and 70.

### HCV-RNA Serum Determinations

Serum samples were collected before treatment initiation (day 1) and at days 3, 6, 10, 13, 28, 42, 70 and weeks 12, 24, 48 and 72, for viral assays. HCV-RNA was determined by qRT-PCR (TaqMan^®^, Schering-Plough Research Institute, Union, NJ) with a lower limit of detection of 29 IU/ml.

### Peripheral Blood Mononuclear Cell (PBMC) Preparation

Blood was collected in sodium heparin-CPT tubes, diluted with an equal volume (8 ml) of phosphate buffered saline (PBS), carefully layered over a 10 ml Ficoll-Hypaque gradient (Amersham/Pharmacia, Piscataway, NJ) and centrifuged at 800 rpm for 20 minutes at room temperature. The buffy coat layer was transferred to a 15 ml RNAse-free tube, diluted with PBS, and centrifuged at 100 × g for 15 minutes at room temperature. The supernatants were discarded and the PBMC were retained.

### RNA Extraction

The PBMC were lysed in 1 ml of TRI reagent (Molecular Research Center Inc, Cincinnati, OH). The lysate was mixed with 1-Bromo-3-chloropropane (BCP)-phase separation agent for 1 minute, and incubated at room temperature for 15 minutes. After centrifugation for 15 minutes at 12,000 rpm and 4°C, RNA was precipitated from the supernatant overnight at -20°C with an equal volume of isopropanol and 1/10 volume of 7.5 M ammonium acetate. The precipitate was washed twice with 75% ethanol, and then with 95% ethanol. RNA was briefly air-dried and then resuspended and further purified using RNeasy columns (Qiagen; Valencia, CA). The amount and quality of RNA were determined by spectrophotometry and by electrophoresis through 1% agarose with ethidium bromide. RNA was further analyzed by the Agilent Bioanalyzer; samples that did not show two clear bands of ribosomal RNA were discarded.

### RNA Labeling, Hybridization and Scanning

Preparation of cDNA, cRNA, and labeling were carried out according to the protocols recommended by Affymetrix in the GeneChip^® ^Expression Analysis Technical Manual (Affymetrix, Santa Clara, CA), as previously described [2]. Hybridization was to Affymetrix GeneChip^® ^Human Genome U133A microarrays, which measure approximately 22,000 genes. The microarrays were scanned using a dedicated Affymetrix Model 3000 7G scanner controlled by GCOS software.

### Statistical Analysis

The average intensity on each array was normalized by global scaling to a target intensity of 1000. Data were extracted using the Affymetrix Microarray Suite 5 (MAS5) algorithm. To avoid analyzing genes that were not reliably detected, the MAS5 data were filtered to eliminate any gene that was not called present in at least 50% of the samples in at least one group [27]. If a probeset was not reliably detected on day 1 but was later, it is noted as "turned on" and if it was detected on day 1 but not later it is noted as "turned off;" the exact fold change for such genes are not reliable because the signal for a gene that is not detected is largely background. Fold changes for each gene were calculated using the ratio of the MAS5 signals of the post treatment time to the baseline (pre-treatment). If the signal for the post-treatment time point was greater than the baseline the fold change was calculated as +average(post-treatment)/average(baseline), otherwise the fold change was calculated as -average(baseline)/average(post-treatment). Genes were considered significant if the paired t-test p-value of log(signal) ≤ 0.001 and the fold change was at least 1.5.

Gene expression as a function of time was analyzed using Edge [28]; values are calculated on the log transform, but are plotted as percent of maximum signal values with gnuplot  to show wider range of values. The 90 genes most significant across all time points (by 1-way ANOVA) were clustered using Pearsons dissimilarity and average linkage, using Partek Genomics Suite (Partek Inc. St. Louis, MO); arrays were ordered by day to show the pattern of expression across time.

## Results

Of the 20 patients enrolled, 19 were European American and one was African American. Sixteen were male. All were genotype 1, 14 with genotype 1A and 6 with genotype 1B. The baseline features of the 20 patients in this study are shown in Table 1. In this cohort, 11/20 had advanced hepatic fibrosis (Metavir stage 3–4), with 17/20 having high viral load (> 600,000 IU/mL). The overall sustained viral response rate (SVR) at the end of treatment (72 weeks) was 35%; i.e. 7/20 individuals had undetectable virus at 72 weeks. Table 2 presents virus titers with time. By week 12 there were 11/20 patients who cleared virus, however one withdrew from treatment because of severe side effects, and 2 relapsed by the end of treatment.

**Table 2:** Viral titer with time.

Patient ID	Day 0 HCV RNA level (IU/Ml)	Day 3	Day 7	Day 10	Day 13	Day 27	Week 6	Week 10	Viral Response*	Week 12	Week 24	Week 48	Week 72	Final Response
1	1.7E+06	3.5E+05	3.7E+05	1.0E+05	2.3E+05	9.6E+04	8.3E+04	5.3E+03	NR	2.0E+03	0	0	NR	NR
2	7.0E+05	1.3E+05	9.2E+04	1.5E+03	3.8E+03	0	0	0	R	0	0	0	0	R
3	3.4E+06	1.5E+06	2.8E+06	1.1E+06	1.9E+06	1.6E+06	1.9E+06	6.3E+05	NR	3.8E+05			NR	NR
4	1.1E+07	3.5E+05	6.1E+05	3.6E+04	1.7E+05	9.0E+03	5.7E+02	0	R	0	0	0	0	R
5	3.5E+06	2.9E+06	1.6E+06	4.9E+05	1.4E+06	6.9E+05	4.7E+05	2.0E+05	NR	1.7E+05			NR	NR
6	1.1E+07	4.0E+06	5.6E+06	3.9E+06	6.3E+06	2.3E+06	1.0E+06	2.2E+05	NR	7.5E+05			NR	NR
7	5.8E+06	1.3E+05	9.9E+04	1.4E+04	7.3E+04	1.3E+03	1.5E+02	0	R	0	0	0	0	R
8	1.1E+07	1.9E+05	5.8E+05	2.2E+04	no sample	2.6E+03	8.7E+01	0	R	0	0	0	0	R
9	7.2E+06	8.8E+03	2.2E+05	1.9E+03	4.4E+03	0	0	0	R	0	0	0	0	R
10	2.3E+06	1.7E+06	5.3E+06	6.1E+05	1.0E+06	5.5E+05	3.6E+05	8.0E+05	NR	3.1E+05			NR	NR
11	7.1E+06	1.9E+06	2.7E+06	2.0E+06	5.8E+06	1.9E+06	1.9E+06	1.4E+06	NR	6.7E+05			NR	NR
12	2.4E+06	1.7E+07	3.3E+06	1.3E+06	2.0E+06	7.3E+05	5.4E+05	1.0E+05	NR	6.6E+04			NR	NR
13	5.8E+06	9.9E+05	9.2E+05	1.1E+05	1.9E+05	1.4E+05	7.4E+03	1.5E+02	R	0	0	0	0	R
14	3.7E+05	1.0E+05	2.3E+05	7.0E+04	1.2E+05	7.0E+03	3.9E+02	1.6E+02	R	0	0	0	1.2E+06	NR
15	1.8E+06	3.8E+05	1.0E+06	4.2E+05	8.0E+05	1.9E+05	2.3E+04	2.0E+03	R	3.6E+03			NR	NR
16	1.6E+06	2.1E+06	3.9E+06	8.0E+05	5.8E+06	5.4E+06	2.1E+06	1.3E+06	NR	7.7E+04			NR	NR
17	4.1E+05	1.2E+05	1.2E+06	1.3E+05	6.1E+05	3.1E+04	7.5E+03	4.5E+01	R	0	0	0	0	R
18	9.0E+05	1.5E+06	6.2E+06	4.3E+05	1.4E+06	3.2E+05	3.3E+05	2.5E+05	NR	4.8E+04			NR	NR
19	4.0E+05	8.7E+04	6.9E+05	3.1E+04	3.7E+04	1.5E+03	1.0E+03	0	R	0	0	0	6.3E+06	NR
20	8.1E+05	3.3E+03	1.9E+03	3.8E+01	2.7E+02	0	0	0	R	0	W	W	W	withdrew

*R = responder, NR = non-responder.

W = withdrew

### Changes in Global Gene Expression

**Figure 1:**
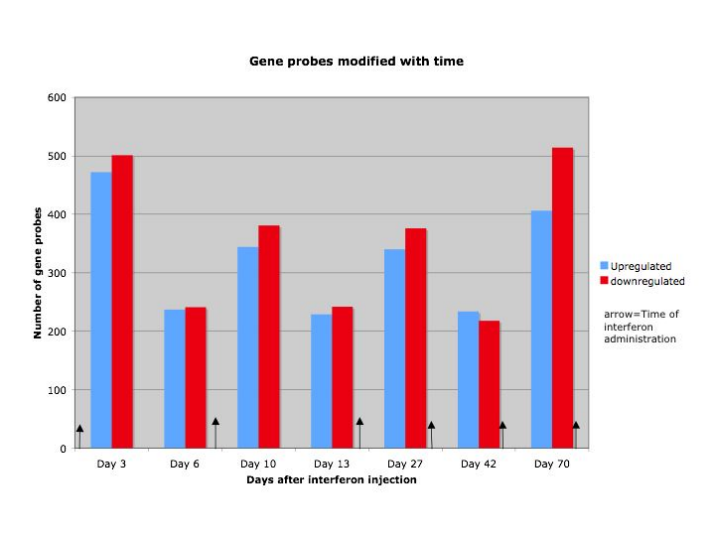
The number of genes significantly upregulated or down-regulated (p ≤ 0.001) at each time point.

Gene expression in PBMC changed dramatically and rapidly during PEG-interferon-α2b (PegIntron™)/ribavirin therapy, with major changes being evident at all days after the initial administration of the drugs (Table 3, Figure 1). There was no significant difference in response between patients with genotype 1A and 1B, nor between responders and non-responders, so all patients were analyzed together. 973 genes were significantly (p ≤ 0.001; False Discovery Rate [29] 1.2%) induced or down regulated on day 3; the number induced was approximately the same as the number down-regulated, as was seen in our earlier study [2]. The number of differentially expressed genes varied with time (Table 3, Figure 1); it was high on days 3 and 10 (mid-way between injections) and much lower on days 6, 13 and 42 (just before subsequent injections) (Table 3, Figure 1). The number of genes with altered expression was high again, particularly for down regulated genes, at day 70. Half of the up-regulated genes but only 16% of the down-regulated genes showed at least 1.5-fold change (Table 3). For our subsequent analyses we focused on the genes with more robust changes (p = 0.001 and absolute fold-change ≥ 1.5).

**Table 3:** Number of probe sets that significantly differed in expression (p ≤ 0.001).

Fold Change	Expected*	Day 3	Day 6	Day 10	Day 13	Day 27	Day 42	Day 70
NA**	11	973	478	725	471	716	452	920
≥ 1.5	-†	290	165	255	142	194	157	289
%≥ 1.5		30%	35%	35%	30%	27%	35%	31%
								
Up regulated								
Fold Change	Expected*	Day 3	Day 6	Day 10	Day 13	Day 27	Day 42	Day 70
NA**	-†	472	237	344	229	340	234	406
≥ 1.5	-†	235	96	294	102	129	103	206
%≥ 1.5		50%	41%	85%	45%	38%	44%	51%
								
Down regulated								
Fold Change	Expected*	Day 3	Day 6	Day 10	Day 13	Day 27	Day 42	Day 70
NA**	-†	501	241	381	242	376	218	514
≤ -1.5	-†	55	32	61	40	65	54	83
%≥ 1.5		11%	13%	16%	17%	17%	25%	16%

* Expected for normal distribution

** Not applicable; No fold change cutoff applied

† Not applicable

There were 69 genes that showed at least 1.5-fold differences in expression at either 6 or all 7 time points: 59 up-regulated and 10 down-regulated (Table 4). Many of these up regulated genes have previously been shown to be regulated by interferon [2,25,26]. A full list of all genes induced or down regulated at p ≤ 0.001 at any one day compared to day 1 is presented in Supplementary Table 1.

**Table 4:** Genes differentially expressed (1.5-fold) at ≥ 6 time points

Symbol	Day 3	Day 6	Day 10	Day 13	Day 27	Day 42	Day 70	Description
**Up regulated**								
Gene name								Other name/function
AGRN	2.1	1.7	2.1	1.9	1.6	1.7	2.1	agrin
APOBEC3A	2.9	2.0	2.7	*1.9*	1.9	1.8	2.0	apolipoprotein B mRNA editing enzyme, catalytic polypeptide-like 3A
CHMP5	2.8	1.9	2.6	2.0	1.9	1.8	1.9	chromatin modifying protein 5
DDX58	2.8	1.8	2.6	1.7	1.8	1.6	1.7	RNA helicase RIG-I
EIF2AK2	2.6	1.9	2.6	2.2	2.0	2.0	2.4	Eukaryotic translation initiation factor 2-alpha kinase 2
FLJ11286	1.8	1.5	1.8	1.6	1.5	1.6	1.6	C19orf66
FLJ20035	3.8	3.0	3.6	2.7	2.5	2.6	2.7	DDX60
H1F0	3.1	1.6	2.1	1.8	1.9	2.1	2.5	H1 histone family, member 0
HERC5	4.5	2.1	4.1	2.5	2.6	2.5	2.7	Ubiquitin ligase/mediates ISGylation of protein targets
HERC6	5.9	4.1	5.7	4.1	4.1	4.2	4.2	Ubiquitin ligase
HIST1H1C	2.6	1.6	2.4	1.7	2.6	2.4	3.2	histone cluster 1, H1c
HIST1H2AE	2.4	2.0	3.3	2.1	2.5	2.6	3.8	histone cluster 1, H2ae
HIST1H2BC	2.5	2.0	2.7	1.9	2.0	*2.2*	2.8	histone cluster 1, H2bg///histone cluster 1, H2bc
HIST1H2BD	2.1	1.7	2.3	1.6	1.7	1.6	2.2	histone cluster 1, H2bd
HIST1H2BF	2.4	1.9	2.5	1.8	1.9	*1.5*	2.2	histone cluster 1, H2bf
HIST1H2BG	4.3	2.8	3.6	3.5	3.3	*2.4*	3.4	histone cluster 1, H2bg
HIST1H2BI	2.4	1.9	2.5	1.9	2.0	*1.5*	2.2	histone cluster 1, H2bi
HIST2H2AA3	3.1	1.8	2.8	1.8	2.1	2.1	2.8	histone cluster 2, H2aa3///histone cluster 2, H2aa4
IFI27	73.3	70.3	98.0	93.5	94.8	97.4	107.7	interferon, alpha-inducible protein 27 (ISG12, P27)
IFI35	3.2	2.0	2.9	2.0	1.8	1.9	1.9	interferon-induced protein 35
IFI44	7.1	4.8	6.5	4.8	4.5	5.2	5.0	Interferon-induced protein 44, p44
IFI44L	10.1	8.2	9.7	7.6	7.5	8.0	8.4	interferon-induced protein 44-like
IFIH1	3.3	2.0	3.3	2.4	2.1	2.3	2.2	interferon induced with helicase C domain 1
IFIT1	12.2	4.1	9.9	6.0	4.8	5.6	4.8	interferon-induced protein with tetratricopeptide repeats 1
IFIT3	7.1	3.3	6.8	3.8	3.4	3.5	3.9	interferon-induced protein with tetratricopeptide repeats 3
IFIT5	2.7	1.9	2.7	2.4	2.0	2.2	1.8	interferon-induced protein with tetratricopeptide repeats 5
IFITM1	2.0	1.6	1.9	1.7	1.7	1.7	1.7	interferon induced transmembrane protein 1 (9–27)
IFITM3	2.2	1.8	2.1	1.8	1.7	1.8	2.0	interferon induced transmembrane protein 3 (1-8U)
IRF7	3.7	2.4	3.3	2.5	2.2	2.5	2.4	interferon regulatory factor 7
ISG15	6.8	3.8	6.6	4.0	3.9	4.4	4.2	ISG15 ubiquitin-like modifier
ISG20	2.5	1.8	2.6	1.8	1.7	1.6	1.7	interferon stimulated exonuclease gene 20kDa
LAMP3	6.0	3.1	6.7	3.3	3.8	3.9	4.4	lysosomal-associated membrane protein 3
LGALS3BP	4.0	2.2	4.0	2.8	2.6	3.0	3.1	lectin, galactoside-binding, soluble, 3 binding protein
LOC26010	4.3	2.5	4.2	3.0	3.0	3.1	3.3	viral DNA polymerase-transactivated protein 6
LOC391020	2.7	2.0	2.7	2.2	2.1	2.2	2.5	interferon induced transmembrane protein pseudogene
LY6E	4.1	2.7	4.0	3.1	2.8	3.0	3.4	lymphocyte antigen 6 complex, locus E
MX1	5.0	3.5	4.9	3.8	3.7	3.9	4.1	myxovirus (influenza virus) resistance 1, interferon-inducible protein p78 (mouse)
MX2	3.1	2.1	2.9	2.2	2.2	2.3	2.4	myxovirus (influenza virus) resistance 2 (mouse)
N4BP1	1.5	1.5	1.7	1.6	1.6	1.7	1.6	Nedd4 binding protein 1
OAS1	4.3	2.4	3.8	2.5	2.1	2.3	2.3	2',5'-oligoadenylate synthetase 1, 40/46kDa
OAS2	3.5	2.4	3.4	2.9	2.4	2.5	2.5	2'-5'-oligoadenylate synthetase 2, 69/71kDa
OAS3	4.5	2.6	3.8	2.7	2.3	2.5	2.8	2'-5'-oligoadenylate synthetase 3, 100kDa
OASL	4.6	2.1	4.1	2.7	2.2	2.5	2.6	2'-5'-oligoadenylate synthetase-like
PARP12	2.4	1.8	2.3	1.8	1.8	1.9	1.9	poly (ADP-ribose) polymerase family, member 12
PHF11	1.8	1.6	1.8	1.6	1.7	1.7	1.7	PHD finger protein 11
PLSCR1	3.1	1.9	2.8	2.1	2.1	2.1	2.4	phospholipid scramblase 1
RNASE2	2.9	1.9	2.3	*1.8*	1.7	1.7	1.9	ribonuclease, RNase A family, 2 (liver, eosinophil-derived neurotoxin)
RSAD2	12.8	5.3	11.6	6.8	6.4	6.4	6.6	radical S-adenosyl methionine domain containing 2
SAMD9	3.8	2.1	3.3	2.6	2.1	2.1	2.0	sterile alpha motif domain containing 9
SERPING1	4.9	1.9	4.1	2.3	1.9	2.0	2.1	serpin peptidase inhibitor, clade G (C1 inhibitor), member 1, (angioedema, hereditary)
SIGLEC1	27.1	16.5	23.1	17.0	13.7	18.2	20.9	sialic acid binding Ig-like lectin 1, sialoadhesin
SP100	1.9	1.7	1.9	1.6	1.6	1.5	1.8	SP100 nuclear antigen
SP110	1.9	*1.5*	1.9	1.5	1.5	1.5	1.6	SP110 nuclear body protein
USP18	7.4	4.9	7.5	5.1	4.5	5.3	5.5	ubiquitin specific peptidase 18
								
XAF1	4.3	3.3	3.8	2.9	2.9	2.5	3.1	XIAP associated factor-1
ZBP1	3.0	2.2	3.0	2.2	2.1	2.2	2.2	Z-DNA binding protein 1
ZCCHC2	2.3	1.9	2.2	1.7	1.8	1.8	1.8	zinc finger, CCHC domain containing 2
cDNA CSODK002YF13	3.3	2.6	3.4	2.6	2.5	2.7	2.8	Full-length cDNA clone CS0DK002YF13 of HeLa cells Cot 25-normalized of Homo sapiens (human)
cDNA FLJ11754	3.1	2.4	3.1	2.3	2.6	2.6	2.6	CDNA FLJ11754 fis, clone HEMBA1005588
**Down regulated**								
ALDH1A1	*-1.5*	-1.7	-1.7	-2.0	-2.5	-2.5	-2.6	aldehyde dehydrogenase 1 family, member A1
CDKN1C	1.9	-2.1	*-1.1*	-1.7	-1.7	-1.7	-1.7	cyclin-dependent kinase inhibitor 1C (p57, Kip2)
EIF3EIP	-2.0	-1.6	-2.0	-1.8	-1.6	-1.6	-1.7	eukaryotic translation initiation factor 3, subunit E interacting protein
EIF4B	-2.0	*-1.5*	-2.0	-1.6	-1.5	-1.9	-1.8	eukaryotic translation initiation factor 4B
FCER1A	*-1.5*	-1.7	-2.1	-2.2	-2.4	-3.0	-2.8	Fc fragment of IgE, high affinity I, receptor for; alpha polypeptide
INSR	*-1.5*	-1.6	-1.6	-1.9	-2.0	-2.0	-1.6	insulin receptor
LTA4H	-1.7	-1.7	-2.0	-1.7	-1.6	-1.6	-1.6	leukotriene A4 hydrolase
PAPSS2	-1.7	-1.7	-1.8	-1.6	-1.6	*-1.3*	-1.6	3'-phosphoadenosine 5'-phosphosulfate synthase 2
PID1	-2.0	-1.8	-2.3	-1.8	-1.8	-1.9	-1.9	phosphotyrosine interaction domain containing 1
RTN1	-2.2	-1.9	-2.3	-2.0	-2.0	-2.0	-2.3	reticulon 1

Values in italics are not significant.

**Figure 2:**
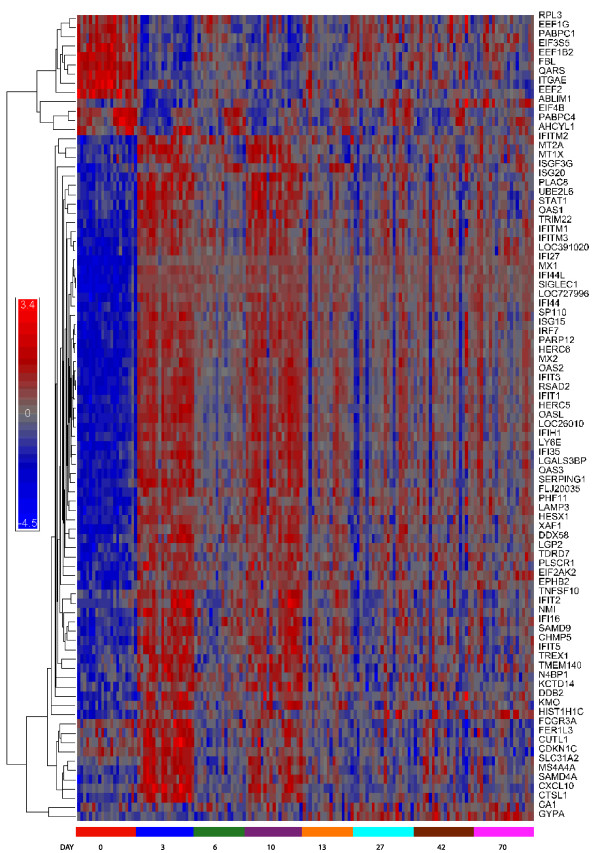
**To illustrate the pattern of expression across time-points, data from the top 90 genes (by p-value) across all time points were clustered by Pearson Dissimilarity.** Arrays (horizontal axis) are arranged in the order of time point (day) and within each time point by Non-Responder(NR) and Responder (R) as determined by viral titer at week 72 (final response in table 2). Expression values were normalized after clustering.

There is a strong pattern of gene expression as a function of time, as demonstrated by hierarchical clustering of the 90 genes that differed most (Figure 2). There is a clearly visible, alternating pattern of increases and decreases that decays over time. The patterns of gene expression can be divided into four groups. The top cluster are genes that are decreased as a result of treatment. These include genes associated with protein synthesis including eukaryotic initiation and elongation factors *(EIF4B, EEI2, EIF3S5) *and genes involved in ribosomal proteins (*RPL3*). The majority of genes fall into a second group, highly induced at days 3 and 10 but showing a decrease at day 6 and 13; the alternation decreases with time but is still high at day 70. This includes most of the well characterized IFN inducible genes, including *MX1, MX2, OAS1, OAS2, OAL, RIG1 (DDX58) *and most interferon stimulated genes (ISGs). A third group are transiently induced genes, i.e. genes induced at day 3 and then returning to base line at later times (Table 5); many have been described as important in the interferon antiviral response and include *CXCL10, IL1RA (IL1RN), JAK2, TNFSF10 (TRAIL)*, as well as *CDKN1C, CXCL10, SMD4A*. The last two genes at the bottom of the cluster array represent genes that are induced late. As is obvious for *GYPA *(glycophorin A), induction for such genes begins around day 27 and proceeds through day 70. Most of the genes in this latter group are related to blood chemistry, including hemoglobin complex formation, heme binding and oxygen transport (Table 6), which may reflect secondary response to long term treatment with ribavirin. A more complete list of genes in each category is presented in the accompanying Tables 4, 5 and 6.

**Table 5:** Transiently induced genes

Symbol	Fold Change	Description
ABCA1	1.8	ATP-binding cassette, sub-family A (ABC1), member 1
ABCC3	1.7	ATP-binding cassette, sub-family C (CFTR/MRP), member 3
AIM2	1.6	Related to IFI16
ARRB1	1.7	arrestin, beta 1
C1QA	1.6	complement component 1, q subcomponent, A chain
C1QB	2.0	complement component 1, q subcomponent, B chain
CALML4	1.8	calmodulin-like 4
CDKN1C	2.2	cyclin-dependent kinase inhibitor 1C (p57, Kip2)
CMKLR1	2.0	chemokine-like receptor 1, chimerin
CTSL1	2.4	cathepsin L1 (lysosomal cysteine proteinase)
CUTL1	1.5	cut-like 1, CCAAT displacement protein (Drosophila)
CXCL10	4.0	chemokine (C-X-C motif) ligand 10
FAM46A	1.6	family with sequence similarity 46, member A
FFAR2 (GPR43)	2.7	free fatty acid receptor 2.
hCG_1776259	1.9	hypothetical protein FLJ23556 (unknown function)
IL1RN	2.1	interleukin 1 receptor antagonist
JAK2	1.7	Janus kinase 2 (a protein tyrosine kinase)
LILRA3	1.6	leukocyte immunoglobulin-like receptor, subfamily A (without TM domain), member 3
MARCKS	2.4	myristoylated alanine-rich protein kinase C substrate
RHOB	1.7	ras homolog gene family, member B
RRAS	1.9	related RAS viral (r-ras) oncogene homolog
SAMD4A	2.6	sterile alpha motif domain containing 4A
SLC31A2	1.7	solute carrier family 31 (copper transporters), member 2
TCN2	2.2	transcobalamin II; macrocytic anemia
TLR1	1.6	toll-like receptor 1
TNFAIP6 (TSG-6)	2.1	tumor necrosis factor, alpha-induced protein 6
TNFSF10 (TRAIL)	2.3	tumor necrosis factor (ligand) superfamily, member 10
TRAFD1 (FLN29)	2.0	TRAF-type zinc finger domain containing 1
VDR	1.6	vitamin D (1,25- dihydroxyvitamin D3) receptor

Genes are significantly induced at day 3 but either not induced at any other time point, or day 3 fold change is at least 20% higher than other days.

**Table 6:** Genes induced late

Symbol	Day 3	Day 6	Day 10	Day 13	Day 27	Day 42	Day 70	Description
CA1	*-1.4*	*1.1*	*3.8*	*8.9*	*13.9*	*15.2*	21.2	carbonic anhydrase I
FKBP8	*1.2*	*-1.1*	*1.4*	*2.7*	*2.1*	*2.7*	3.5	FK506 binding protein 8, 38kDa
GYPA	*1.5*	*1.3*	*4.5*	*8.4*	*14.7*	*15.5*	17.3	glycophorin A (MNS blood group)
GYPB	*-1.1*	*-1.1*	*1.7*	*2.9*	*4.8*	*4.4*	5.0	glycophorin B (MNS blood group)
GYPB	*1.2*	*-1.0*	*2.4*	*4.5*	*6.0*	*6.5*	7.5	glycophorin B (MNS blood group)
GYPB///GYPE	*-1.1*	*-1.0*	*1.5*	*2.7*	*3.2*	*3.3*	3.7	glycophorin B (MNS blood group)///glycophorin E
HBD	*-1.1*	*-1.0*	*2.8*	*4.8*	7.6	*7.0*	9.4	hemoglobin, delta
HBG1///HBG2	*-1.2*	*-1.2*	*1.6*	*2.5*	4.7	4.7	6.5	hemoglobin, gamma A///hemoglobin, gamma G
HBG2	*-1.2*	*-1.3*	*1.8*	*2.5*	5.0	5.0	6.8	hemoglobin, gamma G
HIST1H3H	*1.5*	*1.5*	2.0	*1.4*	1.8	1.9	2.8	histone cluster 1, H3h
LCN2	*1.8*	*1.3*	2.6	*1.9*	1.8	*1.8*	2.8	lipocalin 2 (oncogene 24p3)
MYL4	*1.0*	*1.1*	*1.4*	*1.9*	2.5	*2.5*	3.2	myosin, light chain 4, alkali; atrial, embryonic
MYL4	*-1.0*	*-1.1*	*1.4*	*2.2*	*2.9*	*3.2*	3.7	myosin, light chain 4, alkali; atrial, embryonic
SLC14A1	*-1.2*	*1.1*	*1.2*	*1.9*	*1.9*	*2.1*	2.2	solute carrier family 14 (urea transporter), member 1 (Kidd blood group)
TAL1	*1.1*	*-1.3*	*1.4*	*1.6*	*2.1*	*1.9*	2.7	T-cell acute lymphocytic leukemia 1
TRIM58	*1.7*	*-1.1*	2.5	*2.6*	4.0	*3.3*	4.2	tripartite motif-containing 58

Values in italics not significant when compared to base line.

**Figure 3:**
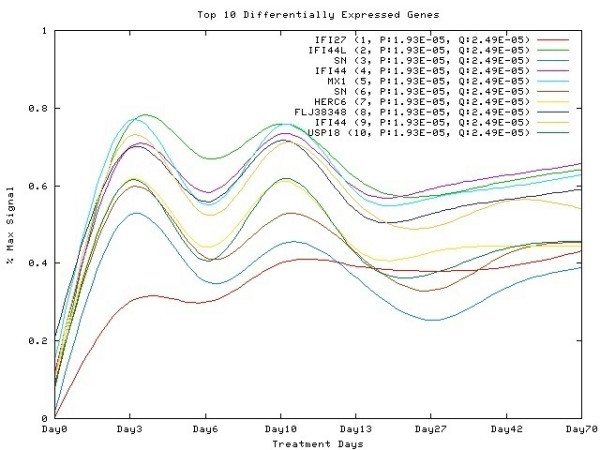
Gene expression profile over time of the 10 genes most differentially expressed, plotted as percentage of maximum signal with points were connected by natural cubic splines.

To further examine the variation of gene expression with time, we used Edge software [28], which tests for changes in gene expression over time *vs*. the null hypothesis that the gene was expressed at a constant level. Among the 518 gene probes that were significantly modulated (absolute fold change ≥ 1.5, p ≤ 0.001) at any one time point in the study (Supplementary Table 1) 90% were shown to be differentially regulated over time (p ≤ 0.001; False Discovery Rate ≤ 0.001) in a cyclic fashion. The ten most differentially expressed of these genes are plotted in Figure 3. These same genes were previously selected by an unbiased mathematical model as being involved in interferon anti-HCV activity [26].

### Comparison with previous studies

To compare the level of induction or down regulation between this study and a previous study (Virahep C; [2]) performed with Peg-intron, we chose twenty patients from the Virahep C study for whom we had data from day 3 (note that day 3 in Virahep C was the fourth day after interferon injection, which was day 0 in that study). The top 20 genes in terms of fold change are shown in Table 7. All genes induced in both trials are presented in Supplementary Table 2. Note that in the Virahep C study the dose of Peginterferon-alfa2a (Pegasys™) was 180 ug; in the present study the dose of Pegylated-interferon-alfa2b (PegIntron™) was lower: 1.5 ug/kg, for an average of 133 ug (standard deviation 25.6, maximum 174).

**Table 7:** Comparison of fold changes between Pegaysy and Peg-intron at day 3

**Gene Symbol**	**Probe Set ID**	**Gene Title**	**Pegasys**^1^	**PegIntron**^2^
IFI27	202411_at	interferon, alpha-inducible protein 27	46.6	73.3
SIGLEC1	219519_s_at	sialic acid binding Ig-like lectin 1, sialoadhesin	15.7	27.1
CCL2	216598_s_at	chemokine (C-C motif) ligand 2	15.1	13.4
RSAD2	213797_at	radical S-adenosyl methionine domain containing 2	11.7	12.8
IFIT1	203153_at	interferon-induced protein with tetratricopeptide repeats 1	11.3	12.2
HESX1	211267_at	HESX homeobox 1	14.7	11.5
IFI44L	204439_at	interferon-induced protein 44-like	6.5	10.1
USP18	219211_at	ubiquitin specific peptidase 18///similar to ubiquitin specific peptidase 18	5.6	7.4
IFIT3	204747_at	interferon-induced protein with tetratricopeptide repeats 3	7.6	7.1
IFI44	214059_at	Interferon-induced protein 44	4.8	7.1
ISG15	205483_s_at	ISG15 ubiquitin-like modifier	6.2	6.8
SIGLEC1	44673_at	sialic acid binding Ig-like lectin 1, sialoadhesin	5.5	6.4
LAMP3	205569_at	lysosomal-associated membrane protein 3	4.1	6.0
IFI44	214453_s_at	interferon-induced protein 44	4.5	6.0
HERC6	219352_at	hect domain and RLD 6	4.2	5.9
MX1	202086_at	myxovirus (influenza virus) resistance 1, interferon-inducible protein p78 (mouse)	4.3	5.0
SERPING1	200986_at	serpin peptidase inhibitor, clade G (C1 inhibitor), member 1, (angioedema, hereditary)	4.0	4.9
OASL	205660_at	2'-5'-oligoadenylate synthetase-like	4.9	4.6
HERC5	219863_at	hect domain and RLD 5	4.4	4.5
OAS3	218400_at	2'-5'-oligoadenylate synthetase 3, 100 kDa	4.1	4.5

^1^Fold-changes in Virahep C study []

^2^Fold-changes in this study.

## Discussion

The aim of this study was to examine the effects of Peg interferon alfa-2b (PegIntron™, administered at 1.5 ug/kg) plus ribavirin on gene expression as a function of time in a cohort of patients infected with HCV genotype 1. The number of genes modified and the signal values for each individual gene induced or down regulated as a result of interferon treatment are remarkably similar between this study and others [2,30,31]. Virtually all genes identified as being important in the interferon response were induced approximately equally in this study and the Virahep C clinical trial [2,23], despite the different interferon used (PegIntron™ here *vs*. Pegasys™ in Virahep C) and the difference in dose (Table 7 and Supplementary Table 2). The few apparent differences were on the borderline of being significant or close to the 1.5 fold cutoff we chose. The kinetics of gene induction was also very similar, with most genes being induced early and elevated throughout the trial period. This was true despite several differences between the studies. Virahep C study used peg interferon α-2a (Pegasys™), whereas here we used peg interferon α-2b (PegIntron™). Another difference was the dose of interferon used; the Virahep C study used a constant amount (180 μg/injection), whereas here we adjusted dose based upon the initial weight of the patient (1.5 μg/kg). A third difference was that subjects for the Virahep C gene expression study were selected based on their viral titer response during the first 28 days of treatment, to allow comparison among fast responders, slow responders and non-responders. In the present study, patients were not selected for response, but rather all subjects were analyzed, and only 3 subjects in the current study would have met the Virahep C criteria of rapid responders; this greatly reduced our power to detect differences in gene expression related to response. Considering that these trials were done a few years apart, and with different populations, there was excellent agreement in the changes in gene expression. Some of the small differences seen are due to heterogeneity within the populations, and are apparent even at day 1, before initiation of treatment. For instance, the mean weight of this 20 person cohort was 88.4 kg which correlates with the those in the Virahep C cohort having intermediate or poor response, and 11/20 individuals had bridging fibrosis or cirrhosis. In addition, there was just one African American in this cohort.

As can be seen from Figure 1 and Table 3, and Supplementary table 1 a large number of genes are initially induced following treatment. In the earlier study, the peak was at day 1 after treatment [2], however this time point was not included in the present study; thus in the present study the peak was observed at the earliest time point after injection, day 3. In general, there was very good agreement in the fold increase in gene expression at day 3 (Table 7 and Supplementary Table 2). As in the case of Virahep C, there is a decline in both the number of genes induced and the extent of gene elevation before the next injection of interferon, at day 6 here and day 7 in the Virahep C study. At that time there is a small increase in viral titer. This pattern appears to be repeated until about a month into the study (Table 3, Figures 1, 2 and 3), which might suggest that interferon treatment more frequently than once a week would be more efficacious in early stages of treatment.

Among the major functional categories of genes induced at day 3 (based on Gene Ontology categories and KEGG pathways) are innate immune response, transcription factors, and chemotaxis. Many of these genes have previously been reported to be induced primarily by IFN-gamma, suggesting low amounts of IFN-gamma may be induced, although we could not detect IFN-gamma in our arrays.

We have divided the gene responses into four categories: genes that are induced early and once induced are induced throughout the trial period, genes transiently induced, those that appear late and down regulated genes (Tables 4, 5, 6). Most of these genes fall into a similar temporal category in the previous study [2]. Most of the genes that are induced (or down regulated) throughout the studied period (up to 10 weeks; Table 4 and Supplementary Table 1) were previously identified as being involved in the interferon response [2,22-25,32]. Among gene functions significantly altered by IFN are genes involved in the immune response including inflammation, genes previously reported to be involved in response to virus infection and transcription factors (DNA and RNA binding proteins).

Among the genes transiently expressed is *CXCL10*. It has been proposed previously that the levels of this gene are related to the final outcome of treatment [33,34]. However both in this trial and in the Virahep C trial, this gene is only expressed at enhanced levels for the first few days after initiation of IFN treatment, and by day 13 is back to baseline levels. We have not found a correlation between *CXCL10 *expression and response to treatment. Protein levels (ELISA data not shown) follow the mRNA levels. IL1ra (*IL1RN*) has previously been reported to be transiently expressed at the protein level following interferon treatment [35], and, as can be seen from Table 5, this is confirmed in the microarray data.

*TNFSF10 *(tumor necrosis factor (ligand) superfamily, member 10, TRAIL), a gene associated with apoptosis in transformed and tumor cells [36] and recently shown to have direct anti-viral activity against dengue virus [37], is induced early but only transiently. This gene was shown to be induced at high levels during the early stages of treatment in our previous study [2]. It is possible that it has direct anti-hepatitis C activity. TNFAIP6 (TSG6) is also induced early; its gene product has been show to have anti-inflammatory activity and may inhibit TNF activity by a feed back loop [38,39].

*AIM2 *has been identified as part of a cluster of homologous genes (*MNDA*, *IFI16 *and *AIM2*) on human chromosome 1 [40] referred to as IFI or HIN-200 genes. It has been suggested that *AIM2 *functions as a tumor suppressor gene [41], however, over expression of *AIM2 *in another study did not induce a tumor suppressor phenotype [42]. *AIM2 *has homology to *IFI16*. However, whereas *IFI16 *is induced and highly expressed throughout the treatment period, *AIM2 *is not, indicating that the regulation of this gene differs from that of other HIN-200 family members.

*TLR1 *and *FLN29 *(*TRAFD1*), regulators of toll like receptor signaling [43], are both transiently induced. *TLR1 *is involved in recognition of viral antigens, and is found on the surface of most immune cells. On the other hand, *TLR7 *is induced through out the 10 week period.

*CDKN1C *(cyclin dependent kinase inhibitor 1C, alias p57, Kip2) behaves differently from all the other genes. The mRNA for this gene is elevated early, both in this trial and in the Virahep C trial, but is severely repressed at later times rather than returning to base line. This gene product is an inhibitor of several G1 cyclin/CdK complexes and a negative regulator of cell proliferation. *CDKN1C *plays a role in cell proliferation, differentiation, apoptosis, tumorgenesis and developmental changes. It has been reported that the *CDKN1C *protein physically interacts with and inhibits the c-Jun NH2-terminal kinase/stress activated protein kinase (JNK/SAPK) [44]. It has also been reported to bind to the proliferating cell nuclear antigen (PCNA), and thus control the cell cycle [45]. This is the first report that this gene is regulated by interferon or ribavirin. Its role in the interferon/ribavirin response is unknown.

### Late Gene Induction

One area very rarely studied is the change in profile of gene induction after a few weeks of treatment with IFN and ribavirin. Most of the late genes probably represent secondary or tertiary events, and include genes involved in hemopoiesis, hemoglobin complex formation, and oxygen transport and binding. Genes for the synthesis of hemoglobin delta and gamma are enhanced. There is no enhanced synthesis of *HBA *or *HBB*, both of which are expressed at high levels. Carbonic anhydrase, which has been associated previously with erythrocytes [46], is also highly induced late in treatment. We noted that this gene was also induced late in patients in the Virahep C study. Its importance in blood chemistry or response to interferon is unknown. Glycophorins A (*GYPA*) and B (*GYPB*) are major sialoglycoproteins of the human erythrocyte membrane which bear the antigenic determinants for the MN and Ss blood groups [47]. The enhanced synthesis of these genes also indicates changes in the synthesis of erythrocytes. These changes may reflect the side effects of interferon or more likely ribavirin therapy. The major clinical side effect of ribavirin is a hemolytic anemia [48,49], and thus the changes in expression of these genes may represent compensatory responses.

### Patient Variation

In both this study, and our previous one [2], we noted considerable variability in the initial levels of gene expression among subjects. Thus both studies were designed to examine the changes from this baseline; such a design allowed robust detection of the effects of interferon, and as noted above most changes were consistent between the two studies (Table 7) The clinical results of this trial are similar to previously reported trials in genotype I naïve patients [1,2,7,8,50]. The sustained viral response rate (SVR) was approximately 35%. Only three patients showed a rapid response to interferon/ribavirin therapy, with an immediate decrease in viral titer. Ten patients showed no decrease in virus titer, and 7 showed a biphasic decrease. However the rate of initial viral decrease for the 7 patients with a biphasic curve was not predictive of final response in this small study. Two patients showed a return of virus at week 42 although one had cleared at week 10 the other by week 12. Perhaps HCV can normally interfere with the interferon response through binding to RIG-I [51,52] and the mutated virus is the exception and sensitive to interferon treatment. This is supported by recent work in which it has been shown there is more variability in RNA sequence in virus from responders than in non-responders[53]. The occurrence of relapsers could be due to reversion of such " mutated" virus to wild type and restoration of resistance to treatment. The presence of relapsers also indicates that virus is "hiding" in either the liver or cells of the immune system or is present at non-detectable levels in the serum.

## Conclusion

We have used multiple time points to measure gene expression. It is obvious from our data that a single measurement might give misleading data on gene expression and thus mechanism of action of the drug combination. This can be seen in particular with genes that are only transiently expressed, or that vary during treatment. Because both this study and the earlier Virahep C study [2] show a peak in the effects of interferon within the first 3 days after interferon, and a decline toward baseline before the next injection, it suggests that treatment more frequently than once a week, at least during the first month of treatment, might be more efficacious. The effects of more frequent treatment could be measured using the responses of a few key genes as a function of time. However it is also possible that the receptor sites are down regulated and require some time to be reactivated [54] or resynthesized. No major differences were found in gene induction or down regulation patterns between this study and that of Virahep C. Thus the location of the pegylation and structure of the interferon does not appear to be important *in vivo*, although it does alter the anti-viral activity *in vitro *[14]. This could suggest that the receptor sites for interferon are saturated *in vivo*, and that the activities once bound to the receptor are identical. It should be noted that the patients treated in this study received lower doses of interferon. We could find no relationship between response to therapy and gene induction in this trial, perhaps because of the very low number of rapid and sustained responders.

## Competing interests

Dr. Paul Kwo is a Scientific Advisor to Schering- Plough.

## Authors' contributions

This Ms was written and data interpreted by MWT. TT was employed as a bio-informaticist and together with JNM performed statistical analysis. HJE as head of the Center for Medical Genomics helped write the manuscript. PK ran the clinical study.

## Supplementary Material

**Additional file 1 ** **List of genes with significant differential expression for at least one time point** Click here for file

**Additional file 2 ** **Comparison of fold changes for genes differentially expressed at day 3 for this study and the ViraHep C study** Click here for file
