# Supplementary material for: Cyclic changes in gene expression induced by Peg-interferon alfa-2b plus ribavirin in peripheral blood monocytes (PBMC) of hepatitis C patients during the first 10 weeks of treatment
Source: J Transl Med. 2008 Nov 5;6:66. doi: 10.1186/1479-5876-6-66 (PMC2613871; doi:10.1186/1479-5876-6-66)
Supplement: Additional file 1 — List of genes with significant differential expression for at least one time point [file 1479-5876-6-66-S1.pdf]

Genes with significant differential expression for at least one time point.

			Day 1	Day 3		Day 6		Day 10		Day 13		Day 27		Day 42		Day 70	
Gene Symbol	Probe Set ID	Gene Title	mean signal	Fold Chg	p-value	Fold Chg	p-value	Fold Chg	p-value	Fold Chg	p-value	Fold Chg	p-value	Fold Chg	p-value	Fold Chg	p-value
ABCA1	203505_at	ATP-binding cassette, sub-family A (ABC1), member 1	359	1.75	0.00006	1.18	0.16047	1.37	0.00645	1.13	0.18533	1.15	0.17553	1.12	0.29663	1.29	0.03224
ABCC3	208161_s_at	ATP-binding cassette, sub-family C (CFTR/MRP), member 3	432	1.66*	0.00006	ND		ND		ND		ND		ND		ND	
ABHD2	63825_at	abhydrolase domain containing 2	997	-1.29	0.00497	-1.19	0.03054	-1.47	0.00001	-1.51	0.00030	-1.64	0.00016	-1.61	0.00001	-1.75	0.00001
ADAR	201786_s_at	adenosine deaminase, RNA-specific	7548	1.68	0.00000	1.5	0.00000	1.64	0.00000	1.47	0.00000	1.43	0.00000	1.38	0.00019	1.42	0.00000
ADK	204120_s_at	adenosine kinase	162	-1.54	0.00007	-1.33	0.01659	-1.27	0.01893	-1.39	0.01355	-1.14	0.21761	-1.14	0.28551	-1.42	0.00116
ADM	202912_at	adrenomedullin	770	1.84	0.00097	1.27	0.12805	1.47	0.02847	1	0.98585	1.28	0.07742	1.13	0.31173	1.55	0.00139
AGRN	212285_s_at	agrin	432	2.11	0.00000	1.69	0.00016	2.07	0.00000	1.86	0.00000	1.59	0.00003	1.73	0.00004	2.14	0.00000
AHR	202820_at	aryl hydrocarbon receptor	609	-1.26	0.02184	-1.27	0.02097	-1.34	0.01917	-1.48	0.00341	-1.56	0.00052	-1.29	0.01896	-1.63	0.00021
AIM2	206513_at	absent in melanoma 2	1078	1.64	0.00000	-1.04	0.57488	1.18	0.01329	-1.06	0.43730	-1.06	0.45846	-1.02	0.71671	-1.07	0.45239
AKAP2 /// PALM2-AKAP2	202760_s_at	A kinase (PRKA) anchor protein 2 /// PALM2-AKAP2 protein	711	1.53	0.00010	1.42	0.00023	1.74	0.00003	1.33	0.00679	1.45	0.00081	1.17	0.17255	1.53	0.00002
ALDH1A1	212224_at	aldehyde dehydrogenase 1 family, member A1	729	-1.45	0.00001	-1.72	0.00000	-1.74	0.00000	-2.03	0.00000	-2.48	0.00000	-2.47	0.00000	-2.57	0.00000
ALDH2	201425_at	aldehyde dehydrogenase 2 family (mitochondrial)	6406	-1.45	0.00000	-1.46	0.00000	-1.55	0.00000	-1.48	0.00000	-1.52	0.00001	-1.52	0.00004	-1.45	0.00001
AMY1A /// AMY1B /// AMY1C /// AMY2A /// AMY2B /// LOC730924	208498_s_at	amylase, alpha 1A (salivary) /// alpha 1B (salivary) /// alpha 1C (salivary) /// alpha 2A (pancreatic) /// alpha 2B (pancreatic) /// similar to Pancreatic alpha-amylase precursor (PA) (1,4-alpha-D-glucan glucanohydrolase)	407	1.05	0.35348	1.52	0.00082	1.22	0.05446	1.17	0.18637	1.26	0.00578	1.27	0.06444	1.21	0.12182
ANP32A	201043_s_at	acidic (leucine-rich) nuclear phosphoprotein 32 family, member A	575	-1.08	0.41214	-1.07	0.52163	-1.3	0.03065	-1.21	0.14439	-1.44	0.00388	-1.34	0.02000	-1.73	0.00004
APOBEC3A	210873_x_at	apolipoprotein B mRNA editing enzyme, catalytic polypeptide-like 3A	2075	2.94	0.00000	2.01	0.00001	2.72	0.00000	1.88	0.00103	1.87	0.00011	1.81	0.00009	2	0.00000
ARRB1	218832_x_at	arrestin, beta 1	350	1.68*	0.00008	ND		ND		ND		ND		ND		ND	
ASGR1	206743_s_at	asialoglycoprotein receptor 1	478	-1.08	0.23139	-1.19#	0.02344	-1.18#	0.04365	-1.31#	0.00360	-1.56#	0.00003	-1.37#	0.01383	-1.41#	0.00072
ATG12	204833_at	ATG12 autophagy related 12 homolog (S. cerevisiae)	205	1.93*	0.00002	1.58*	0.00012	ND		ND		ND		ND		ND	
ATOX1	203454_s_at	ATX1 antioxidant protein 1 homolog (yeast)	909	1.58*	0.00000	1.12*	0.12292	1.47*	0.00190	1.13*	0.17205	ND		1.24*	0.02927	1.18*	0.03486
ATP10A	214255_at	ATPase, Class V, type 10A	447	1.43	0.00189	1.39	0.00500	1.47	0.00030	1.48	0.00005	1.5	0.00014	1.66	0.00042	1.4	0.00304
ATP6V1B2	201089_at	ATPase, H+ transporting, lysosomal 56/58kDa, V1 subunit B2	6801	-1.23	0.01018	-1.43	0.00054	-1.37	0.00105	-1.27	0.00003	-1.65	0.00000	-1.42	0.00008	-1.82	0.00000
ATXN1	203231_s_at	ataxin 1	656	-1.58	0.00004	-1.08	0.20372	-1.31	0.00049	-1.2	0.04337	-1.26	0.07173	-1.15	0.20957	-1.2	0.08740
AXL	202686_s_at	AXL receptor tyrosine kinase	192	ND		ND		2.36*	0.00000	ND		ND		ND		ND	
BCAT2	203576_at	branched chain aminotransferase 2, mitochondrial	535	-1.31#	0.00241	-1.27#	0.00908	-1.65#	0.00005	-1.18	0.06991	-1.29#	0.00872	-1.22#	0.10360	-1.56#	0.00025
BICD2	212702_s_at	bicaudal D homolog 2 (Drosophila)	1493	-1.25	0.00026	-1.2	0.03693	-1.28	0.00086	-1.28	0.02563	-1.51	0.00001	-1.47	0.00046	-1.48	0.00097
BID	204493_at	BH3 interacting domain death agonist	1429	1.09	0.16140	-1.14	0.07514	-1.19	0.01781	-1.29	0.00477	-1.38	0.00007	-1.38	0.00005	-1.54	0.00000
BLVRA	203771_s_at	biliverdin reductase A	591	1.63	0.00000	1.35	0.00077	1.74	0.00000	1.33	0.04107	1.33	0.00430	1.37	0.00428	1.43	0.00015
BLVRA	211729_x_at	biliverdin reductase A	4388	1.61	0.00000	1.2	0.04307	1.63	0.00003	1.34	0.01107	1.34	0.00366	1.39	0.00650	1.37	0.00038
BLVRA	203773_x_at	biliverdin reductase A	4704	1.55	0.00003	1.15	0.20750	1.52	0.00050	1.28	0.09852	1.19	0.11865	1.3	0.04536	1.19	0.13127
BST2	201641_at	bone marrow stromal cell antigen 2	2224	2.06	0.00000	1.59	0.00000	1.96	0.00000	1.6	0.00007	1.41	0.00138	1.55	0.00128	1.5	0.00015
C11orf2	217969_at	chromosome 11 open reading frame2	5350	-1.63	0.00000	-1.43	0.00000	-1.68	0.00000	-1.42	0.00001	-1.32	0.00005	-1.3	0.00022	-1.44	0.00001
C14orf147	213508_at	chromosome 14 open reading frame 147	1049	-1.34	0.00122	-1.41	0.00038	-1.54#	0.00007	-1.34	0.00225	-1.58#	0.00000	-1.45#	0.00029	-1.75#	0.00001
C1GALT1	219439_at	core 1 synthase, glycoprotein-N-acetylgalactosamine 3-beta-galactosyltransferase, 1	425	1.55	0.00000	1.35	0.00002	1.48	0.00001	1.34	0.00003	1.31	0.00000	1.31	0.00006	1.31	0.00008
C1QA	218232_at	complement component 1, q subcomponent, A chain	381	1.63*	0.00048	ND		1.35*	0.02319	ND		ND		ND		1.10*	0.25983
C1QB	202953_at	complement component 1, q subcomponent, B chain	269	1.97*	0.00000	ND		ND		ND		ND		ND		ND	
C2	203052_at	complement component 2	389	1.66*	0.00000	1.14*	0.21241	1.46*	0.00022	1.14*	0.14268	1.08*	0.40375	1.20*	0.09251	1.18*	0.01252
C2orf47	219176_at	chromosome 2 open reading frame 47	851	1.71	0.00000	1.41	0.00014	1.72	0.00000	1.54	0.00000	1.32	0.00025	1.27	0.00202	1.34	0.00234
C3AR1	209906_at	complement component 3a receptor 1	2635	2.27	0.00001	1.3	0.09530	1.92	0.00240	1.39	0.03361	1.11	0.41143	1.31	0.07757	1.16	0.31333
C4orf18	219872_at	chromosome 4 open reading frame 18	419	-1.32	0.00813	-1.31	0.05437	-1.39	0.01428	-1.59	0.00315	-1.72	0.00049	-1.53	0.01376	-1.95	0.00044
C9orf40	218904_s_at	chromosome 9 open reading frame 40	155	1.15*	0.16058	1.11*	0.28196	1.27*	0.02975	1.80*	0.00039	1.47*	0.00856	1.56*	0.00990	1.42*	0.07017
CA1	205950_s_at	carbonic anhydrase I	679	-1.41	0.08929	1.11	0.62325	3.83	0.00466	8.88	0.04929	13.87	0.00135	15.16	0.00727	21.24	0.00074
CALML4	221879_at	calmodulin-like 4	1012	1.72*	0.00000	ND		1.34*	0.00241	ND		ND		ND		ND	
CALML4	64408_s_at	calmodulin-like 4	223	1.83	0.00001	1.03	0.60859	1.4	0.00131	1.01	0.86013	1.04	0.67167	1.02	0.89067	1.12	0.25042
CAPN3	214475_x_at	calpain 3, (p94)	301	1.49*	0.00024	1.54*	0.00025	1.37*	0.01667	ND		ND		ND		ND	
CBR1	209213_at	carbonyl reductase 1	410	1.73	0.00009	1.21	0.15141	1.58	0.00015	1.48	0.00354	1.07	0.60176	1.19	0.23581	1.12	0.42149
CCNG2	202770_s_at	cyclin G2	793	-1.18	0.03832	-1.21	0.03053	-1.26	0.02758	-1.21	0.02549	-1.32	0.00386	-1.26	0.03819	-1.59	0.00028
CCR1	205099_s_at	chemokine (C-C motif) receptor 1	940	2.06	0.00000	1.42	0.00804	2.04	0.00000	1.74	0.00006	1.43	0.00456	1.5	0.00162	1.45	0.00233
CCR1	205098_at	chemokine (C-C motif) receptor 1	2924	1.89	0.00001	1.26	0.15310	1.83	0.00094	1.75	0.00014	1.21	0.02645	1.54	0.00115	1.16	0.33288

			Day 1	Day 3		Day 6		Day 10		Day 13		Day 27		Day 42		Day 70	
Gene Symbol	Probe Set ID	Gene Title	mean signal	Fold Chg	p-value	Fold Chg	p-value	Fold Chg	p-value	Fold Chg	p-value	Fold Chg	p-value	Fold Chg	p-value	Fold Chg	p-value
CCR2 /// LOC729230	206978_at	chemokine (C-C motif) receptor 2 /// similar to C-C chemokine receptor type 2 (C-C CKR-2) (CC-CKR-2) (CCR-2) (CCR2) (Monocyte chemoattractant protein 1 receptor) (MCP-1-R) (CD192 antigen)	4664	-1.36	0.07241	-1.37	0.09056	-1.36	0.09553	-1.14	0.33118	-1.76	0.00017	-1.25	0.13419	-1.91	0.00714
CD1C	205987_at	CD1c molecule	1300	-2.51#	0.00000	-1.82#	0.00000	-1.89#	0.00000	-1.57#	0.00028	-1.28#	0.00217	-1.48#	0.00006	-1.57#	0.00000
CD2AP	203593_at	CD2-associated protein	549	1.63	0.00000	1.42	0.00005	1.67	0.00001	1.45	0.00089	1.45	0.00000	1.49	0.00000	1.48	0.00000
CD302	203799_at	CD302 molecule	5393	-1.46	0.00007	-1.56	0.00008	-1.57	0.00001	-1.43	0.00019	-1.51	0.00002	-1.37	0.00033	-1.41	0.00005
CD33	206120_at	CD33 molecule	1564	-1.02	0.83604	-1.43	0.00218	-1.36	0.00760	-1.44	0.00234	-1.63	0.00009	-1.35	0.03709	-1.57	0.00080
CD38	205692_s_at	CD38 molecule	994	1.95	0.00000	1.34	0.00082	1.67	0.00005	1.3	0.01166	1.19	0.12365	1.16	0.22909	1.25	0.00448
CD44	212014_x_at	CD44 molecule (Indian blood group)	6681	-1.5	0.00001	-1.37	0.00053	-1.57	0.00000	-1.6	0.00001	-1.42	0.00005	-1.49	0.00003	-1.34	0.00088
CD44	209835_x_at	CD44 molecule (Indian blood group)	8053	-1.56	0.00000	-1.35	0.00068	-1.49	0.00002	-1.52	0.00013	-1.33	0.00012	-1.4	0.00036	-1.39	0.00009
CD93	202878_s_at	CD93 molecule	5776	-1.29	0.00124	-1.41	0.00041	-1.43	0.00043	-1.32	0.00128	-1.52	0.00000	-1.33	0.00846	-1.44	0.00065
CDKN1C	213182_x_at	cyclin-dependent kinase inhibitor 1C (p57, Kip2)	846	1.81	0.00016	-1.97#	0.00015	-1.12	0.34801	-2.07#	0.00035	-1.85	0.00105	-1.90#	0.00419	-2.18#	0.00030
CDKN1C	213348_at	cyclin-dependent kinase inhibitor 1C (p57, Kip2)	2473	1.88	0.00000	-2.11	0.00000	-1.1	0.29000	-1.73	0.00000	-1.69	0.00001	-1.69	0.00006	-1.66	0.00001
CDKN1C	216894_x_at	cyclin-dependent kinase inhibitor 1C (p57, Kip2)	813	2.21	0.00002	-2.44	0.00001	-1.16	0.37159	-1.99	0.00298	-2.08	0.00049	-2.02	0.00294	-2.05	0.00223
CDKN1C	219534_x_at	cyclin-dependent kinase inhibitor 1C (p57, Kip2)	1148	2.2	0.00003	-2.47	0.00001	-1.18	0.24011	-2.03	0.00080	-2.17	0.00034	-1.98	0.00129	-2.19	0.00073
CES1	209616_s_at	carboxylesterase 1 (monocyte/macrophage serine esterase 1)	941	2.06	0.00000	1.27	0.00162	1.91	0.00002	1.47	0.00078	1.33	0.01051	1.5	0.04238	1.54	0.00845
CHKB /// CPT1B	210069_at	choline kinase beta /// carnitine palmitoyltransferase 1B (muscle)	739	1.29	0.00004	1.47	0.00001	1.42	0.00007	1.41	0.00000	1.53	0.00000	1.72	0.01064	1.77	0.00000
CHMP5	219356_s_at	chromatin modifying protein 5	1478	2.78	0.00000	1.93	0.00000	2.63	0.00000	2.03	0.00002	1.88	0.00003	1.8	0.00005	1.92	0.00000
CHMP5	218085_at	chromatin modifying protein 5	692	2.56	0.00000	1.69	0.00003	2.43	0.00000	1.83	0.00004	1.52	0.00005	1.76	0.00001	1.62	0.00003
CHST12	218927_s_at	carbohydrate (chondroitin 4) sulfotransferase 12	1538	1.58	0.00000	1.2	0.00114	1.52	0.00022	1.39	0.00006	1.24	0.01121	1.29	0.00243	1.23	0.01747
CIITA	205101_at	class II, major histocompatibility complex, transactivator	707	-1.74#	0.00000	-1.38#	0.00266	-1.63#	0.00000	-1.43#	0.00141	-1.28#	0.00337	-1.36#	0.00059	-1.45#	0.00001
CLIC5	213317_at	chloride intracellular channel 5	208	-1.79#	0.00038	-1.17	0.33599	-1.3	0.02574	-1.42	0.00828	-1.36	0.00822	-1.44#	0.00865	-1.71#	0.00037
CLU	208792_s_at	clusterin	1057	1.62	0.00005	-1.1	0.41693	1.51	0.00684	-1.12	0.43963	-1.1	0.33045	1	0.97406	1.35	0.02369
CMKLR1	207652_s_at	chemokine-like receptor 1	341	2.03*	0.00000	ND		ND		ND		ND		ND		ND	
CMKLR1	210659_at	chemokine-like receptor 1	349	1.69*	0.00000	ND		1.44*	0.00004	ND		ND		ND		1.40*	0.00007
CNP	208912_s_at	2',3'-cyclic nucleotide 3' phosphodiesterase	1503	1.37*	0.00001	1.10*	0.12872	1.50*	0.00000	1.39*	0.00008	1.24*	0.00068	1.32*	0.00192	1.36*	0.00004
COL6A2	209156_s_at	collagen, type VI, alpha 2	565	-1.08	0.28423	1.05	0.47226	-1.37	0.00189	-1.14	0.17298	-1.54	0.00005	-1.39#	0.02494	-1.27	0.04430
COX15	221550_at	COX15 homolog, cytochrome c oxidase assembly protein (yeast)	239	-1.13	0.25508	-1.56	0.01527	-1.4	0.01383	-1.32	0.04235	-1.53#	0.00084	-1.50#	0.00088	-1.47#	0.00288
CPD	201942_s_at	carboxypeptidase D	475	-1.1	0.10638	-1.24	0.00090	-1.35	0.00091	-1.33	0.02211	-1.53	0.00078	-1.57	0.00058	-1.84	0.00012
CPVL	208146_s_at	carboxypeptidase, vitellogenic-like	4561	-1.37	0.00061	-1.54	0.00004	-1.48	0.00020	-1.43	0.00033	-1.65	0.00000	-1.6	0.00005	-1.53	0.00006
CRIM1	202551_s_at	cysteine rich transmembrane BMP regulator 1 (chordin-like)	154	-1.60#	0.00100	-1.21#	0.02097	-1.30#	0.01241	-1.65#	0.00000	-1.20#	0.06283	-1.23#	0.04054	-1.27#	0.05431
CRTAP	201380_at	cartilage associated protein	1014	-1.52	0.00000	-1.43	0.00000	-1.53	0.00000	-1.39	0.00003	-1.34	0.00003	-1.37	0.00001	-1.49	0.00000
CSF1R	203104_at	colony stimulating factor 1 receptor, formerly McDonough feline sarcoma viral (v-fms) oncogene homolog	6075	1.11	0.09692	-1.36	0.00005	-1.16	0.01038	-1.31	0.00193	-1.5	0.00000	-1.39	0.00014	-1.41	0.00003
CSF3R	203591_s_at	colony stimulating factor 3 receptor (granulocyte)	2554	-1.25	0.01115	-1.3	0.01244	-1.36	0.00009	-1.44	0.00157	-1.72	0.00000	-1.65	0.00006	-1.47	0.00277
CTBP1	213980_s_at	C-terminal binding protein 1	3019	-1.18	0.00609	-1.11	0.17033	-1.24	0.04028	-1.19	0.11318	-1.42	0.00083	-1.17	0.08941	-1.67	0.00000
CTSL1	202087_s_at	cathepsin L1	771	2.4	0.00000	1.31	0.00084	1.82	0.00000	1.25	0.01816	1.32	0.00181	1.4	0.01327	1.45	0.00009
CTSZ	210042_s_at	cathepsin Z	1329	-1.35	0.06234	-1.43	0.04157	-1.6	0.00571	-1.83	0.00096	-1.5	0.01524	-2.31	0.00023	-1.76	0.00098
CUTL1	202367_at	cut-like 1, CCAAT displacement protein (Drosophila)	1156	1.53	0.00001	-1.24	0.01218	1.05	0.58619	-1.23	0.01092	-1.25	0.00061	-1.16	0.09641	-1.21	0.00453
CXCL10	204533_at	chemokine (C-X-C motif) ligand 10	703	3.95	0.00001	-1.13	0.24698	2.39	0.00005	-1.16	0.24142	-1.21	0.11369	-1.03	0.78536	-1.09	0.50500
CXCL5	214974_x_at	chemokine (C-X-C motif) ligand 5	527	-1.02	0.83780	-1.59	0.00008	-1.07	0.32346	-1.68	0.00175	-1.86	0.00019	-1.85	0.00025	-1.4	0.00082
CYP1B1	202435_s_at	cytochrome P450, family 1, subfamily B, polypeptide 1	887	-1.46	0.00004	-1.29	0.00984	-1.53	0.00040	-1.44	0.00108	-1.49	0.00024	-1.41	0.00954	-1.46	0.01437
CYP1B1	202437_s_at	cytochrome P450, family 1, subfamily B, polypeptide 1	1524	-1.38	0.00329	-1.23	0.02353	-1.6	0.00067	-1.31	0.02331	-1.56	0.00019	-1.26	0.06591	-1.51	0.00577
DDB2	203409_at	damage-specific DNA binding protein 2, 48kDa	848	1.82	0.00000	1.35	0.00000	1.54	0.00001	1.31	0.00000	1.3	0.00001	1.5	0.00011	1.44	0.00000
DDX24	200702_s_at	DEAD (Asp-Glu-Ala-Asp) box polypeptide 24	593	1.3	0.02330	1.53	0.00087	1.45	0.00004	1.27	0.02055	1.52	0.00519	1.36	0.06102	1.55	0.00436
DDX58	218943_s_at	DEAD (Asp-Glu-Ala-Asp) box polypeptide 58	809	2.83	0.00000	1.77	0.00000	2.62	0.00000	1.65	0.00001	1.79	0.00003	1.63	0.00002	1.71	0.00000
DGAT1	203669_s_at	diacylglycerol O-acyltransferase homolog 1 (mouse)	529	-1.11	0.17625	-1.37	0.00108	-1.53	0.00020	-1.34	0.00394	-1.36#	0.00121	-1.3	0.05321	-1.53	0.00073
DHX58	219364_at	DEXH (Asp-Glu-X-His) box polypeptide 58	255	2.97*	0.00000	2.17*	0.00000	2.75*	0.00000	2.35*	0.00001	ND		2.45*	0.00000	ND	
DPEP2	219452_at	dipeptidase 2	2087	-1.13	0.03831	-1.27	0.00006	-1.57	0.00000	-1.53	0.00000	-1.5	0.00000	-1.43	0.00003	-1.39	0.00002
DSC2	204751_x_at	desmocollin 2	198	-1.29	0.01506	-1.38	0.00079	-1.3	0.00752	-1.46	0.00006	-1.4	0.00182	-1.37#	0.00158	-1.51	0.00002
ECGF1	217497_at	endothelial cell growth factor 1 (platelet-derived)	1083	2.03	0.00000	1.67	0.00006	1.72	0.00002	1.54	0.00167	1.43	0.00232	1.41	0.00025	1.52	0.00058
ECGF1	204858_s_at	endothelial cell growth factor 1 (platelet-derived)	2627	1.71	0.00000	1.24	0.00379	1.67	0.00002	1.32	0.01047	1.15	0.19014	1.29	0.04971	1.46	0.00076
EEF1B2 /// hCG_19809 /// hCG_1983058	200705_s_at	eukaryotic translation elongation factor 1 beta 2 /// hCG1983058 /// eukaryotic translation elongation factor 1 beta 2-like	27667	-1.67	0.00000	-1.29	0.00000	-1.51	0.00000	-1.39	0.00000	-1.24	0.00012	-1.32	0.00000	-1.39	0.00000
EEF1D	203113_s_at	eukaryotic translation elongation factor 1 delta (guanine nucleotide exchange protein)	12411	-1.5	0.00001	-1.07	0.36102	-1.58	0.00001	-1.55	0.00170	-1.9	0.00000	-1.73	0.00005	-2.16	0.00000

			Day 1	Day 3		Day 6		Day 10		Day 13		Day 27		Day 42		Day 70	
Gene Symbol	Probe Set ID	Gene Title	mean signal	Fold Chg	p-value	Fold Chg	p-value	Fold Chg	p-value	Fold Chg	p-value	Fold Chg	p-value	Fold Chg	p-value	Fold Chg	p-value
EEF2	204102_s_at	eukaryotic translation elongation factor 2	19263	-1.58	0.00000	-1.31	0.00003	-1.51	0.00000	-1.36	0.00000	-1.19	0.00145	-1.31	0.00001	-1.33	0.00001
EEF2	200094_s_at	eukaryotic translation elongation factor 2	19261	-1.88	0.00000	-1.52	0.00000	-1.79	0.00000	-1.41	0.00052	-1.4	0.00001	-1.57	0.00000	-1.58	0.00000
EHBP1L1 /// MGC15523	91703_at	hypothetical protein MGC15523 /// EH domain binding protein 1-like 1	1364	-1.1	0.17847	-1.07	0.33105	-1.23	0.02427	-1.41	0.00029	-1.64	0.00000	-1.51	0.00026	-1.55	0.00000
EIF2AK2	204211_x_at	eukaryotic translation initiation factor 2-alpha kinase 2	901	2.59	0.00000	1.89	0.00000	2.55	0.00000	2.2	0.00002	2.03	0.00002	1.99	0.00001	2.44	0.00000
EIF3EIP	217719_at	eukaryotic translation initiation factor 3, subunit E interacting protein	16353	-2.02	0.00000	-1.59	0.00000	-2.02	0.00000	-1.76	0.00000	-1.6	0.00000	-1.63	0.00000	-1.74	0.00000
EIF4B	211938_at	eukaryotic translation initiation factor 4B	8307	-1.88	0.00000	-1.37	0.00026	-1.77	0.00000	-1.43	0.00001	-1.43	0.00001	-1.53	0.00000	-1.55	0.00000
EIF4B	219599_at	eukaryotic translation initiation factor 4B	1955	-1.58	0.00008	-1.3	0.00293	-1.42	0.00018	-1.46	0.00074	-1.25	0.02002	-1.4	0.00245	-1.7	0.00000
EIF4B	211937_at	eukaryotic translation initiation factor 4B	7116	-1.97	0.00000	-1.48	0.00000	-1.96	0.00000	-1.6	0.00009	-1.54	0.00000	-1.85	0.00000	-1.82	0.00000
EIF4EBP1	221539_at	eukaryotic translation initiation factor 4E binding protein 1	997	-1.21	0.02424	-1.51	0.00022	-1.33	0.00191	-1.26	0.00361	-1.29	0.00105	-1.23	0.00859	-1.22	0.01284
EIF4H	206621_s_at	eukaryotic translation initiation factor 4H	7326	-1.15	0.04048	-1.11	0.01691	-1.16	0.01007	-1.31	0.00199	-1.37	0.00012	-1.31	0.00075	-1.54	0.00001
EMR2	207610_s_at	egf-like module containing, mucin-like, hormone receptor-like 2	1134	1.1	0.10056	-1.5	0.00002	-1.14	0.01079	-1.38	0.00179	-1.24	0.02291	-1.18	0.08326	-1.23	0.01023
EMR3	210724_at	egf-like module containing, mucin-like, hormone receptor-like 3	369	-1.17	0.09383	-1.65#	0.00303	-1.95#	0.00001	-2.42#	0.00009	-1.98#	0.00002	-2.51#	0.00000	-1.90#	0.00031
EPB41L3	206710_s_at	erythrocyte membrane protein band 4.1-like 3	753	1.63	0.00014	1.05	0.43380	1.38	0.00065	1.15	0.27164	1.01	0.89993	1.1	0.42522	1.28	0.03095
EPHB2	209589_s_at	EPH receptor B2	119	3.25*	0.00000	ND		3.46*	0.00000	ND		ND		ND		ND	
ERGIC3	216032_s_at	ERGIC and golgi 3	4084	-1.46	0.00000	-1.36	0.00000	-1.5	0.00000	-1.34	0.00000	-1.38	0.00000	-1.36	0.00000	-1.53	0.00000
ETV7	221680_s_at	ets variant gene 7 (TEL2 oncogene)	422	1.55	0.00000	1.33	0.00026	1.55	0.00000	1.29	0.00300	1.27	0.00746	1.42	0.00024	1.28	0.00001
EVI5	209717_at	ecotropic viral integration site 5	303	-1.29#	0.00646	-1.43#	0.00300	-1.35#	0.00096	-1.44#	0.00065	-1.47#	0.00020	-1.28#	0.00423	-1.75#	0.00003
FAM46A	221766_s_at	family with sequence similarity 46, member A	3034	1.6	0.00000	1.21	0.00621	1.3	0.00335	1.09	0.17658	1.1	0.32120	1.12	0.10782	1.16	0.08447
FAM70A	219895_at	family with sequence similarity 70, member A	78	3.57*	0.00000	1.74*	0.00000	2.64*	0.00000	1.56*	0.01254	ND		1.78*	0.00126	1.67*	0.01016
FANCL	218397_at	Fanconi anemia, complementation group L	264	1.63	0.00001	1.39	0.00056	1.45	0.00108	1.25	0.02494	1.19	0.03011	1.32	0.00191	1.23	0.01400
FBL	211623_s_at	fibrillarin	8706	-1.74	0.00000	-1.37	0.00000	-1.6	0.00000	-1.37	0.00000	-1.34	0.00000	-1.34	0.00000	-1.44	0.00000
FCER1A	211734_s_at	Fc fragment of IgE, high affinity I, receptor for; alpha polypeptide	2024	-1.46	0.00277	-1.69	0.00016	-2.13	0.00000	-2.19	0.00001	-2.4	0.00000	-3.01	0.00000	-2.78	0.00000
FCGR1A	216950_s_at	Fc fragment of IgG, high affinity Ia, receptor (CD64)	1770	1.54	0.00001	1.14	0.30537	1.5	0.00096	1.1	0.46389	-1.09	0.23476	1.06	0.53759	1.07	0.35360
FCGR1B	214511_x_at	Fc fragment of IgG, high affinity Ib, receptor (CD64)	1704	1.69	0.00000	1.11	0.48728	1.53	0.00190	1.2	0.21883	1.08	0.43579	1.24	0.10048	1.21	0.02724
FCGR3A /// FCGR3B	204006_s_at	Fc fragment of IgG, low affinity IIIa, receptor (CD16a) /// Fc fragment of IgG, low affinity IIIb, receptor (CD16b)	7216	1.44	0.00013	-1.32	0.00078	1.04	0.61541	-1.34	0.00598	-1.45	0.00096	-1.46	0.00675	-1.51	0.00056
FCGRT	218831_s_at	Fc fragment of IgG, receptor, transporter, alpha	3650	-1.2	0.01467	-1.32	0.00115	-1.38	0.00005	-1.33	0.00013	-1.63	0.00000	-1.44	0.00028	-1.45	0.00021
FFAR2	221345_at	free fatty acid receptor 2	546	2.68	0.00004	1.29	0.23929	2.11	0.00106	1.24	0.49632	1.13	0.56929	1.06	0.80657	1.24	0.33917
FGL1	205305_at	fibrinogen-like 1	285	-1.12	0.18950	-1.04	0.62991	-1.08	0.33694	1.16	0.19839	1.1	0.28414	1.5	0.00007	1.25	0.03465
FKBP8	40850_at	FK506 binding protein 8, 38kDa	444	ND		ND		ND		ND		ND		ND		3.47*	0.00053
FLJ11151	218610_s_at	hypothetical protein FLJ11151	733	-1.18	0.02682	-1.32	0.03634	-1.43	0.00050	-1.44	0.00004	-1.45	0.00031	-1.55	0.00003	-1.43	0.00006
FLJ11286	218429_s_at	hypothetical protein FLJ11286	3098	1.78	0.00000	1.53	0.00000	1.77	0.00000	1.61	0.00001	1.51	0.00000	1.58	0.00000	1.6	0.00000
FLJ11286	53720_at	hypothetical protein FLJ11286	1603	1.71	0.00000	1.49	0.00000	1.69	0.00000	1.44	0.00001	1.49	0.00000	1.49	0.00000	1.49	0.00000
FLJ20035	218986_s_at	hypothetical protein FLJ20035	1446	3.75	0.00000	2.96	0.00000	3.55	0.00000	2.67	0.00002	2.46	0.00000	2.61	0.00000	2.66	0.00000
FOS	209189_at	v-fos FBJ murine osteosarcoma viral oncogene homolog	18955	-1.4	0.00094	-1.02	0.91257	-1.49	0.00285	-1.67	0.00837	-1.53	0.00282	-1.62	0.00085	-1.52	0.00331
FPRL1	210773_s_at	formyl peptide receptor-like 1	736	1.57	0.00034	1.11	0.28332	1.54	0.00032	1.11	0.19963	1.09	0.14171	1.05	0.65384	-1.07	0.45145
FZD2	210220_at	frizzled homolog 2 (Drosophila)	332	1.02	0.79654	-1.22#	0.14621	-1.05	0.72692	-1.12	0.31604	-1.52#	0.00037	-1.12#	0.24087	-1.64#	0.00288
GAB2	203853_s_at	GRB2-associated binding protein 2	1285	-1.08	0.35801	-1.46#	0.00027	-1.42#	0.00026	-1.63#	0.00048	-1.77#	0.00001	-1.71#	0.00017	-1.80#	0.00001
GAS7	202192_s_at	growth arrest-specific 7	2980	-1.55	0.00000	-1.47	0.00000	-1.62	0.00000	-1.45	0.00000	-1.43	0.00000	-1.34	0.00002	-1.4	0.00000
GAS7	202191_s_at	growth arrest-specific 7	1784	-1.48	0.00004	-1.33	0.00030	-1.51	0.00004	-1.66	0.00002	-1.47	0.00000	-1.64	0.00004	-1.43	0.00068
GATA2	209710_at	GATA binding protein 2	563	-1.12	0.13536	-1.27	0.02946	-1.19	0.17124	-1.3	0.08284	-1.89	0.00018	-1.76	0.00296	-1.74	0.00773
GATM	203178_at	glycine amidinotransferase (L-arginine:glycine amidinotransferase)	129	-1.61	0.00086	-1.37	0.04764	-1.44	0.00339	-1.12	0.35500	-1.26	0.04576	-1.26	0.03167	-1.65	0.00023
GBP1	202270_at	guanylate binding protein 1, interferon-inducible, 67kDa	929	2.32	0.00000	1.33	0.00787	2.13	0.00000	1.37	0.00975	1.19	0.18167	1.34	0.01591	1.26	0.05329
GBP1	202269_x_at	guanylate binding protein 1, interferon-inducible, 67kDa	2762	1.8	0.00000	1.17	0.00425	1.73	0.00000	1.26	0.00026	1.21	0.03786	1.11	0.23141	1.23	0.01037
GCA	203765_at	grancalcin, EF-hand calcium binding protein	2367	-1.12	0.18707	-1.27	0.02101	-1.3	0.00766	-1.34	0.00095	-1.56	0.00000	-1.32	0.00263	-1.59	0.00002
GIMAP4	219243_at	GTPase, IMAP family member 4	6893	1.53	0.00040	1.04	0.79506	1.29	0.06113	1.43	0.00331	1.03	0.73730	1.12	0.34266	-1.04	0.80989
GIT1	218030_at	G protein-coupled receptor kinase interactor 1	940	1.04	0.70275	1.11	0.32720	1.3	0.00428	1.53	0.07368	1.64	0.00063	1.67	0.00498	1.96	0.00042
GLS	203159_at	glutaminase	2115	-1.51	0.00000	-1.19	0.03761	-1.41	0.00022	1.02	0.83490	-1.09	0.15077	-1.11	0.15643	-1.28	0.00901
GLUL	200648_s_at	glutamate-ammonia ligase (glutamine synthetase)	1147	1.56	0.00005	-1.02	0.91021	1.35	0.05554	1.19	0.35193	1.25	0.16702	1.2	0.23483	1.8	0.05642
GMPR	204187_at	guanosine monophosphate reductase	480	1.86*	0.00000	1.27*	0.04478	2.14*	0.00000	2.36*	0.05103	2.43*	0.00161	2.76*	0.03489	4.48*	0.00448
GNG11	204115_at	guanine nucleotide binding protein (G protein), gamma 11	1677	1.37	0.03951	-1.31	0.01728	1.35	0.06739	-1.56	0.00081	-1.32	0.01298	-1.2	0.15963	1.06	0.63679
GPR109B	205220_at	G protein-coupled receptor 109B	1859	1.27	0.31025	1.23	0.49550	-1.03	0.90008	-1	0.99470	-1.37	0.13537	-2.73	0.00030	-1.79	0.02396
GPR18	210279_at	G protein-coupled receptor 18	1618	1.33	0.00333	1.45	0.00541	1.36	0.01453	1.5	0.00082	1.43	0.00285	1.15	0.10211	1.05	0.59431

			Day 1	Day 3		Day 6		Day 10		Day 13		Day 27		Day 42		Day 70	
**Gene Symbol**	**Probe Set ID**	**Gene Title**	**mean signal**	**Fold Chg**	**p-value**	**Fold Chg**	**p-value**	**Fold Chg**	**p-value**	**Fold Chg**	**p-value**	**Fold Chg**	**p-value**	**Fold Chg**	**p-value**	**Fold Chg**	**p-value**
GRN	216041_x_at	granulin	10638	1.5	0.00000	1.06	0.23158	1.3	0.00094	1.2	0.05348	1	0.99417	1.13	0.28612	1.15	0.13857
GSPT1	215438_x_at	G1 to S phase transition 1	1400	-1.13	0.12739	-1.05	0.37751	1.18	0.08714	1.65	0.09908	1.92	0.00063	2.18	0.01772	2.63	0.01075
GYPA	211820_x_at	glycophorin A (MNS blood group)	93	ND		ND		ND		ND		ND		ND		6.86*	0.00046
GYPA	211821_x_at	glycophorin A (MNS blood group)	54	ND		ND		ND		ND		14.69*	0.00141	ND		17.33*	0.00020
GYPB	214407_x_at	glycophorin B (MNS blood group)	244	ND		ND		ND		ND		6.04*	0.00355	6.47*	0.00856	7.52*	0.00022
GYPB	207459_x_at	glycophorin B (MNS blood group)	299	ND		ND		ND		ND		ND		ND		5.02*	0.00003
GYPB /// GYPE	216833_x_at	glycophorin B (MNS blood group) /// glycophorin E	235	ND		ND		ND		ND		3.15*	0.01193	3.29*	0.00824	3.67*	0.00013
GZMB	210164_at	granzyme B (granzyme 2, cytotoxic T-lymphocyte-associated serine esterase 1)	6882	1.61	0.00000	1.28	0.00004	1.48	0.00110	1.53	0.00056	1.33	0.00117	1.37	0.00220	1.51	0.00010
H1F0	208886_at	H1 histone family, member 0	651	3.13	0.00001	1.62	0.00000	2.14	0.00000	1.78	0.00010	1.89	0.00001	2.11	0.00003	2.54	0.00001
H2BFS	208579_x_at	H2B histone family, member S	1578	1.94	0.00003	1.4	0.00813	1.93	0.00018	1.19	0.21376	1.26	0.09250	1.13	0.33230	1.73	0.00106
H3F3B	211998_at	H3 histone, family 3B (H3.3B)	5179	1.1	0.29117	1.01	0.90243	1.07	0.56375	-1.17	0.15029	1.28	0.00093	1.24	0.01746	1.66	0.00005
HADHA	208631_s_at	hydroxyacyl-Coenzyme A dehydrogenase/3-ketoacyl-Coenzyme A thiolase/enoyl-Coenzyme A hydratase (trifunctional protein), alpha subunit	3917	-1.51	0.00000	-1.49	0.00001	-1.62	0.00000	-1.43	0.00002	-1.55	0.00000	-1.54	0.00000	-1.69	0.00000
HBA1 /// HBA2	211699_x_at	hemoglobin, alpha 1 /// hemoglobin, alpha 2	57541	1.09	0.38622	-1.09	0.39088	1.19	0.10586	1.26	0.06477	1.49	0.00009	1.33	0.00472	1.52	0.00001
HBD	206834_at	hemoglobin, delta	2510	-1.12	0.65336	-1	0.98347	2.78	0.00888	4.78	0.02411	7.61	0.00015	6.98	0.00251	9.39	0.00001
HBG1 /// HBG2	213515_x_at	hemoglobin, gamma A /// hemoglobin, gamma G	6275	-1.19	0.44359	-1.19	0.37492	1.62	0.02309	2.45	0.08160	4.67	0.00014	4.72	0.00088	6.53	0.00000
HBG1 /// HBG2	204848_x_at	hemoglobin, gamma A /// hemoglobin, gamma G	6641	-1.2	0.51621	-1.28	0.41289	1.76	0.02209	2.47	0.09180	4.9	0.00001	4.64	0.00093	6.45	0.00000
HBG2	204419_x_at	hemoglobin, gamma G	7115	-1.21	0.48469	-1.26	0.44314	1.79	0.01860	2.49	0.08914	5.04	0.00002	5.02	0.00079	6.75	0.00000
hCG_1776259	206548_at	hypothetical protein FLJ23556	166	1.91*	0.00050	ND		ND		ND		ND		ND		ND	
HEMK1	218621_at	HemK methyltransferase family member 1	382	1.10*	0.30026	1.23*	0.02160	1.27*	0.01584	1.22*	0.10372	1.53*	0.00006	1.54*	0.00184	1.51*	0.00019
HERC5	219863_at	hect domain and RLD 5	1046	4.54	0.00000	2.14	0.00000	4.07	0.00000	2.46	0.00000	2.55	0.00000	2.52	0.00000	2.72	0.00000
HERC6	219352_at	hect domain and RLD 6	651	5.88	0.00000	4.09	0.00000	5.74	0.00000	4.07	0.00000	4.07	0.00000	4.22	0.00000	4.23	0.00000
HESX1	211267_at	HESX homeobox 1	34	11.52*	0.00000	ND		10.31*	0.00000	ND		ND		ND		ND	
HIST1H1C	209398_at	histone cluster 1, H1c	712	2.56	0.00000	1.63	0.00014	2.4	0.00000	1.73	0.00000	2.63	0.00047	2.39	0.00035	3.18	0.00000
HIST1H2AC	215071_s_at	histone cluster 1, H2ac	3816	1.51	0.00142	1.08	0.28337	1.6	0.00091	1.01	0.92044	1.13	0.20578	1.19	0.14911	1.71	0.00081
HIST1H2AE	214469_at	histone cluster 1, H2ae	153	2.40*	0.00002	1.95*	0.00002	3.30*	0.00001	2.12*	0.00023	2.53*	0.00006	2.60*	0.00003	3.84*	0.00000
HIST1H2AG	207156_at	histone cluster 1, H2ag	203	ND		ND		1.84*	0.00161	ND		ND		1.26*	0.10246	1.84*	0.00003
HIST1H2AJ	208583_x_at	histone cluster 1, H2aj	577	1.77*	0.00004	ND		ND		ND		ND		ND		ND	
HIST1H2BC /// HIST1H2BG	214455_at	histone cluster 1, H2bg /// histone cluster 1, H2bc	132	2.52*	0.00011	1.96*	0.00091	2.73*	0.00000	1.91*	0.00013	2.03*	0.00003	2.16*	0.00229	2.82*	0.00000
HIST1H2BD	222067_x_at	histone cluster 1, H2bd	1191	3.37	0.00000	2.51	0.00001	3.42	0.00000	2.04	0.00108	2.42	0.00003	2.02	0.00349	3.28	0.00000
HIST1H2BD	209911_x_at	histone cluster 1, H2bd	1283	2.14	0.00000	1.72	0.00001	2.26	0.00001	1.58	0.00007	1.69	0.00000	1.61	0.00041	2.2	0.00000
HIST1H2BE	208527_x_at	histone cluster 1, H2be	711	1.9	0.00002	1.54	0.00020	2.04	0.00002	1.41	0.00674	1.56	0.00104	1.4	0.00624	2	0.00000
HIST1H2BF	208490_x_at	histone cluster 1, H2bf	731	2.4	0.00001	1.92	0.00001	2.54	0.00001	1.75	0.00025	1.91	0.00003	1.5	0.01006	2.19	0.00000
HIST1H2BG	210387_at	histone cluster 1, H2bg	165	4.25*	0.00002	2.76*	0.00012	3.62*	0.00010	3.47*	0.00002	3.29*	0.00000	2.43*	0.00534	3.37*	0.00000
HIST1H2BG	215779_s_at	histone cluster 1, H2bg	231	3.13*	0.00000	2.23*	0.00001	2.65*	0.00002	1.96*	0.00000	2.18*	0.00000	ND		2.19*	0.00001
HIST1H2BH	208546_x_at	histone cluster 1, H2bh	361	2.10*	0.00016	1.58*	0.00266	2.50*	0.00001	1.43*	0.07162	1.70*	0.00590	1.38*	0.09242	2.45*	0.00026
HIST1H2BI	208523_x_at	histone cluster 1, H2bi	345	2.35	0.00001	1.93	0.00003	2.52	0.00004	1.86	0.00050	1.95	0.00002	1.47	0.02142	2.19	0.00002
HIST1H3D	214472_at	histone cluster 1, H3d	116	3.11*	0.00000	2.76*	0.00000	3.53*	0.00000	ND		ND		ND		ND	
HIST1H3H	206110_at	histone cluster 1, H3h	1317	1.54	0.00224	1.47	0.00406	1.95	0.00021	1.39	0.02999	1.79	0.00009	1.93	0.00045	2.82	0.00000
HIST1H4H	208180_s_at	histone cluster 1, H4h	190	1.63	0.00126	1.85	0.00255	3.12	0.00004	2.18	0.00084	3.04	0.00111	2.42	0.00001	4.65	0.00013
HIST2H2AA3 /// HIST2H2AA4	214290_s_at	histone cluster 2, H2aa3 /// histone cluster 2, H2aa4	7248	3.14	0.00000	1.76	0.00004	2.83	0.00000	1.79	0.00059	2.09	0.00000	2.08	0.00006	2.76	0.00000
HIST2H2AA3 /// HIST2H2AA4	218280_x_at	histone cluster 2, H2aa3 /// histone cluster 2, H2aa4	2660	2.89	0.00000	1.56	0.00089	2.55	0.00001	1.5	0.01173	1.86	0.00002	1.87	0.00013	2.45	0.00000
HIST2H2BE	202708_s_at	histone cluster 2, H2be	1609	2.62	0.00000	1.63	0.00008	2.47	0.00000	1.47	0.00770	1.75	0.00019	1.68	0.00141	2.28	0.00000
HIST3H2A	221582_at	histone cluster 3, H2a	953	1.11	0.22967	1.12	0.24862	1.21	0.05131	1.33	0.00969	1.44	0.00050	1.53	0.00000	1.29	0.00226
HK3	205936_s_at	hexokinase 3 (white cell)	2708	-1.08	0.19525	-1.45	0.00039	-1.28	0.03063	-1.37	0.00345	-1.64	0.00007	-1.32	0.02022	-1.45	0.00026
HLA-DMB	203932_at	major histocompatibility complex, class II, DM beta	9491	-1.23	0.04403	-1.34	0.02458	-1.49	0.00797	-1.4	0.01691	-1.88	0.00006	-1.87	0.00001	-2.01	0.00000
HLA-DQB1	209823_x_at	major histocompatibility complex, class II, DQ beta 1	5133	-1.45	0.00005	-1.4	0.00142	-1.59	0.00005	-1.26	0.03170	-1.42	0.00018	-1.41	0.00372	-1.48	0.00756
HMBS	203040_s_at	hydroxymethylbilane synthase	939	-1.05	0.26165	-1.05	0.30238	-1.01	0.79700	1.26	0.06478	1.4	0.00116	1.47	0.00136	1.5	0.00020
HNRNPA1 /// LOC728844	222040_at	heterogeneous nuclear ribonucleoprotein A1 /// hypothetical protein LOC728844	1693	-1.07	0.51167	1.2	0.17571	1.09	0.52122	-1.04	0.80102	1.3	0.03336	1.19	0.06884	1.62	0.00099
HPSE	219403_s_at	heparanase	1257	1.7	0.00000	1.2	0.00428	1.57	0.00000	1.29	0.01857	1.21	0.03036	1.32	0.02143	1.55	0.00005
HRASLS	219983_at	HRAS-like suppressor	172	-1.33#	0.00621	-1.48#	0.00168	-1.28#	0.00793	-1.52#	0.00053	-1.22#	0.02570	-1.20#	0.04041	-1.23#	0.07581
ICAM3	204949_at	intercellular adhesion molecule 3	5713	-1.28	0.00000	-1.32	0.00031	-1.43	0.00000	-1.41	0.00007	-1.56	0.00002	-1.37	0.00026	-1.56	0.00024
IFI16	206332_s_at	interferon, gamma-inducible protein 16	4915	1.78	0.00000	1.51	0.00006	1.74	0.00000	1.47	0.00007	1.44	0.00006	1.52	0.00000	1.48	0.00001
IFI16	208966_x_at	interferon, gamma-inducible protein 16	7072	1.76	0.00000	1.45	0.00001	1.68	0.00000	1.51	0.00008	1.45	0.00044	1.57	0.00001	1.4	0.00008
IFI16	208965_s_at	interferon, gamma-inducible protein 16	2462	1.74	0.00000	1.45	0.00001	1.76	0.00000	1.37	0.00758	1.34	0.01364	1.22	0.01812	1.28	0.00340
IFI27	202411_at	interferon, alpha-inducible protein 27	322	73.30*	0.00000	70.29*	0.00000	98.01*	0.00000	93.53*	0.00000	94.83*	0.00000	97.44*	0.00000	107.70*	0.00000
IFI35	209417_s_at	interferon-induced protein 35	1906	3.19	0.00000	2.03	0.00000	2.86	0.00000	2.02	0.00000	1.76	0.00011	1.88	0.00013	1.88	0.00000
IFI44	214453_s_at	interferon-induced protein 44	2711	5.97	0.00000	4.61	0.00000	6	0.00000	4.65	0.00000	4.91	0.00000	5.2	0.00000	5.45	0.00000
IFI44	214059_at	Interferon-induced protein 44	523	7.09	0.00000	4.75	0.00000	6.45	0.00000	4.78	0.00000	4.53	0.00000	5.15	0.00000	4.98	0.00000

			Day 1	Day 3		Day 6		Day 10		Day 13		Day 27		Day 42		Day 70	
**Gene Symbol**	**Probe Set ID**	**Gene Title**	**mean signal**	**Fold Chg**	**p-value**	**Fold Chg**	**p-value**	**Fold Chg**	**p-value**	**Fold Chg**	**p-value**	**Fold Chg**	**p-value**	**Fold Chg**	**p-value**	**Fold Chg**	**p-value**
IFI44L	204439_at	interferon-induced protein 44-like	1836	10.07	0.00000	8.17	0.00000	9.68	0.00000	7.58	0.00000	7.5	0.00000	7.99	0.00000	8.38	0.00000
IFIH1	219209_at	interferon induced with helicase C domain 1	616	3.31	0.00000	2.01	0.00000	3.26	0.00000	2.37	0.00000	2.12	0.00000	2.29	0.00000	2.15	0.00000
IFIT1	203153_at	interferon-induced protein with tetratricopeptide repeats 1	828	12.23*	0.00000	4.06*	0.00000	9.91*	0.00000	6.00*	0.00000	4.76*	0.00004	5.59*	0.00000	4.81*	0.00006
IFIT2	217502_at	interferon-induced protein with tetratricopeptide repeats 2	929	4.16*	0.00001	ND		3.73*	0.00018	1.71*	0.00064	ND		ND		1.40*	0.02177
IFIT3	204747_at	interferon-induced protein with tetratricopeptide repeats 3	1316	7.11	0.00000	3.3	0.00000	6.76	0.00000	3.79	0.00000	3.43	0.00001	3.54	0.00001	3.85	0.00000
IFIT5	203596_s_at	interferon-induced protein with tetratricopeptide repeats 5	677	2.74*	0.00000	1.86*	0.00015	2.72*	0.00000	2.36*	0.00000	2.03*	0.00001	2.24*	0.00000	1.80*	0.00028
IFIT5	203595_s_at	interferon-induced protein with tetratricopeptide repeats 5	965	2.25	0.00000	1.41	0.00015	1.99	0.00001	1.56	0.00002	1.45	0.00054	1.52	0.00001	1.3	0.02059
IFITM1	201601_x_at	interferon induced transmembrane protein 1 (9-27)	18622	2.01	0.00000	1.63	0.00000	1.89	0.00000	1.67	0.00000	1.67	0.00000	1.74	0.00000	1.74	0.00000
IFITM1	214022_s_at	interferon induced transmembrane protein 1 (9-27)	22532	1.71	0.00000	1.47	0.00000	1.68	0.00000	1.54	0.00000	1.54	0.00000	1.55	0.00000	1.58	0.00000
IFITM2	201315_x_at	interferon induced transmembrane protein 2 (1-8D)	22495	1.7	0.00000	1.47	0.00000	1.64	0.00000	1.55	0.00000	1.36	0.00001	1.43	0.00000	1.5	0.00000
IFITM3	212203_x_at	interferon induced transmembrane protein 3 (1-8U)	15734	2.15	0.00000	1.77	0.00000	2.07	0.00000	1.75	0.00000	1.67	0.00000	1.78	0.00000	1.97	0.00000
IFT81	219372_at	intraflagellar transport 81 homolog (Chlamydomonas)	86	-1.51#	0.00063	-1.11#	0.28166	-1.18#	0.26463	-1.28#	0.03452	1.08#	0.62846	-1.09#	0.46919	-1.32#	0.02870
IGF2BP3	203820_s_at	insulin-like growth factor 2 mRNA binding protein 3	176	1.67	0.00002	1.17	0.02011	1.66	0.00009	1.12	0.16337	-1.05	0.61512	1.03	0.82312	1.14	0.19708
IGHA1	215118_s_at	Immunoglobulin heavy constant alpha 1	1117	-1.84	0.00000	-1.47	0.00016	-1.97	0.00000	-1.34	0.02670	-1.5	0.00002	-1.47	0.06343	-2.01	0.00270
IGHD	214973_x_at	immunoglobulin heavy constant delta	886	-1.3	0.00482	-1.08	0.40400	-1.5	0.00014	-1.14	0.22305	-1.29	0.01542	-1.35	0.00568	-1.56#	0.00112
IGHG1 /// IGHG2 /// IGHG3 /// IGHM /// IGHV4-31	211430_s_at	immunoglobulin heavy constant gamma 1 (G1m marker) /// gamma 2 (G2m marker) /// gamma 3 (G3m marker) /// mu /// immunoglobulin heavy variable 4-31	6712	-1.39	0.00672	-1.09	0.55640	-2.17	0.00047	-1.64	0.02145	-2.3	0.00077	-1.83	0.02203	-3.17	0.00027
IGHV1-69	211635_x_at	Immunoglobulin heavy variable 1-69	944	-1.44	0.00149	-1.11	0.31515	-1.47	0.00335	-1.34	0.05517	-1.24	0.07239	-1.62	0.00035	-1.59	0.00875
IL13RA1	201887_at	interleukin 13 receptor, alpha 1	1657	-1.53	0.00005	-1.4	0.00009	-1.57	0.00002	-1.44	0.00019	-1.46	0.00001	-1.4	0.00009	-1.47	0.00004
IL1RN	212659_s_at	interleukin 1 receptor antagonist	700	1.51	0.00000	1.12	0.01432	1.39	0.00002	1.12	0.16678	ND		ND		ND	
IL1RN	216243_s_at	interleukin 1 receptor antagonist	982	1.56	0.00000	1.11	0.17858	1.51	0.00005	1.27	0.00595	1.12#	0.21890	1.13	0.34278	1.3	0.02978
IL1RN	212657_s_at	interleukin 1 receptor antagonist	1454	2.12	0.00001	1.14	0.11428	1.56	0.00018	1.36	0.11997	1.19	0.20644	1.18	0.10854	1.26	0.01422
IL8	217281_x_at	Interleukin 8	412	-1.48#	0.00728	-1.17#	0.22690	-1.95#	0.00068	-1.36#	0.16970	-1.30#	0.09618	-1.92#	0.00302	-1.79#	0.01762
INSR	213792_s_at	insulin receptor	364	-1.48	0.00320	-1.56#	0.00091	-1.61#	0.00016	-1.94	0.00002	-1.96#	0.00002	-1.97#	0.00000	-1.60#	0.00049
IRF1	202531_at	interferon regulatory factor 1	4893	-1.13	0.11828	-1.36	0.00079	-1.24	0.00128	-1.33	0.00200	-1.62	0.00001	-1.6	0.00003	-1.59	0.00000
IRF7	208436_s_at	interferon regulatory factor 7	3052	3.65	0.00000	2.44	0.00000	3.34	0.00000	2.48	0.00000	2.22	0.00000	2.49	0.00000	2.38	0.00000
IRS2	209185_s_at	insulin receptor substrate 2	7235	-1.69	0.00319	-1.36	0.04881	-1.57	0.00380	-1.74	0.00001	-1.4	0.00297	-1.53	0.00113	-1.32	0.05489
IRS2	209184_s_at	insulin receptor substrate 2	3142	-1.54	0.00254	-1.22	0.12321	-1.38	0.01327	-1.5	0.00000	-1.21	0.02529	-1.41	0.00154	-1.35	0.02744
ISG15	205483_s_at	ISG15 ubiquitin-like modifier	4167	6.76	0.00000	3.76	0.00000	6.59	0.00000	4	0.00000	3.9	0.00000	4.36	0.00000	4.23	0.00000
ISG20	204698_at	interferon stimulated exonuclease gene 20kDa	2731	2.54	0.00000	1.82	0.00000	2.64	0.00000	1.77	0.00000	1.71	0.00002	1.64	0.00002	1.68	0.00000
ISG20	33304_at	interferon stimulated exonuclease gene 20kDa	5053	1.79	0.00000	1.49	0.00000	1.86	0.00000	1.51	0.00000	1.47	0.00000	1.44	0.00000	1.44	0.00000
ITGA2B	206493_at	integrin, alpha 2b (platelet glycoprotein IIb of IIb/IIIa complex, antigen CD41)	1644	1.44	0.00581	-1.58	0.01623	1.17	0.24084	-1.79	0.00486	-1.67	0.00097	-1.45	0.01784	-1.19	0.14249
ITGAE	205055_at	integrin, alpha E (antigen CD103, human mucosal lymphocyte antigen 1; alpha polypeptide)	3873	-1.57	0.00000	-1.47	0.00001	-1.59	0.00000	-1.43	0.00000	-1.54	0.00000	-1.47	0.00000	-1.71	0.00000
JAK2	205842_s_at	Janus kinase 2 (a protein tyrosine kinase)	1457	1.64	0.00000	1.16	0.00618	1.42	0.00000	1.15	0.13422	1.08	0.42341	1.03	0.74974	1.12	0.22717
JAK2	205841_at	Janus kinase 2 (a protein tyrosine kinase)	326	1.65	0.00004	1.16	0.14528	1.25	0.01794	-1.29	0.05805	-1.07	0.42345	-1.01	0.96680	-1.16	0.14795
JUP	201015_s_at	junction plakoglobin	549	3.39*	0.00000	1.86*	0.00046	2.93*	0.00000	2.51*	0.00019	1.78*	0.00188	1.87*	0.00646	2.03*	0.00139
KCTD14	58916_at	potassium channel tetramerisation domain containing 14	151	1.82*	0.00002	1.63*	0.00002	2.24*	0.00001	1.50*	0.00208	ND		1.34*	0.02279	1.31*	0.00084
KIAA0125	206478_at	KIAA0125	947	-1.57	0.00000	-1.14	0.17871	-1.37	0.00081	-1.18	0.20607	-1.02	0.87180	-1.05	0.65459	-1.13	0.24014
KIAA0430	202386_s_at	KIAA0430	2757	1.07	0.12730	1.16	0.00395	1.19	0.00030	1.39	0.00039	1.42	0.00001	1.5	0.00235	1.6	0.00076
KIAA1466	222139_at	KIAA1466 gene	430	1.64	0.00002	1.46	0.01651	1.56	0.00192	1.05	0.62352	1.33	0.01236	1.29	0.01021	1.45	0.00221
KIR2DL4	208426_x_at	killer cell immunoglobulin-like receptor, two domains, long cytoplasmic tail, 4	606	1.19	0.02849	1.12	0.17349	1.18	0.04725	1.14	0.10311	1.27	0.00654	1.16	0.04997	1.52	0.00003
KLF4	221841_s_at	Kruppel-like factor 4 (gut)	7570	-1.04	0.67717	-1.43	0.00123	-1.23	0.03220	-1.58	0.00100	-1.48	0.00016	-1.55	0.00028	-1.23	0.01153
KLF4	220266_s_at	Kruppel-like factor 4 (gut)	1812	-1.1	0.30637	-1.26	0.02746	-1.21	0.06797	-1.49	0.00716	-1.64	0.00001	-1.69	0.00083	-1.33	0.01373
KLF9	203542_s_at	Kruppel-like factor 9	1371	-1.39	0.03089	-1.05	0.79164	-1.1	0.53720	-1.57	0.00026	-1.11	0.40340	-1.49	0.00491	-1.13	0.39022
KLHL21	203068_at	kelch-like 21 (Drosophila)	746	-1.30#	0.01225	-1.14	0.28866	-1.56#	0.00040	-1.22#	0.06043	-1.47#	0.00373	-1.43#	0.00510	-1.62#	0.00025
KLRB1	214470_at	killer cell lectin-like receptor subfamily B, member 1	9732	-1.41	0.00004	-1.21	0.00155	-1.32	0.00004	-1.25	0.00411	-1.32	0.00001	-1.37	0.00002	-1.5	0.00001
KLRD1	207795_s_at	killer cell lectin-like receptor subfamily D, member 1	2957	1.51	0.00000	1.34	0.00043	1.32	0.00075	1.43	0.00004	1.11	0.23922	1.01	0.90546	1.05	0.35928
KMO	211138_s_at	kynurenine 3-monooxygenase (kynurenine 3-hydroxylase)	791	1.77	0.00000	1.24	0.00395	1.64	0.00000	1.29	0.00005	1.24	0.00379	1.3	0.00605	1.23	0.00291
KMO	205306_x_at	kynurenine 3-monooxygenase (kynurenine 3-hydroxylase)	699	1.73	0.00000	1.23	0.00273	1.57	0.00000	1.33	0.00005	1.27	0.00030	1.26	0.00193	1.22	0.00231
KRT10	207023_x_at	keratin 10 (epidermolytic hyperkeratosis; keratosis palmaris et plantaris)	3120	-1.54	0.00000	-1.3	0.00033	-1.47	0.00000	-1.46	0.00002	-1.31	0.00232	-1.37	0.00004	-1.43	0.00011

			Day 1	Day 3		Day 6		Day 10		Day 13		Day 27		Day 42		Day 70	
Gene Symbol	Probe Set ID	Gene Title	mean signal	Fold Chg	p-value	Fold Chg	p-value	Fold Chg	p-value	Fold Chg	p-value	Fold Chg	p-value	Fold Chg	p-value	Fold Chg	p-value
KRT10	210633_x_at	keratin 10 (epidermolytic hyperkeratosis; keratosis palmaris et plantaris)	4516	-1.63	0.00000	-1.37	0.00042	-1.6	0.00000	-1.53	0.00003	-1.37	0.00073	-1.41	0.00086	-1.45	0.00036
LAG3	206486_at	lymphocyte-activation gene 3	434	1.75*	0.00000	1.47*	0.00022	1.81*	0.00000	ND		ND		ND		ND	
LAMP3	205569_at	lysosomal-associated membrane protein 3	185	6.02*	0.00000	3.11*	0.00000	6.68*	0.00000	3.29*	0.00000	3.80*	0.00000	3.93*	0.00000	4.41*	0.00000
LAP3	217933_s_at	leucine aminopeptidase 3	3873	2.04	0.00000	1.49	0.00001	1.96	0.00000	1.53	0.00017	1.44	0.00035	1.48	0.00258	1.59	0.00000
LBA1	213261_at	lupus brain antigen 1	1508	1.52	0.00000	1.29	0.00006	1.49	0.00000	1.38	0.00003	1.26	0.00349	1.32	0.00000	1.24	0.00008
LCN2	212531_at	lipocalin 2 (oncogene 24p3)	559	1.75*	0.01044	1.32*	0.07925	2.62*	0.00001	1.92*	0.00147	1.83*	0.00028	1.76*	0.00162	2.84*	0.00012
LDLR	202068_s_at	low density lipoprotein receptor (familial hypercholesterolemia)	994	1.7	0.00001	1.39	0.00003	1.5	0.00002	1.33	0.00168	1.35	0.00023	1.35	0.00041	1.37	0.00001
LEPR	209894_at	leptin receptor	154	-1.16	0.09118	-1.38	0.00131	-1.09	0.18777	-1.48	0.00890	-1.57	0.00010	-1.22	0.05404	-1.19	0.15730
LGALS3BP	200923_at	lectin, galactoside-binding, soluble, 3 binding protein	970	3.99*	0.00000	2.23*	0.00000	4.02*	0.00000	2.84*	0.00000	2.61*	0.00005	2.95*	0.00000	3.14*	0.00000
LGALS9	203236_s_at	lectin, galactoside-binding, soluble, 9 (galectin 9)	1832	1.88	0.00000	1.34	0.00037	1.75	0.00000	1.48	0.00001	1.31	0.00145	1.54	0.00006	1.67	0.00001
LILRA1	210660_at	leukocyte immunoglobulin-like receptor, subfamily A (with TM domain), member 1	2204	1.18	0.03025	-1.32	0.00001	-1.06	0.40321	-1.32	0.00301	-1.5	0.00003	-1.28	0.02738	-1.19	0.03794
LILRA2	211102_s_at	leukocyte immunoglobulin-like receptor, subfamily A (with TM domain), member 2	981	1.15	0.03835	-1.1	0.13326	-1.01	0.87071	-1.18	0.03044	-1.54	0.00000	-1.34	0.00681	-1.33	0.00169
LILRA3	206881_s_at	leukocyte immunoglobulin-like receptor, subfamily A (without TM domain), member 3	1824	1.57	0.00001	-1.04	0.72135	1.29	0.01684	1.08	0.44151	-1.08	0.46215	1.18	0.26344	1.27	0.05120
LILRB2	210146_x_at	leukocyte immunoglobulin-like receptor, subfamily B (with TM and ITIM domains), member 2	2758	1.54	0.00000	1.07	0.43348	1.32	0.00268	-1.04	0.66149	-1.08	0.36402	1.04	0.67447	1.08	0.30925
LOC150759	213703_at	hypothetical protein LOC150759	356	-1.04	0.55740	1.53	0.00006	1.26	0.07798	1.16	0.11009	1.31	0.03802	1.43	0.00255	1.39	0.02541
LOC26010	222154_s_at	viral DNA polymerase-transactivated protein 6	857	4.32	0.00000	2.45	0.00000	4.17	0.00000	3.03	0.00000	2.97	0.00000	3.06	0.00000	3.28	0.00000
LOC391020	216565_x_at	interferon induced transmembrane protein pseudogene	2444	2.68	0.00000	1.96	0.00000	2.65	0.00000	2.17	0.00000	2.13	0.00000	2.18	0.00000	2.48	0.00000
LOC442171	217379_at	similar to ribosomal protein L10	9003	-1.65	0.00000	-1.25	0.01718	-1.55	0.00000	-1.44	0.00003	-1.42	0.00036	-1.37	0.00039	-1.52	0.00000
LOC442215	217142_at	similar to Elongation FacTor family member (eft-4)	873	-1.63	0.00017	-1.23	0.12208	-1.59	0.00054	-1.49	0.00588	-1.23	0.01830	-1.47	0.00246	-1.4	0.00225
LST1	214181_x_at	leukocyte specific transcript 1	6884	1.03	0.67475	-1.45	0.00024	-1.27	0.01046	-1.42	0.00112	-1.6	0.00006	-1.39	0.00616	-1.38	0.00072
LST1	215633_x_at	leukocyte specific transcript 1	6680	1.05	0.45336	-1.46	0.00005	-1.25	0.00769	-1.4	0.00203	-1.6	0.00009	-1.39	0.00855	-1.42	0.00083
LTA4H	208771_s_at	leukotriene A4 hydrolase	15548	-1.73	0.00000	-1.74	0.00000	-1.95	0.00000	-1.71	0.00001	-1.55	0.00007	-1.61	0.00000	-1.62	0.00001
LY6E	202145_at	lymphocyte antigen 6 complex, locus E	2143	4.11	0.00000	2.68	0.00000	3.98	0.00000	3.07	0.00000	2.78	0.00000	3.04	0.00000	3.39	0.00000
MAFB	218559_s_at	v-maf musculoaponeurotic fibrosarcoma oncogene homolog B (avian)	4224	1.65	0.00000	1.24	0.04572	1.52	0.00011	1.23	0.24339	1.16	0.06186	1.22	0.09459	1.12	0.21386
MAP3K12	205448_s_at	mitogen-activated protein kinase kinase kinase 12	560	-1.14	0.07723	-1.12	0.05936	-1.08	0.30760	-1.43	0.00011	-1.1	0.40330	-1.28#	0.00146	-1.5	0.00047
MARCKS	201670_s_at	myristoylated alanine-rich protein kinase C substrate	896	2.43	0.00000	1.14	0.20741	1.82	0.00002	1.13	0.42762	1.12	0.39994	-1.05	0.72064	1.26	0.12299
MARCKS	201669_s_at	myristoylated alanine-rich protein kinase C substrate	3083	2.07	0.00000	1.2	0.02656	1.69	0.00000	1.25	0.05666	1.13	0.17988	1.17	0.13138	1.22	0.02941
MARCKS	201668_x_at	myristoylated alanine-rich protein kinase C substrate	412	1.93	0.00069	1.19	0.06105	1.4	0.00035	1.01	0.88069	1.13#	0.21574	1.05#	0.73004	1.13	0.34104
MARCKS	213002_at	myristoylated alanine-rich protein kinase C substrate	836	1.74	0.00000	1.15	0.07049	1.44	0.00073	1.11	0.39621	1.15	0.04558	1.04	0.62333	1.1	0.22370
MARCO	205819_at	macrophage receptor with collagenous structure	140	2.58*	0.00009	ND		ND		ND		ND		ND		ND	
MARK2	203942_s_at	MAP/microtubule affinity-regulating kinase 2	639	-1.09	0.26664	-1.14	0.21105	-1.2	0.03976	-1.1	0.26517	-1.41#	0.00012	-1.50#	0.00050	-1.25	0.01759
MEGF9	212831_at	multiple EGF-like-domains 9	510	-1.37	0.00607	-1.75#	0.00014	-1.45#	0.00879	-1.62#	0.01539	-1.54#	0.00246	-1.72#	0.00006	-1.18#	0.13295
MGC5618	221477_s_at	hypothetical protein MGC5618	2232	-1.27	0.08924	-1.57	0.00735	-1.59	0.00122	-1.67	0.00007	-1.48	0.00960	-1.6	0.00137	-1.69	0.00040
MICAL2	212473_s_at	microtubule associated monoxygenase, calponin and LIM domain containing 2	2623	-1.63	0.00000	-1.44	0.00002	-1.49	0.00000	1	0.98122	-1.01	0.92530	1.01	0.92674	1.15	0.24246
MICAL2	212472_at	microtubule associated monoxygenase, calponin and LIM domain containing 2	1906	-1.68	0.00000	-1.46	0.00049	-1.56	0.00004	-1.07	0.73078	-1.11	0.41955	-1	0.99629	1.13	0.37395
MLC1	213395_at	megalencephalic leukoencephalopathy with subcortical cysts 1	297	ND		ND		ND		1.51*	0.02751	ND		ND		1.74*	0.00022
MMRN1	205612_at	multimerin 1	100	1.22	0.28054	-1.32	0.01295	-1.05	0.74988	-1.32#	0.03212	-1.45#	0.00734	-1.67#	0.00081	-1.50#	0.00877
MRPL42	217919_s_at	mitochondrial ribosomal protein L42	1409	1.53	0.00003	1.27	0.00690	1.34	0.01092	1.3	0.03812	1.16	0.10065	1.12	0.21335	1.14	0.11808
MRPS12	204330_s_at	mitochondrial ribosomal protein S12	148	1.19	0.10127	1.28	0.02929	1.35	0.00857	1.26	0.09073	1.58	0.00060	1.32	0.03246	1.5	0.00630
MS4A4A	219607_s_at	membrane-spanning 4-domains, subfamily A, member 4	588	3.86	0.00000	1.36	0.02743	2.59	0.00000	1.64	0.00163	1.54	0.00429	1.97	0.00178	1.67	0.00009
MT1E	212859_x_at	metallothionein 1E	1847	1.74	0.00000	1.33	0.00042	1.63	0.00001	1.34	0.00846	1.17	0.16731	1.39	0.00576	1.44	0.00001
MT1F	217165_x_at	metallothionein 1F	1263	1.8	0.00000	1.28	0.00067	1.77	0.00000	1.28	0.01099	1.25	0.01476	1.23	0.01319	1.38	0.00037
MT1G	204745_x_at	metallothionein 1G	2358	1.63	0.00000	1.19	0.01953	1.54	0.00001	1.17	0.01654	1.18	0.00071	1.27	0.00210	1.38	0.00000
MT1P2	211456_x_at	metallothionein 1 pseudogene 2	2938	2.38	0.00000	1.32	0.01842	2.01	0.00012	1.38	0.00862	1.22	0.02554	1.35	0.02432	1.71	0.00000
MT1P2 /// MT1A /// MT1M	216336_x_at	metallothionein 1A /// metallothionein 1M /// metallothionein 1 pseudogene 2	1363	1.72	0.00000	1.24	0.00222	1.72	0.00000	1.34	0.00746	1.21	0.02322	1.34	0.00141	1.56	0.00000
MT1P2 /// MT1H	206461_x_at	metallothionein 1H /// metallothionein 1 pseudogene 2	2264	2.28	0.00000	1.45	0.00064	2.12	0.00000	1.46	0.00079	1.34	0.00160	1.49	0.00007	1.66	0.00017
MT1X	208581_x_at	metallothionein 1X	3045	2.49	0.00000	1.53	0.00015	2.24	0.00000	1.4	0.00192	1.25	0.02202	1.36	0.00081	1.67	0.00021

			Day 1	Day 3		Day 6		Day 10		Day 13		Day 27		Day 42		Day 70	
**Gene Symbol**	**Probe Set ID**	**Gene Title**	**mean signal**	**Fold Chg**	**p-value**	**Fold Chg**	**p-value**	**Fold Chg**	**p-value**	**Fold Chg**	**p-value**	**Fold Chg**	**p-value**	**Fold Chg**	**p-value**	**Fold Chg**	**p-value**
MT1X	204326_x_at	metallothionein 1X	1682	1.56	0.00000	1.29	0.00005	1.57	0.00000	1.24	0.00607	1.26	0.00311	1.28	0.00026	1.39	0.00008
MT2A	212185_x_at	metallothionein 2A	8207	2.03	0.00000	1.37	0.00063	1.91	0.00000	1.26	0.00606	1.29	0.00091	1.37	0.00030	1.49	0.00001
MTMR11	205076_s_at	myotubularin related protein 11	1645	-1.06	0.23040	-1.43	0.00010	-1.21	0.03474	-1.43	0.00013	-1.5	0.00001	-1.35	0.00241	-1.42	0.00013
MX1	202086_at	myxovirus (influenza virus) resistance 1, interferon-inducible protein p78 (mouse)	4087	5.02	0.00000	3.52	0.00000	4.88	0.00000	3.81	0.00000	3.74	0.00000	3.93	0.00000	4.13	0.00000
MX2	204994_at	myxovirus (influenza virus) resistance 2 (mouse)	3661	3.13	0.00000	2.1	0.00000	2.92	0.00000	2.21	0.00000	2.23	0.00000	2.34	0.00000	2.43	0.00000
MYCL1	214058_at	v-myc myelocytomatosis viral oncogene homolog 1, lung carcinoma derived (avian)	1261	1.06	0.47987	-1.23	0.00602	-1.07	0.37074	-1.22	0.05582	-1.39	0.00048	-1.29	0.00699	-1.57	0.00001
MYL4	210088_x_at	myosin, light chain 4, alkali; atrial, embryonic	698	-1.01	0.89481	-1.06	0.62400	1.41	0.00428	2.23	0.06526	2.93	0.00301	3.23	0.01850	3.71	0.00070
MYL4	217274_x_at	myosin, light chain 4, alkali; atrial, embryonic	406	1.04	0.63769	1.08#	0.56747	1.37	0.00918	1.86	0.01837	2.52	0.00075	2.48	0.00466	3.18	0.00010
N4BP1	204601_at	Nedd4 binding protein 1	752	1.92*	0.00000	1.46*	0.00044	1.70*	0.00000	1.45*	0.00001	1.27*	0.02422	1.38*	0.00143	1.18*	0.01345
N4BP1	221867_at	Nedd4 binding protein 1	677	1.54	0.00000	1.54	0.00000	1.71	0.00000	1.56	0.00000	1.57	0.00002	1.71	0.00003	1.59	0.00000
N4BP1	32069_at	Nedd4 binding protein 1	889	1.83	0.00000	1.54	0.00000	1.85	0.00000	1.56	0.00001	1.54	0.00001	1.43	0.00007	1.54	0.00000
NDC80	204162_at	NDC80 homolog, kinetochore complex component (S. cerevisiae)	165	1.51	0.00001	1.15	0.15726	1.37	0.00222	1.23#	0.11073	1.22#	0.09451	1.32#	0.02042	1.33#	0.01371
NELL2	203413_at	NEL-like 2 (chicken)	4486	-1.51	0.00002	-1.06	0.17764	-1.41	0.00000	-1.28	0.00440	-1.01	0.92974	-1.14	0.04230	-1.1	0.09855
NGFRAP1	217963_s_at	nerve growth factor receptor (TNFRSF16) associated protein 1	3633	1.67	0.00025	1.14	0.15893	1.71	0.00038	1.31	0.05180	1.64	0.00000	1.61	0.00105	2.26	0.00000
NMI	203964_at	N-myc (and STAT) interactor	3919	1.86	0.00000	1.27	0.00106	1.63	0.00000	1.37	0.00000	1.23	0.01096	1.24	0.00757	1.32	0.00005
NRG1	206343_s_at	neuregulin 1	863	-2.73#	0.00009	-1.70#	0.00761	-2.55#	0.00032	-1.78#	0.00875	-1.82#	0.00082	-1.50#	0.02875	-1.55#	0.04408
NRGN	204081_at	neurogranin (protein kinase C substrate, RC3)	8064	1.3	0.06488	-1.43	0.01201	1.17	0.15988	-1.56	0.00188	-1.62	0.00002	-1.37	0.03281	1	0.96273
OAS1	205552_s_at	2',5'-oligoadenylate synthetase 1, 40/46kDa	1310	4.29	0.00000	2.4	0.00000	3.76	0.00000	2.51	0.00000	2.08	0.00008	2.33	0.00007	2.33	0.00000
OAS1	202869_at	2',5'-oligoadenylate synthetase 1, 40/46kDa	2334	3.91	0.00000	2.04	0.00000	3.51	0.00000	2.37	0.00001	1.98	0.00002	2.65	0.00003	2.3	0.00002
OAS2	204972_at	2'-5'-oligoadenylate synthetase 2, 69/71kDa	2791	3.5	0.00000	2.37	0.00000	3.4	0.00000	2.9	0.00000	2.4	0.00000	2.49	0.00000	2.49	0.00000
OAS2	206553_at	2'-5'-oligoadenylate synthetase 2, 69/71kDa	833	1.93	0.00000	1.49	0.00000	1.99	0.00000	1.57	0.00000	1.6	0.00007	1.51	0.00008	1.57	0.00001
OAS3	218400_at	2'-5'-oligoadenylate synthetase 3, 100kDa	2136	4.48	0.00000	2.64	0.00000	3.76	0.00000	2.74	0.00000	2.34	0.00001	2.45	0.00001	2.83	0.00000
OASL	205660_at	2'-5'-oligoadenylate synthetase-like	954	4.56	0.00000	2.13	0.00000	4.07	0.00000	2.65	0.00000	2.23	0.00000	2.45	0.00000	2.58	0.00000
OASL	210797_s_at	2'-5'-oligoadenylate synthetase-like	1144	4.02	0.00000	2.39	0.00000	3.69	0.00000	2.55	0.00000	2.32	0.00000	2.55	0.00000	2.51	0.00000
OLFM1	213131_at	olfactomedin 1	371	-2.34#	0.00000	-1.44	0.00432	-1.79#	0.00023	-1.18#	0.04273	-1.38#	0.01579	-1.31#	0.00466	-1.23#	0.16443
P2RX7	207091_at	purinergic receptor P2X, ligand-gated ion channel, 7	404	1.05	0.51215	-1.07	0.35499	-1.14	0.07667	-1.22#	0.08093	-1.55#	0.00033	-1.32#	0.01196	-1.38#	0.00094
P2RY13	220005_at	purinergic receptor P2Y, G-protein coupled, 13	1470	1.41	0.03370	-1.25	0.09869	1.08	0.61443	-1.09	0.34985	-1.62	0.00005	-1.31	0.11287	-1.58	0.01795
PABPC4	201064_s_at	poly(A) binding protein, cytoplasmic 4 (inducible form)	3291	-1.53	0.00000	-1.31	0.00008	-1.49	0.00000	-1.41	0.00001	-1.27	0.00024	-1.4	0.00000	-1.28	0.00003
PALLD	200897_s_at	palladin, cytoskeletal associated protein	531	-1.06	0.55671	-1.3	0.00072	-1.34	0.00352	-1.4	0.00134	-1.62	0.00007	-1.47	0.00125	-1.55	0.00007
PAPSS2	203060_s_at	3'-phosphoadenosine 5'-phosphosulfate synthase 2	319	-1.73#	0.00005	-1.74#	0.00001	-1.76#	0.00001	-1.58#	0.00008	-1.55#	0.00005	-1.32#	0.00424	-1.57#	0.00000
PARP11	220315_at	poly (ADP-ribose) polymerase family, member 11	172	1.60*	0.00004	1.24*	0.07717	1.51*	0.00044	1.52*	0.00015	ND		ND		1.49*	0.00002
PARP12	218543_s_at	poly (ADP-ribose) polymerase family, member 12	3445	2.44	0.00000	1.79	0.00000	2.31	0.00000	1.77	0.00000	1.77	0.00000	1.91	0.00000	1.94	0.00000
PARVB	216253_s_at	parvin, beta	349	-1.1	0.28329	-1.36	0.00957	-1.1	0.29959	-1.18	0.08023	-1.52	0.00008	-1.21	0.07586	-1.28	0.01914
PCTP	218676_s_at	phosphatidylcholine transfer protein	518	-1.29	0.01758	-1.64	0.00058	-1.31	0.05446	-1.07	0.56230	1.04	0.85209	1.17	0.62394	1.76	0.16714
PDGFC	218718_at	platelet derived growth factor C	1240	-1.21	0.01160	-1.51	0.00010	-1.31	0.00241	-1.57	0.00004	-1.4	0.00025	-1.33	0.00103	-1.27	0.00560
PDHB	211023_at	pyruvate dehydrogenase (lipoamide) beta	3815	1	0.95283	-1.13	0.25000	-1.26	0.03026	-1.14	0.16995	-1.28	0.01524	-1.13	0.25622	-1.57	0.00047
PF4	206390_x_at	platelet factor 4 (chemokine (C-X-C motif) ligand 4)	10386	1.7	0.00028	1.04	0.62465	1.62	0.00042	-1.13	0.14207	-1.12	0.13682	-1.13	0.33129	1.24	0.04945
PGAP1	213469_at	GPI deacylase	67	2.00*	0.00002	1.80*	0.00021	2.27*	0.00000	ND		1.98*	0.00000	ND		2.04*	0.00000
PGAP1	220576_at	GPI deacylase	239	1.91	0.00000	1.7	0.00245	2.19	0.00068	1.23	0.00341	1.83	0.00001	1.82	0.00002	2.01	0.00014
PHF11	221816_s_at	PHD finger protein 11	5834	1.8	0.00000	1.59	0.00000	1.77	0.00000	1.6	0.00000	1.66	0.00000	1.7	0.00000	1.7	0.00000
PHLDA2	209803_s_at	pleckstrin homology-like domain, family A, member 2	215	1.64	0.12643	1.34	0.09865	1.51	0.04184	1.18	0.37317	1.43	0.02906	1.28	0.05610	1.87	0.00059
PID1	219093_at	phosphotyrosine interaction domain containing 1	1358	-1.96	0.00005	-1.75	0.00019	-2.29	0.00002	-1.76	0.00103	-1.82	0.00007	-1.87	0.00011	-1.9	0.00038
PILRA	219788_at	paired immunoglobin-like type 2 receptor alpha	1272	1.33	0.00144	-1.11	0.18600	-1.07	0.38478	-1.27	0.00560	-1.56	0.00000	-1.44	0.00163	-1.48	0.00003
PIP4K2A	212829_at	phosphatidylinositol-5-phosphate 4-kinase, type II, alpha	7793	-1.03	0.39496	1.01	0.84386	1.03	0.53862	1.22	0.09077	1.24	0.00546	1.29	0.01886	1.58	0.00017
PLAC8	219014_at	placenta-specific 8	14056	1.85	0.00000	1.3	0.00007	1.71	0.00000	1.36	0.00001	1.35	0.00020	1.32	0.00037	1.4	0.00000
PLSCR1	202446_s_at	phospholipid scramblase 1	3924	3.06	0.00000	1.93	0.00000	2.8	0.00000	2.08	0.00000	2.06	0.00000	2.06	0.00000	2.36	0.00000
PLSCR1	202430_s_at	phospholipid scramblase 1	934	2.6	0.00000	1.73	0.00000	2.39	0.00000	2.02	0.00000	1.81	0.00000	1.83	0.00001	1.88	0.00000
PML /// LOC161527	211012_s_at	promyelocytic leukemia /// hypothetical protein LOC161527	745	2.34*	0.00000	1.61*	0.00003	2.11*	0.00000	ND		ND		ND		1.55*	0.00260
POU2AF1	205267_at	POU class 2 associating factor 1	2449	-1.51	0.00002	-1.14	0.01881	-1.44	0.00008	-1.12	0.19448	1.04	0.58193	-1.07	0.61991	-1.15	0.24413
PPBP	214146_s_at	pro-platelet basic protein (chemokine (C-X-C motif) ligand 7)	17295	1.19	0.00770	-1.4	0.00008	1.03	0.69427	-1.71	0.00001	-1.61	0.00001	-1.57	0.00057	-1.15	0.15638
PPM1F	37384_at	protein phosphatase 1F (PP2C domain containing)	2234	-1.43	0.00008	-1.48	0.00018	-1.66	0.00000	-1.42	0.00308	-1.46	0.00042	-1.29	0.00440	-1.45	0.00007
PPM1F	203063_at	protein phosphatase 1F (PP2C domain containing)	2364	-1.55	0.00153	-1.61	0.00150	-1.84	0.00040	-1.58	0.00478	-1.81	0.00051	-1.37	0.02372	-1.62	0.00057
PPM2C	218273_s_at	protein phosphatase 2C, magnesium-dependent, catalytic subunit	1016	-1.24	0.00162	-1.17	0.04965	-1.22	0.01531	-1.49	0.00046	-1.31	0.01081	-1.51	0.00029	-1.34	0.00479
PRDX6	200844_s_at	peroxiredoxin 6	3289	1.01	0.90005	-1.08	0.07951	1.13	0.01382	1.22	0.09326	1.38	0.00503	1.37	0.02077	1.75	0.00054
PRKAR2B	203680_at	protein kinase, cAMP-dependent, regulatory, type II, beta	2192	1.18	0.13891	-1.46	0.00241	1.12	0.37012	-1.69	0.00075	-1.6	0.00073	-1.5	0.00834	-1.11	0.40896

			Day 1	Day 3		Day 6		Day 10		Day 13		Day 27		Day 42		Day 70	
Gene Symbol	Probe Set ID	Gene Title	mean signal	Fold Chg	p-value	Fold Chg	p-value	Fold Chg	p-value	Fold Chg	p-value	Fold Chg	p-value	Fold Chg	p-value	Fold Chg	p-value
PRKCD	202545_at	protein kinase C, delta	2771	1.1	0.06279	-1.16	0.11778	-1.08	0.24598	-1.21	0.05144	-1.62	0.00000	-1.38	0.00360	-1.47	0.00037
PTAFR	206278_at	platelet-activating factor receptor	419	-1.21	0.04324	-1.37	0.01114	-1.53	0.00058	-1.60#	0.00084	-1.86#	0.00000	-1.63#	0.00028	-1.81#	0.00032
PTCH1	209815_at	patched homolog 1 (Drosophila)	666	1.22	0.00128	1.43	0.00025	1.26	0.00705	1.61	0.00056	1.22	0.02253	1.17	0.10535	1.06	0.61365
PTOV1	212032_s_at	prostate tumor overexpressed gene 1	704	-1.47#	0.00359	-1.12	0.30765	-1.54#	0.00410	-1.42#	0.00230	-1.59#	0.00097	-1.54#	0.01858	-1.62#	0.00048
PTPRO	208121_s_at	protein tyrosine phosphatase, receptor type, O	460	1.72	0.00001	1.22	0.03919	1.45	0.01164	1.2	0.10154	1.1	0.24103	1.12	0.15811	-1.05	0.40871
QARS	217846_at	glutaminyl-tRNA synthetase	6877	-1.56	0.00000	-1.38	0.00000	-1.51	0.00000	-1.33	0.00000	-1.33	0.00000	-1.3	0.00001	-1.4	0.00000
RAB31	217763_s_at	RAB31, member RAS oncogene family	3665	-1.22	0.00393	-1.53	0.00001	-1.35	0.00002	-1.44	0.00001	-1.48	0.00001	-1.35	0.00239	-1.21	0.03929
RABGAP1L	213982_s_at	RAB GTPase activating protein 1-like	2002	1.61	0.00000	1.17	0.00454	1.41	0.00000	1.22	0.00528	1.14	0.02797	1.22	0.00395	1.14	0.06268
RBX1	218117_at	ring-box 1	3462	1.46	0.00000	1.2	0.01619	1.5	0.00000	1.41	0.00019	1.4	0.00004	1.4	0.00011	1.64	0.00000
RGL1	209568_s_at	ral guanine nucleotide dissociation stimulator-like 1	659	1.54	0.00000	1.28	0.00334	1.56	0.00002	1.22	0.00839	1.3	0.00002	1.42	0.00013	1.27	0.00003
RGS1	216834_at	regulator of G-protein signaling 1	4361	-1.43	0.06423	-1.23	0.40107	-1.43	0.13300	-1.71	0.01831	-1.33	0.10865	-1.78	0.02656	-2.36	0.00094
RGS2	202388_at	regulator of G-protein signaling 2, 24kDa	19526	-1.34	0.00201	-1.42	0.00011	-1.79	0.00000	-1.57	0.00009	-1.5	0.00004	-1.56	0.00002	-1.73	0.00000
RHOB	212099_at	ras homolog gene family, member B	4049	1.65	0.00059	1.37	0.09397	1.18	0.32632	1.17	0.40469	1.19	0.34697	-1.08	0.70536	1.07	0.60849
RHOBTB1	212651_at	Rho-related BTB domain containing 1	102	2.50*	0.00009	ND		2.41*	0.00065	ND		ND		ND		ND	
RIN2	209684_at	Ras and Rab interactor 2	947	1.53	0.00010	1.01	0.93518	1.35	0.00395	1.11	0.15205	-1.02	0.68325	1.08	0.44990	1.07	0.36964
RNASE1	201785_at	ribonuclease, RNase A family, 1 (pancreatic)	495	1.32*	0.01349	1.28*	0.05466	1.66*	0.00061	ND		1.50*	0.01894	1.59*	0.00871	2.12*	0.00267
RNASE2	206111_at	ribonuclease, RNase A family, 2 (liver, eosinophil-derived neurotoxin)	3620	2.86	0.00000	1.85	0.00001	2.32	0.00000	1.76	0.00132	1.65	0.00002	1.73	0.00045	1.94	0.00000
ROGDI	218394_at	rogdi homolog (Drosophila)	1530	-1.28	0.00001	-1.52	0.00000	-1.4	0.00002	-1.26	0.00062	-1.38	0.00002	-1.23	0.00269	-1.27	0.00627
RP2	205191_at	retinitis pigmentosa 2 (X-linked recessive)	346	1.05	0.60752	-1.29	0.02624	-1.1	0.36854	-1.16	0.07932	-1.41	0.00011	-1.16	0.09760	-1.52	0.00001
RPH3A	205230_at	rabphilin 3A homolog (mouse)	523	-1.79	0.00188	-1.71	0.00099	-1.86	0.00054	-1.68	0.00721	-1.52	0.00212	-1.68	0.00095	-1.64	0.00524
RPIA	212973_at	ribose 5-phosphate isomerase A (ribose 5-phosphate epimerase)	2172	-1.08	0.18904	1.02	0.62404	1.06	0.17144	1.37	0.05336	1.42	0.00048	1.43	0.01382	1.63	0.00085
RPL10L	217559_at	ribosomal protein L10-like	297	-1.76#	0.00058	-1.55	0.00041	-1.11	0.23911	-1.25#	0.11655	1.07	0.52035	-1.21#	0.10899	-1	0.96511
RPL18A /// hCG_2040224 /// LOC285053 /// LOC390354 /// LOC729955	216383_at	60S ribosomal protein L18a /// similar to ribosomal protein L18a;	988	-1.48	0.00179	-1.08	0.39132	-1.4	0.01108	-1.33	0.05189	-1.24	0.06867	-1.57	0.00229	-1.67	0.00013
RPL22	214042_s_at	ribosomal protein L22	9528	-1.52	0.00000	-1.2	0.00051	-1.4	0.00000	-1.19	0.00400	-1.13	0.07384	-1.22	0.00069	-1.22	0.00041
RPL3	211666_x_at	ribosomal protein L3	41213	-1.51	0.00000	-1.18	0.00000	-1.35	0.00000	-1.23	0.00017	-1.12	0.00371	-1.23	0.00000	-1.22	0.00000
RPL4	211710_x_at	ribosomal protein L4	40945	-1.58	0.00000	-1.27	0.00000	-1.5	0.00000	-1.34	0.00002	-1.22	0.00010	-1.26	0.00006	-1.36	0.00000
RPL4	201154_x_at	ribosomal protein L4	41802	-1.63	0.00000	-1.27	0.00000	-1.55	0.00000	-1.36	0.00004	-1.27	0.00003	-1.3	0.00016	-1.42	0.00000
RPL4	200089_s_at	ribosomal protein L4	19883	-1.56	0.00000	-1.23	0.00000	-1.45	0.00000	-1.35	0.00001	-1.25	0.00009	-1.32	0.00005	-1.43	0.00000
RPS5 /// LOC392424	200024_at	ribosomal protein S5 /// similar to ribosomal protein S5	27131	-1.56	0.00000	-1.26	0.00033	-1.46	0.00000	-1.28	0.00046	-1.12	0.01883	-1.18	0.00523	-1.23	0.00295
RRAS	212647_at	related RAS viral (r-ras) oncogene homolog	610	1.92	0.00000	-1.02	0.87555	1.46	0.00231	1.16	0.19707	-1.01	0.92829	1.12	0.38602	1.11	0.46655
RSAD2	213797_at	radical S-adenosyl methionine domain containing 2	616	12.78	0.00000	5.26	0.00000	11.56	0.00000	6.79	0.00000	6.43	0.00005	6.42	0.00000	6.59	0.00000
RSL1D1	212019_at	ribosomal L1 domain containing 1	399	-1.38#	0.00029	-1.13#	0.09480	-1.50#	0.00004	-1.12	0.11135	-1.14	0.10320	-1.13#	0.14310	-1.19	0.03466
RTN1	210222_s_at	reticulon 1	1330	-2.05	0.00000	-1.97	0.00000	-2.15	0.00000	-1.93	0.00000	-2.15	0.00000	-1.93	0.00000	-2.05	0.00000
RTN1	203485_at	reticulon 1	985	-2.17	0.00000	-1.94	0.00004	-2.29	0.00000	-1.96	0.00001	-1.99	0.00000	-1.98	0.00000	-2.25	0.00000
RTN2	34408_at	reticulon 2	492	-1.11	0.16442	-1.53	0.00019	-1.07	0.33146	-1.18	0.08834	-1.14	0.09135	-1.06	0.46576	1.01	0.89214
RTP4	219684_at	receptor (chemosensory) transporter protein 4	638	3.13*	0.00000	1.71*	0.00307	2.59*	0.00005	2.04*	0.00002	ND		1.72*	0.00193	ND	
SAMD4A	215495_s_at	sterile alpha motif domain containing 4A	167	2.56*	0.00000	ND		2.03*	0.00000	ND		ND		ND		ND	
SAMD4A	212845_at	sterile alpha motif domain containing 4A	541	2.77	0.00000	1.35	0.00423	2.18	0.00000	1.56	0.00038	1.32	0.02468	1.56	0.00346	1.48	0.00027
SAMD9	219691_at	sterile alpha motif domain containing 9	514	3.82	0.00000	2.1	0.00001	3.26	0.00000	2.62	0.00000	2.05	0.00015	2.12	0.00004	2.01	0.00001
SCO2	205241_at	SCO cytochrome oxidase deficient homolog 2 (yeast)	3018	2.28	0.00000	1.7	0.00020	2.03	0.00001	1.61	0.00580	1.58	0.00223	1.71	0.00017	1.94	0.00000
SEMA3C	203789_s_at	sema domain, immunoglobulin domain (Ig), short basic domain, secreted, (semaphorin) 3C	194	-1.66	0.00002	-1.4	0.00700	-1.5	0.00005	-1.39	0.00296	-1.48	0.00037	-1.36	0.00215	-1.5	0.00005
SERPING1	200986_at	serpin peptidase inhibitor, clade G (C1 inhibitor), member 1, (angioedema, hereditary)	415	4.93	0.00000	1.87	0.00015	4.07	0.00000	2.25	0.00008	1.9	0.00062	1.99	0.00029	2.12	0.00000
SIGLEC1	219519_s_at	sialic acid binding Ig-like lectin 1, sialoadhesin	183	27.14*	0.00000	16.48*	0.00000	23.13*	0.00000	17.00*	0.00000	13.68*	0.00000	18.17*	0.00000	20.93*	0.00000
SIGLEC1	44673_at	sialic acid binding Ig-like lectin 1, sialoadhesin	679	6.41	0.00000	4.28	0.00000	5.54	0.00000	4.33	0.00000	3.55	0.00000	4.57	0.00000	4.88	0.00000
SKAP2	204361_s_at	src kinase associated phosphoprotein 2	775	-1.07	0.22501	-1.26	0.00617	-1.16	0.00805	-1.31	0.00232	-1.38	0.00004	-1.52	0.00004	-1.34	0.00010
SLC14A1	205856_at	solute carrier family 14 (urea transporter), member 1 (Kidd blood group)	124	-1.24	0.02589	1.07	0.47745	1.21	0.11107	1.85	0.14698	1.91	0.04938	2.05	0.01073	2.19	0.00066
SLC25A6	212826_s_at	solute carrier family 25 (mitochondrial carrier; adenine nucleotide translocator), member 6	18507	-1.4	0.00001	-1.36	0.00061	-1.58	0.00000	-1.64	0.00000	-1.43	0.00003	-1.42	0.00001	-1.46	0.00000
SLC25A6	212085_at	solute carrier family 25 (mitochondrial carrier; adenine nucleotide translocator), member 6	16829	-1.57	0.00000	-1.44	0.00000	-1.59	0.00000	-1.52	0.00000	-1.42	0.00000	-1.5	0.00000	-1.55	0.00000
SLC31A2	204204_at	solute carrier family 31 (copper transporters), member 2	2025	1.74	0.00000	-1.17	0.08792	1.41	0.00051	1.08	0.35602	-1.09	0.08252	1.08	0.48747	-1.05	0.54131
SLC7A7	204588_s_at	solute carrier family 7 (cationic amino acid transporter, y+ system), member 7	8576	-1.07	0.19910	-1.51	0.00000	-1.29	0.00011	-1.49	0.00001	-1.64	0.00000	-1.41	0.00041	-1.57	0.00000

			Day 1	Day 3		Day 6		Day 10		Day 13		Day 27		Day 42		Day 70	
Gene Symbol	Probe Set ID	Gene Title	mean signal	Fold Chg	p-value	Fold Chg	p-value	Fold Chg	p-value	Fold Chg	p-value	Fold Chg	p-value	Fold Chg	p-value	Fold Chg	p-value
SMCHD1	212569_at	structural maintenance of chromosomes flexible hinge domain containing 1	1402	1.66	0.00000	1.49	0.00003	1.63	0.00002	1.3	0.01137	1.4	0.00018	1.33	0.00097	1.42	0.00099
SMCHD1	212579_at	structural maintenance of chromosomes flexible hinge domain containing 1	1697	1.53	0.00000	1.37	0.00001	1.54	0.00002	1.33	0.00032	1.31	0.00000	1.26	0.00126	1.41	0.00013
SMCHD1	212577_at	structural maintenance of chromosomes flexible hinge domain containing 1	3613	1.58	0.00000	1.48	0.00000	1.57	0.00000	1.34	0.00055	1.36	0.00000	1.22	0.02942	1.27	0.00553
SMOX	210357_s_at	spermine oxidase	569	1.84*	0.00035	1.29*	0.02582	1.92*	0.00011	1.30*	0.18036	1.39*	0.04419	1.63*	0.01551	2.03*	0.00110
SMPDL3A	213624_at	sphingomyelin phosphodiesterase, acid-like 3A	762	1.23	0.02865	-1.45	0.00346	-1.29	0.00986	-1.47	0.00142	-1.66	0.00001	-1.41	0.00096	-1.43	0.00088
SNAP23	209131_s_at	synaptosomal-associated protein, 23kDa	626	1.01	0.92998	-1.16	0.07157	-1.07	0.25177	-1.41	0.01308	-1.31	0.03291	-1.63	0.00053	-1.49	0.00350
SOBP	218974_at	sine oculis binding protein homolog (Drosophila)	233	1.41	0.00021	1.35	0.01005	1.56	0.00001	1.44	0.00063	1.41	0.00016	1.41	0.00117	1.33	0.00545
SOD2	215223_s_at	superoxide dismutase 2, mitochondrial	2499	-1.11	0.41139	-1.57	0.01628	-1.38	0.03394	-1.7	0.00276	-1.41	0.00707	-1.52	0.00055	-1.1	0.52876
SOX15	217040_x_at	SRY (sex determining region Y)-box 15	820	ND		ND		1.17*	0.05558	ND		1.49*	0.00386	1.39*	0.06922	1.71*	0.00048
SOX4	201417_at	SRY (sex determining region Y)-box 4	721	-1.37	0.00016	-1.23	0.03696	-1.46	0.00003	-1.48	0.00016	-1.47	0.00011	-1.69	0.00007	-1.38	0.00050
SP100	210218_s_at	SP100 nuclear antigen	348	1.85	0.00000	1.66	0.00006	1.89	0.00000	1.64	0.00006	1.64	0.00001	1.5	0.00001	1.75	0.00000
SP100	202864_s_at	SP100 nuclear antigen	2387	1.93	0.00000	1.77	0.00002	1.83	0.00000	1.44	0.00028	1.62	0.00003	1.54	0.00005	1.6	0.00006
SP100	202863_at	SP100 nuclear antigen	2231	1.77	0.00000	1.52	0.00001	1.73	0.00000	1.37	0.00012	1.44	0.00000	1.45	0.00000	1.55	0.00000
SP100	210985_s_at	SP100 nuclear antigen	175	1.66	0.00002	1.56	0.00056	1.82	0.00002	1.43	0.00881	1.46	0.00130	1.33	0.00559	1.37	0.00104
SP110	208012_x_at	SP110 nuclear body protein	5268	1.94	0.00000	1.46	0.00000	1.85	0.00000	1.52	0.00001	1.54	0.00000	1.5	0.00000	1.59	0.00000
SP110	209762_x_at	SP110 nuclear body protein	4433	1.87	0.00000	1.4	0.00011	1.81	0.00000	1.57	0.00000	1.53	0.00000	1.58	0.00000	1.56	0.00000
SP110	208392_x_at	SP110 nuclear body protein	1737	1.72	0.00000	1.46	0.00004	1.77	0.00000	1.44	0.00000	1.57	0.00001	1.39	0.00030	1.5	0.00000
SP110	209761_s_at	SP110 nuclear body protein	5876	1.7	0.00000	1.44	0.00000	1.63	0.00000	1.3	0.00042	1.37	0.00001	1.29	0.00075	1.41	0.00000
SPTLC2	203127_s_at	serine palmitoyltransferase, long chain base subunit 2	2699	1.56	0.00000	1.13	0.04549	1.5	0.00005	1.21	0.03547	1.18	0.00356	1.31	0.00200	1.35	0.00036
SPTLC2	216202_s_at	serine palmitoyltransferase, long chain base subunit 2	1091	1.92	0.00000	1.16	0.15527	1.73	0.00072	1.26	0.04730	1.07	0.56961	1.27	0.08534	1.29	0.05098
SSPN	204963_at	sarcospan (Kras oncogene-associated gene)	134	-1.52#	0.00304	-1.08#	0.42101	-1.80#	0.00065	-1.00#	0.99059	-1.62#	0.00191	-1.17#	0.20517	-1.65#	0.00549
STAP1	220059_at	signal transducing adaptor family member 1	629	1.32	0.00503	1.4	0.00822	1.46	0.00786	1.41	0.01629	1.58	0.00006	1.32	0.01030	1.84	0.00013
STAT1	AFFX-HUMISGF	signal transducer and activator of transcription 1, 91kDa	1305	2.46	0.00000	1.54	0.00007	2.42	0.00000	1.65	0.00033	1.49	0.00174	1.37	0.01116	1.47	0.00171
STAT1	209969_s_at	signal transducer and activator of transcription 1, 91kDa	2479	2.37	0.00000	1.45	0.00077	2.26	0.00000	1.4	0.00143	1.46	0.00107	1.52	0.00658	1.44	0.00194
STAT1	AFFX-HUMISGF3A /M97935_3_at	signal transducer and activator of transcription 1, 91kDa	4980	1.78	0.00000	1.35	0.00001	1.76	0.00000	1.43	0.00014	1.41	0.00001	1.41	0.00001	1.39	0.00000
STAT1	AFFX-HUMISGF	signal transducer and activator of transcription 1, 91kDa	569	2.56	0.00000	1.7	0.00068	2.71	0.00001	1.72	0.00261	1.58	0.01509	1.33	0.04333	1.39	0.04431
STAT1	AFFX-HUMISGF	signal transducer and activator of transcription 1, 91kDa	2027	2.57	0.00000	1.66	0.00002	2.48	0.00000	1.73	0.00030	1.46	0.00327	1.36	0.02601	1.38	0.00945
STAT1	200887_s_at	signal transducer and activator of transcription 1, 91kDa	9193	1.69	0.00000	1.3	0.00006	1.7	0.00000	1.41	0.00014	1.38	0.00008	1.45	0.00000	1.36	0.00009
STAT2	205170_at	signal transducer and activator of transcription 2, 113kDa	637	1.69	0.00102	1.22	0.06832	1.7	0.00015	1.13	0.14572	1.19	0.11073	-1.04	0.73713	1.2	0.06244
TAL1	216925_s_at	T-cell acute lymphocytic leukemia 1	274	ND		ND		1.43*	0.04380	1.63*	0.07204	2.09*	0.00160	1.87*	0.05818	2.71*	0.00055
TAP1	202307_s_at	transporter 1, ATP-binding cassette, sub-family B (MDR/TAP)	3093	2.09	0.00000	1.37	0.00006	1.93	0.00000	1.47	0.00159	1.22	0.08957	1.39	0.00689	1.29	0.01117
TBC1D22A	210144_at	TBC1 domain family, member 22A	703	-1.3	0.00039	-1.24	0.02330	-1.32	0.00173	-1.27	0.00397	-1.48	0.00026	-1.48	0.00005	-1.89	0.00000
TBC1D9	212956_at	TBC1 domain family, member 9 (with GRAM domain)	2064	-1.51	0.00006	-1.55	0.00001	-1.69	0.00000	-1.38	0.00056	-1.45	0.00000	-1.49	0.00002	-1.76	0.00000
TBCD	201759_at	tubulin folding cofactor D	340	-1.31#	0.01483	-1.23	0.05912	1.03	0.83393	1.19	0.37024	1.64	0.00046	1.47	0.01017	1.52	0.00091
TBXAS1	208130_s_at	thromboxane A synthase 1 (platelet, cytochrome P450, family 5, subfamily A)	1567	-1.08	0.36451	-1.3	0.00532	-1.27	0.00561	-1.28	0.01657	-1.57	0.00004	-1.3	0.04686	-1.29	0.02243
TCN2	204043_at	transcobalamin II; macrocytic anemia	552	2.16	0.00000	1.24	0.00537	1.68	0.00000	1.31	0.00875	1.3	0.00920	1.45	0.00096	1.42	0.00005
TDRD7	213361_at	tudor domain containing 7	1338	1.64	0.00000	1.28	0.00000	1.6	0.00000	1.49	0.00000	1.39	0.00004	1.46	0.00000	1.46	0.00000
TFDP2	203588_s_at	transcription factor Dp-2 (E2F dimerization partner 2)	490	1.14	0.04720	1.14	0.15297	1.22	0.03339	1.27	0.01546	1.51	0.00001	1.37	0.01297	1.54	0.00117
TK2	204276_at	thymidine kinase 2, mitochondrial	503	1.03	0.67289	-1.09	0.39985	-1.21	0.03306	-1.05	0.61286	-1.19	0.04185	-1.16	0.04035	-1.51	0.00032
TKT	208699_x_at	transketolase (Wernicke-Korsakoff syndrome)	9769	-1.3	0.00004	-1.53	0.00000	-1.51	0.00000	-1.45	0.00001	-1.61	0.00000	-1.54	0.00001	-1.48	0.00000
TKT	208700_s_at	transketolase (Wernicke-Korsakoff syndrome)	10936	-1.18	0.02885	-1.33	0.00620	-1.35	0.00179	-1.43	0.00007	-1.51	0.00043	-1.5	0.00009	-1.58	0.00000
TLR1	210176_at	toll-like receptor 1	846	1.59	0.00032	-1.06	0.52935	1.23	0.03395	1.02	0.79288	-1.01	0.91169	1.09	0.38678	-1.07	0.43712
TLR3	206271_at	toll-like receptor 3	147	1.50*	0.00319	1.34*	0.03399	1.49*	0.00695	1.54*	0.00070	ND		ND		1.13*	0.31138
TLR7	220146_at	toll-like receptor 7	399	2.24	0.00001	1.17	0.22160	1.96	0.00025	1.39	0.06554	1	0.99649	1.33	0.02171	1.01	0.91565
TMED2	204426_at	transmembrane emp24 domain trafficking protein 2	667	-1.17	0.08846	-1.11	0.22827	-1.11	0.27462	-1.34	0.02184	-1.18	0.13850	-1.76	0.00032	-1.34	0.03497
TMEM140	218999_at	transmembrane protein 140	1049	2.41	0.00000	1.31	0.00094	2.22	0.00000	1.31	0.00420	1.2	0.05455	1.35	0.00928	1.54	0.00073
TMOD3	220800_s_at	tropomodulin 3 (ubiquitous)	169	1.63	0.00055	1.39	0.04117	1.48	0.00071	1.39	0.02636	1.31	0.09044	1.01	0.90932	1.16	0.37191
TNFAIP6	206025_s_at	tumor necrosis factor, alpha-induced protein 6	233	1.87*	0.00322	ND		1.51*	0.00051	ND		ND		ND		ND	
TNFAIP6	206026_s_at	tumor necrosis factor, alpha-induced protein 6	231	2.07	0.00099	-1	0.99210	1.59	0.00253	1.2	0.29455	1.1	0.54454	1.09	0.56358	1.08	0.63088

			Day 1	Day 3		Day 6		Day 10		Day 13		Day 27		Day 42		Day 70	
Gene Symbol	Probe Set ID	Gene Title	mean signal	Fold Chg	p-value	Fold Chg	p-value	Fold Chg	p-value	Fold Chg	p-value	Fold Chg	p-value	Fold Chg	p-value	Fold Chg	p-value
TNFRSF25	211841_s_at	tumor necrosis factor receptor superfamily, member 25	683	1.15	0.07056	1.51	0.00002	1.35	0.00044	1.59	0.00034	1.44	0.00003	1.55	0.01370	1.46	0.00076
TNFRSF8	206729_at	tumor necrosis factor receptor superfamily, member 8	658	1	0.96923	-1.49	0.00039	-1.34	0.00732	-1.64	0.00003	-1.61	0.00000	-1.6	0.00005	-1.7	0.00000
TNFSF10	202687_s_at	tumor necrosis factor (ligand) superfamily, member 10	2896	2.34	0.00000	1.16	0.06597	1.95	0.00000	1.4	0.00149	1.1	0.27355	1.11	0.40561	1.11	0.43599
TNFSF10	202688_at	tumor necrosis factor (ligand) superfamily, member 10	7233	2.04	0.00000	1.06	0.46959	1.59	0.00002	1.17	0.03032	-1.01	0.87814	1.09	0.39137	1.08	0.48364
TNFSF10	214329_x_at	tumor necrosis factor (ligand) superfamily, member 10	1467	2.01	0.00000	-1	0.99181	1.72	0.00182	1.29	0.07697	-1.16	0.22638	1.16	0.30896	-1.19	0.25887
TNS1	221748_s_at	tensin 1	562	1.4	0.00545	1.08	0.53973	1.7	0.00001	2.37	0.07299	2.29	0.00167	2.6	0.02500	3.26	0.00243
TOB2	222243_s_at	transducer of ERBB2, 2	702	-1.1	0.20294	-1.05	0.55289	-1.15	0.05550	-1.02	0.81786	-1.39	0.00027	-1.2	0.01561	-1.71	0.00000
TOR1B	209593_s_at	torsin family 1, member B (torsin B)	1585	1.89	0.00000	1.29	0.00026	1.65	0.00000	1.53	0.00000	1.3	0.00191	1.39	0.00448	1.35	0.00043
TRAFD1	202837_at	TRAF-type zinc finger domain containing 1	387	1.99*	0.00001	ND		1.37*	0.04119	ND		ND		ND		ND	
TRAK2	202125_s_at	trafficking protein, kinesin binding 2	1770	1.04	0.24888	1.08	0.07092	1.16	0.00112	1.41	0.00617	1.5	0.00010	1.62	0.00022	1.67	0.00064
TREX1	205875_s_at	three prime repair exonuclease 1	1871	1.97*	0.00000	1.13*	0.08996	1.79*	0.00000	1.32*	0.00683	ND		1.36*	0.00350	1.35*	0.00137
TRIM14	203148_s_at	tripartite motif-containing 14	2531	1.51	0.00001	1.29	0.00952	1.4	0.00053	1.33	0.00121	1.13	0.14676	1.22	0.01181	1.06	0.34513
TRIM21	204804_at	tripartite motif-containing 21	1531	1.64	0.00002	1.2	0.05582	1.69	0.00012	1.4	0.00472	1.19	0.06869	1.27	0.03776	1.23	0.04461
TRIM22	213293_s_at	tripartite motif-containing 22	10465	1.72	0.00000	1.45	0.00000	1.65	0.00000	1.47	0.00000	1.46	0.00000	1.41	0.00000	1.5	0.00000
TRIM38	203567_s_at	tripartite motif-containing 38	3072	1.63	0.00000	1.32	0.00002	1.5	0.00000	1.34	0.00001	1.29	0.00004	1.21	0.00418	1.23	0.00274
TRIM38	203610_s_at	tripartite motif-containing 38	1396	1.82	0.00000	1.35	0.00003	1.55	0.00000	1.34	0.00016	1.19	0.00987	1.1	0.17158	1.13	0.06292
TRIM5	210705_s_at	tripartite motif-containing 5	459	1.81	0.00003	1.22	0.08498	1.55	0.00183	1.46	0.00592	1.34	0.00791	1.34	0.01796	1.24	0.08066
TRIM58	215047_at	tripartite motif-containing 58	726	1.73	0.05524	-1.12	0.72417	2.53	0.00073	2.6	0.04365	4	0.00094	3.28	0.00928	4.16	0.00018
TRIM6-TRIM34	221044_s_at	tripartite motif-containing 6 and tripartite motif-containing 34	1196	1.69	0.00000	1.29	0.00342	1.51	0.00006	1.35	0.00013	1.23	0.00110	1.23	0.02391	1.17	0.06064
TRIP6	209129_at	thyroid hormone receptor interactor 6	245	1.57	0.00001	1.37	0.00086	1.41	0.00034	1.28	0.00661	1.35	0.00225	1.42	0.00115	1.28	0.00085
TSC22D1	215111_s_at	TSC22 domain family, member 1	2072	1.33	0.00448	1.24	0.01004	1.57	0.00029	1.07	0.37527	1.21	0.00656	1.23	0.00487	1.69	0.00016
TSC22D3	207001_x_at	TSC22 domain family, member 3	2220	-1.53	0.00869	1.09	0.66441	-1.13	0.51701	-2.03	0.00053	-1.65	0.00519	-1.83	0.00665	-1.57	0.01786
TUBA1A	209118_s_at	tubulin, alpha 1a	7743	-1.2	0.00212	-1.36	0.00081	-1.4	0.00015	-1.7	0.00022	-1.42	0.00022	-1.43	0.00002	-1.4	0.00002
TUBB1	208601_s_at	tubulin, beta 1	1467	1.57	0.02238	-1.18	0.30296	1.05	0.71619	-1.38	0.04566	-1.68#	0.00089	-1.27	0.22907	-1.01	0.94267
TXNDC5	221253_s_at	thioredoxin domain containing 5	3419	-1.43	0.00657	-1.22	0.14290	-1.78	0.00053	-1.41	0.04817	-1.48	0.02379	-1.4	0.16100	-1.85	0.00559
TYMS	202589_at	thymidylate synthetase	744	1.25	0.03261	1.22	0.10831	1.37	0.01644	1.19	0.12184	1.3	0.01334	1.48	0.00068	1.5	0.00039
UBAP2L	201377_at	ubiquitin associated protein 2-like	1133	1.07	0.22277	1.26	0.00480	1.23	0.00520	1.19	0.05869	1.36	0.01194	1.24	0.01020	1.51	0.00008
UBE2L6	201649_at	ubiquitin-conjugating enzyme E2L 6	6323	1.91	0.00000	1.32	0.00001	1.81	0.00000	1.31	0.00093	1.25	0.00218	1.26	0.00085	1.27	0.00008
UBL3	201534_s_at	ubiquitin-like 3	2189	-1.29	0.00801	-1.16	0.26636	-1.33	0.01555	-1.35	0.01097	-1.66	0.00062	-1.61	0.00162	-1.85	0.00002
UNC119B	202365_at	unc-119 homolog B (C. elegans)	995	-1.58	0.00000	-1.28	0.00204	-1.61	0.00000	-1.24	0.00233	-1.22	0.00282	-1.25	0.00078	-1.33	0.00013
UQCRB	209066_x_at	ubiquinol-cytochrome c reductase binding protein	10284	-1.27	0.01217	-1.07	0.52318	-1.36	0.00081	-1.41	0.01819	-1.54	0.00069	-1.53	0.00013	-1.58	0.00003
USP18 /// LOC727996 /// LOC728216 /// LOC728438	219211_at	ubiquitin specific peptidase 18 /// similar to ubiquitin specific peptidase 18	287	7.43*	0.00000	4.93*	0.00000	7.47*	0.00000	5.07*	0.00000	4.50*	0.00000	5.28*	0.00000	5.50*	0.00000
UTRN	213023_at	utrophin	691	1.51	0.00008	1.24	0.00304	1.32	0.00111	1.17	0.04872	1.16	0.13223	-1.04	0.65005	-1.03	0.73095
VDR	204255_s_at	vitamin D (1,25- dihydroxyvitamin D3) receptor	539	1.6	0.00001	1.02	0.80767	1.17	0.04634	1.08	0.33407	1.03	0.66745	1.16	0.25408	1.22	0.06791
VPREB3	220068_at	pre-B lymphocyte gene 3	600	ND		1.44	0.01914	ND		1.46	0.00615	1.5	0.00093	1.48	0.05441	1.28	0.05493
WARS	200629_at	tryptophanyl-tRNA synthetase	6088	1.09	0.26161	-1.44	0.00177	-1.16	0.12151	-1.61	0.00040	-1.52	0.00059	-1.45	0.00407	-1.4	0.01123
WBP5	217975_at	WW domain binding protein 5	158	1.31	0.00146	1.47	0.00005	1.63	0.00021	1.43	0.00250	1.65	0.00006	1.77	0.00002	1.91	0.00000
WDR1	210935_s_at	WD repeat domain 1	992	-1.12	0.11360	-1.26	0.03185	-1.2	0.07726	-1.12	0.22391	-1.35	0.00407	-1.51	0.00066	-1.57	0.00098
WRB	202749_at	tryptophan rich basic protein	1069	-1	0.99843	-1.04	0.63399	-1.11	0.26350	-1.09	0.54838	-1.52	0.00012	-1.26	0.05054	-1.67	0.00012
WSB1	210561_s_at	WD repeat and SOCS box-containing 1	3340	1.52	0.00000	1.19	0.03237	1.37	0.00063	1.2	0.07249	1.12	0.10727	-1.03	0.74941	1.15	0.11347
XAF1	206133_at	XIAP associated factor-1	1551	4.25	0.00000	3.29	0.00000	3.76	0.00000	2.93	0.00000	2.93	0.00001	2.47	0.00000	3.08	0.00000
XK	206698_at	X-linked Kx blood group (McLeod syndrome)	293	1.61	0.00353	1.17	0.15331	2.06	0.00002	2.6	0.03571	3.84	0.00433	4.42	0.01340	5.68	0.00211
ZBP1	208087_s_at	Z-DNA binding protein 1	176	3.03*	0.00000	2.19*	0.00001	3.03*	0.00000	2.22*	0.00001	2.05*	0.00003	2.16*	0.00029	2.21*	0.00001
ZCCHC2	219062_s_at	zinc finger, CCHC domain containing 2	1318	2.26	0.00000	1.89	0.00001	2.24	0.00000	1.68	0.00032	1.79	0.00000	1.84	0.00000	1.76	0.00000
ZEB2	203603_s_at	zinc finger E-box binding homeobox 2	924	1.12	0.20456	-1.26	0.00240	-1.23	0.05835	-1.39	0.00849	-1.47	0.00138	-1.41	0.00221	-1.53	0.00034
ZFX	214678_x_at	zinc finger protein, X-linked	215	-1.15	0.16097	-1.1	0.41807	-1.22	0.06382	-1.45	0.00488	-1.32	0.01256	-1.37	0.00833	-1.54	0.00034
ZNF652	205594_at	zinc finger protein 652	805	-1.48	0.00141	-1.26	0.04962	-1.7	0.00008	-1.28	0.05005	-1.28	0.05052	-1.5	0.00015	-1.48	0.00255
---	212605_s_at	AF034176 Human mRNA (Tripodis and Ragoussis) Homo sapiens cDNA clone ntcon5 contig	1616	-1.6	0.00000	-1.16	0.01702	-1.48	0.00000	-1.36	0.00010	-1.34	0.00002	-1.29	0.00020	-1.35	0.00000
---	221973_at	CDNA clone IMAGE:5217021, with apparent retained intron	701	1.05	0.60288	1.39	0.00090	1.28	0.02683	1.16	0.04008	1.31	0.00514	1.5	0.00032	1.43	0.01323
---	215617_at	CDNA FLJ11754 fis, clone HEMBA1005588	90	3.12*	0.00000	2.38*	0.00003	3.12*	0.00000	2.32*	0.00001	2.57*	0.00000	2.55*	0.00000	2.61*	0.00000
---	213817_at	CDNA FLJ13601 fis, clone PLACE1010069	591	-1.14	0.14782	-1.23	0.05893	-1.24	0.09077	-1.28	0.03051	-1.55	0.00001	-1.18	0.14125	-1.42	0.00004
---	213294_at	Full-length cDNA clone CS0DK002YF13 of HeLa cells Cot 25-normalized of Homo sapiens (human)	2142	3.34	0.00000	2.64	0.00000	3.39	0.00000	2.55	0.00000	2.5	0.00000	2.67	0.00000	2.8	0.00000
---	213710_s_at	Transcribed locus	1343	-1.31	0.00082	-1.21	0.03008	-1.19	0.02478	-1.11	0.34402	-1.13	0.16092	-1.22	0.02284	-1.54	0.00029
---	202015_x_at	---	46	1.38*	0.18350	1.31*	0.13573	1.58*	0.00093	1.18*	0.39859	ND		ND		ND	

			Day 1	Day 3		Day 6		Day 10		Day 13		Day 27		Day 42		Day 70	
**Gene Symbol**	**Probe Set ID**	**Gene Title**	**mean signal**	**Fold Chg**	**p-value**	**Fold Chg**	**p-value**	**Fold Chg**	**p-value**	**Fold Chg**	**p-value**	**Fold Chg**	**p-value**	**Fold Chg**	**p-value**	**Fold Chg**	**p-value**
---	211781_x_at	---	295	1.18*	0.20604	1.33*	0.09583	2.57*	0.00094	4.81*	0.00781	7.37*	0.00100	7.68*	0.01292	11.19*	0.00083
---	217019_at	---	196	-1.63	0.00107	-1.32	0.02501	-1.61#	0.00083	-1.33	0.08376	-1.59#	0.00288	-1.48	0.02907	-1.98#	0.00003
---	211600_at	---	15352	-1.37	0.00534	-1.32	0.02561	-1.53	0.00078	-1.63	0.00107	-1.52	0.00161	-1.77	0.00008	-1.48	0.00174
---	210596_at	---	671	-1.15	0.06996	-1.27	0.01484	-1.18	0.07926	-1.38	0.02024	-1.08	0.40754	-1.29	0.01053	-1.55	0.00056
---	220494_s_at	---	1723	-1.08	0.37671	-1.09	0.38333	-1.13	0.17846	-1.23	0.04707	-1.32	0.01359	-1.31	0.00385	-1.72	0.00026

* Gene turned on: not detected on day 1, but expressed at this time point; fold change may be inaccurate.
# Gene turned off: detected on day 1 but not detected at this time point; fold change may be inaccurate.
ND: gene not detected either at day 1 or this time point.
