# Supplementary material for: Cyclic changes in gene expression induced by Peg-interferon alfa-2b plus ribavirin in peripheral blood monocytes (PBMC) of hepatitis C patients during the first 10 weeks of treatment
Source: J Transl Med. 2008 Nov 5;6:66. doi: 10.1186/1479-5876-6-66 (PMC2613871; doi:10.1186/1479-5876-6-66)
Supplement: Additional file 2 — Comparison of fold changes for genes differentially expressed at day 3 for this study and the ViraHep C study [file 1479-5876-6-66-S2.pdf]

Supplementary Table 2: Comparison of fold-changes at day 3, Pegasys vs. Peg-intron

Gene Symbol	Probe Set ID	Gene Title	Pegasys	Peg-intron
ABCA1	203505_at	ATP-binding cassette, sub-family A (ABC1), member 1	1.43	1.75
ABCC3	208161_s_at	ATP-binding cassette, sub-family C (CFTR/MRP), member 3	-1.16	1.66
ADAR	201786_s_at	adenosine deaminase, RNA-specific	1.58	1.68
ADK	204120_s_at	adenosine kinase	-1.01	-1.54
ADM	202912_at	adrenomedullin	1.67	1.84
AGRN	212285_s_at	agrin	1.82	2.11
AGRN	217419_x_at	agrin	1.60	1.42
AIF1	213095_x_at	allograft inflammatory factor 1	1.50	1.21
AIM2	206513_at	absent in melanoma 2	1.47	1.64
AKAP2 /// PALM2-AKAP2	202760_s_at	A kinase (PRKA) anchor protein 2 /// PALM2-AKAP2 protein	1.42	1.53
APOBEC3A	210873_x_at	apolipoprotein B mRNA editing enzyme, catalytic polypeptide-like 3A	3.23	2.94
APOL1	209546_s_at	apolipoprotein L, 1	1.40	1.42
APOL6	219716_at	apolipoprotein L, 6	1.66	1.43
ARRB1	218832_x_at	arrestin, beta 1	2.04	1.68
ATG12	204833_at	ATG12 autophagy related 12 homolog (S. cerevisiae)	-1.00	1.93
ATOX1	203454_s_at	ATX1 antioxidant protein 1 homolog (yeast)	1.42	1.58
ATXN1	203231_s_at	ataxin 1	-1.27	-1.58
BCL11A	219497_s_at	B-cell CLL/lymphoma 11A (zinc finger protein)	-1.51	-1.09
BLVRA	203771_s_at	biliverdin reductase A	1.91	1.63
BLVRA	211729_x_at	biliverdin reductase A	1.72	1.61
BLVRA	203773_x_at	biliverdin reductase A	1.59	1.55
BST2	201641_at	bone marrow stromal cell antigen 2	2.23	2.06
C11orf2	217969_at	chromosome 11 open reading frame2	-1.42	-1.63
C13orf18	44790_s_at	chromosome 13 open reading frame 18	-1.49	-1.26
C1GALT1	219439_at	core 1 synthase, glycoprotein-N-acetylgalactosamine 3-beta-galactosyltransferase, 1	1.53	1.55
C1GALT1C1	219283_at	C1GALT1-specific chaperone 1	1.47	1.37
C1QA	218232_at	complement component 1, q subcomponent, A chain	1.88	1.63
C1QB	202953_at	complement component 1, q subcomponent, B chain	2.04	1.97
C2	203052_at	complement component 2	1.63	1.66
C2orf47	219176_at	chromosome 2 open reading frame 47	1.57	1.71
C3AR1	209906_at	complement component 3a receptor 1	2.12	2.27
CALML4	64408_s_at	calmodulin-like 4	1.91	1.83
CALML4	221879_at	calmodulin-like 4	1.81	1.72
CASP1	211368_s_at	caspase 1, apoptosis-related cysteine peptidase (interleukin 1, beta, convertase)	1.47	1.36
CASP1	211367_s_at	caspase 1, apoptosis-related cysteine peptidase (interleukin 1, beta, convertase)	1.48	1.34
CASP5	207500_at	caspase 5, apoptosis-related cysteine peptidase	1.68	1.48
CBR1	209213_at	carbonyl reductase 1	1.86	1.73
CCL2	216598_s_at	chemokine (C-C motif) ligand 2	15.11	13.36
CCR1	205099_s_at	chemokine (C-C motif) receptor 1	2.10	2.06
CCR1	205098_at	chemokine (C-C motif) receptor 1	2.55	1.89
CCR5	206991_s_at	chemokine (C-C motif) receptor 5	1.52	1.43
CD164	208653_s_at	CD164 molecule, sialomucin	1.59	1.36
CD1C	205987_at	CD1c molecule	-1.76	-2.51
CD2AP	203593_at	CD2-associated protein	1.31	1.63
CD38	205692_s_at	CD38 molecule	1.68	1.95
CD44	212014_x_at	CD44 molecule (Indian blood group)	-1.23	-1.50
CD44	209835_x_at	CD44 molecule (Indian blood group)	-1.18	-1.56
CD86	205685_at	CD86 molecule	1.53	1.33
CDKN1A	202284_s_at	cyclin-dependent kinase inhibitor 1A (p21, Cip1)	1.72	1.50
CDKN1C	216894_x_at	cyclin-dependent kinase inhibitor 1C (p57, Kip2)	2.04	2.21
CDKN1C	219534_x_at	cyclin-dependent kinase inhibitor 1C (p57, Kip2)	2.19	2.20

CDKN1C	213348_at	cyclin-dependent kinase inhibitor 1C (p57, Kip2)	2.41	1.88
CDKN1C	213182_x_at	cyclin-dependent kinase inhibitor 1C (p57, Kip2)	1.74	1.81
CDKN1C	213183_s_at	Cyclin-dependent kinase inhibitor 1C (p57, Kip2)	1.50	1.17
CES1	209616_s_at	carboxylesterase 1 (monocyte/macrophage serine esterase 1)	1.83	2.06
CFD	205382_s_at	complement factor D (adipsin)	1.43	1.44
CHMP5	219356_s_at	chromatin modifying protein 5	2.32	2.78
CHMP5	218085_at	chromatin modifying protein 5	2.39	2.56
CHST12	218927_s_at	carbohydrate (chondroitin 4) sulfotransferase 12	1.42	1.58
CIITA	205101_at	class II, major histocompatibility complex, transactivator	-1.45	-1.74
CISH	221223_x_at	cytokine inducible SH2-containing protein	1.36	1.24
CLIC5	213317_at	chloride intracellular channel 5	-1.24	-1.79
CLU	208792_s_at	clusterin	1.10	1.62
CMKLR1	207652_s_at	chemokine-like receptor 1	1.37	2.03
CMKLR1	210659_at	chemokine-like receptor 1	1.27	1.69
CRIM1	202551_s_at	cysteine rich transmembrane BMP regulator 1 (chordin-like)	-1.06	-1.60
CRTAP	201380_at	cartilage associated protein	-1.12	-1.52
CST3	201360_at	cystatin C (amyloid angiopathy and cerebral hemorrhage)	1.55	1.16
CTSL1	202087_s_at	cathepsin L1	2.45	2.40
CUTL1	202367_at	cut-like 1, CCAAT displacement protein (Drosophila)	1.52	1.53
CX3CR1	205898_at	chemokine (C-X3-C motif) receptor 1	1.41	1.40
CXCL1	204470_at	chemokine (C-X-C motif) ligand 1 (melanoma growth stimulating activity, alpha)	-1.71	-1.26
CXCL10	204533_at	chemokine (C-X-C motif) ligand 10	5.79	3.95
CXCR4	211919_s_at	chemokine (C-X-C motif) receptor 4	-1.40	-1.39
CXCR4	209201_x_at	chemokine (C-X-C motif) receptor 4	-1.39	-1.43
DDB2	203409_at	damage-specific DNA binding protein 2, 48kDa	1.23	1.82
DDIT4	202887_s_at	DNA-damage-inducible transcript 4	-1.46	-1.19
DDX58	218943_s_at	DEAD (Asp-Glu-Ala-Asp) box polypeptide 58	2.63	2.83
DHX58	219364_at	DEXH (Asp-Glu-X-His) box polypeptide 58	2.60	2.97
DRAP1	203258_at	DR1-associated protein 1 (negative cofactor 2 alpha)	1.53	1.45
DUSP6	208893_s_at	dual specificity phosphatase 6	1.89	1.53
DUSP6	208892_s_at	dual specificity phosphatase 6	1.62	1.29
ECGF1	217497_at	endothelial cell growth factor 1 (platelet-derived)	1.81	2.03
ECGF1	204858_s_at	endothelial cell growth factor 1 (platelet-derived)	1.66	1.71
EEF1B2 /// hCG_19809 /// hCG_1983058	200705_s_at	eukaryotic translation elongation factor 1 beta 2 /// hCG1983058 /// eukaryotic translation elongation factor 1 beta 2-like	-1.53	-1.67
EEF1D	203113_s_at	eukaryotic translation elongation factor 1 delta (guanine nucleotide exchange protein)	-1.35	-1.50
EEF2	204102_s_at	eukaryotic translation elongation factor 2	-1.33	-1.58
EEF2	200094_s_at	eukaryotic translation elongation factor 2	-1.41	-1.88
EIF2AK2	204211_x_at	eukaryotic translation initiation factor 2-alpha kinase 2	2.09	2.59
EIF3EIP	217719_at	eukaryotic translation initiation factor 3, subunit E interacting protein	-1.70	-2.02
EIF4B	219599_at	eukaryotic translation initiation factor 4B	-1.30	-1.58
EIF4B	211938_at	eukaryotic translation initiation factor 4B	-1.64	-1.88
EIF4B	211937_at	eukaryotic translation initiation factor 4B	-1.64	-1.97
ENC1	201341_at	ectodermal-neural cortex (with BTB-like domain)	-1.43	-1.10
EPB41L3	206710_s_at	erythrocyte membrane protein band 4.1-like 3	1.60	1.63
EPHB2	209589_s_at	EPH receptor B2	2.24	3.25
ETV7	221680_s_at	ets variant gene 7 (TEL2 oncogene)	1.62	1.55
FAM46A	221766_s_at	family with sequence similarity 46, member A	1.52	1.60
FAM46C	220306_at	family with sequence similarity 46, member C	-1.43	-1.32
FAM70A	219895_at	family with sequence similarity 70, member A	3.29	3.57
FANCL	218397_at	Fanconi anemia, complementation group L	1.33	1.63
FBL	211623_s_at	fibrillarin	-1.62	-1.74
FCGR1A	216950_s_at	Fc fragment of IgG, high affinity Ia, receptor (CD64)	1.83	1.54

FCGR1B	214511_x_at	Fc fragment of IgG, high affinity Ib, receptor (CD64)	1.85	1.69
FCN1	205237_at	ficolin (collagen/fibrinogen domain containing) 1	1.55	1.19
FER1L3	201798_s_at	fer-1-like 3, myoferlin (C. elegans)	1.64	1.43
FER1L3	211864_s_at	fer-1-like 3, myoferlin (C. elegans)	1.86	1.34
FFAR2	221345_at	free fatty acid receptor 2	1.94	2.68
FGL2	204834_at	fibrinogen-like 2	1.57	1.36
FLJ11286	218429_s_at	hypothetical protein FLJ11286	1.46	1.78
FLJ11286	53720_at	hypothetical protein FLJ11286	1.61	1.71
FLJ20035	218986_s_at	hypothetical protein FLJ20035	3.34	3.75
FOXO1	202723_s_at	forkhead box O1	-1.58	-1.25
FPRL1	210773_s_at	formyl peptide receptor-like 1	1.51	1.57
FXYD6	217897_at	FXYD domain containing ion transport regulator 6	1.59	1.36
GAS7	202192_s_at	growth arrest-specific 7	-1.38	-1.55
GATM	203178_at	glycine amidinotransferase (L-arginine:glycine amidinotransferase)	-1.55	-1.61
GBP1	202270_at	guanylate binding protein 1, interferon-inducible, 67kDa	2.30	2.32
GBP1	202269_x_at	guanylate binding protein 1, interferon-inducible, 67kDa	1.81	1.80
GIMAP4	219243_at	GTPase, IMAP family member 4	1.51	1.53
GLS	203159_at	glutaminase	-1.56	-1.51
GLUL	200648_s_at	glutamate-ammonia ligase (glutamine synthetase)	1.02	1.56
GMPR	204187_at	guanosine monophosphate reductase	1.38	1.86
GRN	216041_x_at	granulin	1.68	1.50
GRN	200678_x_at	granulin	1.61	1.44
GRN	211284_s_at	granulin	1.70	1.41
GZMB	210164_at	granzyme B (granzyme 2, cytotoxic T-lymphocyte-associated serine esterase 1)	1.34	1.61
H1F0	208886_at	H1 histone family, member 0	2.08	3.13
H2BFS	208579_x_at	H2B histone family, member S	1.05	1.94
HADHA	208631_s_at	hydroxyacyl-Coenzyme A dehydrogenase/3-ketoacyl-Coenzyme A thiolase/enoyl-Coenzyme A hydratase (trifunctional protein), alpha subunit	-1.15	-1.51
hCG_1776259	206548_at	hypothetical protein FLJ23556	1.54	1.91
HERC5	219863_at	hect domain and RLD 5	4.42	4.54
HERC6	219352_at	hect domain and RLD 6	4.24	5.88
HESX1	211267_at	HESX homeobox 1	14.74	11.52
HIST1H1C	209398_at	histone cluster 1, H1c	1.26	2.56
HIST1H2AE	214469_at	histone cluster 1, H2ae	1.13	2.40
HIST1H2AJ	208583_x_at	histone cluster 1, H2aj	1.10	1.77
HIST1H2BC /// HIST1H2BG	214455_at	histone cluster 1, H2bg /// histone cluster 1, H2bc	1.17	2.52
HIST1H2BD	222067_x_at	histone cluster 1, H2bd	1.27	3.37
HIST1H2BD	209911_x_at	histone cluster 1, H2bd	1.39	2.14
HIST1H2BE	208527_x_at	histone cluster 1, H2be	1.11	1.90
HIST1H2BF	208490_x_at	histone cluster 1, H2bf	1.14	2.40
HIST1H2BG	210387_at	histone cluster 1, H2bg	-1.00	4.25
HIST1H2BG	215779_s_at	histone cluster 1, H2bg	1.24	3.13
HIST1H2BH	208546_x_at	histone cluster 1, H2bh	1.10	2.10
HIST1H2BI	208523_x_at	histone cluster 1, H2bi	1.29	2.35
HIST1H3D	214472_at	histone cluster 1, H3d	1.26	3.11
HIST2H2AA3 /// HIST2H2AA4	214290_s_at	histone cluster 2, H2aa3 /// histone cluster 2, H2aa4	1.52	3.14
HIST2H2AA3 /// HIST2H2AA4	218280_x_at	histone cluster 2, H2aa3 /// histone cluster 2, H2aa4	1.33	2.89
HIST2H2BE	202708_s_at	histone cluster 2, H2be	-1.02	2.62
HNRNPA1 /// LOC728844	222040_at	heterogeneous nuclear ribonucleoprotein A1 /// hypothetical protein LOC728844	-1.60	-1.07
HPSE	219403_s_at	heparanase	1.70	1.70
HSPA1A /// HSPA1B	200800_s_at	heat shock 70kDa protein 1A /// heat shock 70kDa protein 1B	1.55	3.00

IFI16	206332_s_at	interferon, gamma-inducible protein 16	1.65	1.78
IFI16	208966_x_at	interferon, gamma-inducible protein 16	1.78	1.76
IFI16	208965_s_at	interferon, gamma-inducible protein 16	1.92	1.74
IFI27	202411_at	interferon, alpha-inducible protein 27	46.60	73.30
IFI30	201422_at	interferon, gamma-inducible protein 30	1.51	1.21
IFI35	209417_s_at	interferon-induced protein 35	2.91	3.19
IFI44	214059_at	Interferon-induced protein 44	4.82	7.09
IFI44	214453_s_at	interferon-induced protein 44	4.50	5.97
IFI44L	204439_at	interferon-induced protein 44-like	6.47	10.07
IFI6	204415_at	interferon, alpha-inducible protein 6	2.80	3.00
IFIH1	219209_at	interferon induced with helicase C domain 1	3.08	3.31
IFIH1	216020_at	Interferon induced with helicase C domain 1	3.20	2.11
IFIT1	203153_at	interferon-induced protein with tetratricopeptide repeats 1	11.25	12.23
IFIT2	217502_at	interferon-induced protein with tetratricopeptide repeats 2	4.67	4.16
IFIT3	204747_at	interferon-induced protein with tetratricopeptide repeats 3	7.59	7.11
IFIT5	203596_s_at	interferon-induced protein with tetratricopeptide repeats 5	2.69	2.74
IFIT5	203595_s_at	interferon-induced protein with tetratricopeptide repeats 5	2.44	2.25
IFITM1	201601_x_at	interferon induced transmembrane protein 1 (9-27)	1.83	2.01
IFITM1	214022_s_at	interferon induced transmembrane protein 1 (9-27)	1.62	1.71
IFITM2	201315_x_at	interferon induced transmembrane protein 2 (1-8D)	1.52	1.70
IFITM3	212203_x_at	interferon induced transmembrane protein 3 (1-8U)	2.11	2.15
IFT81	219372_at	intraflagellar transport 81 homolog (Chlamydomonas)	-1.13	-1.51
IGF2BP3	203820_s_at	insulin-like growth factor 2 mRNA binding protein 3	1.23	1.67
IGFBP7	201162_at	insulin-like growth factor binding protein 7	1.71	1.40
IGHA1	215118_s_at	Immunoglobulin heavy constant alpha 1	-2.13	-1.84
IGHD	213674_x_at	immunoglobulin heavy constant delta	-1.66	-1.44
IGHG1 /// IGHG2 /// IGHG3 /// IGHM ///		immunoglobulin heavy constant gamma 1 (G1m marker) /// gamma 2 (G2m marker) /// gamma 3 (G3m marker) /// mu ///		
IGHV4-31	211430_s_at	immunoglobulin heavy variable 4-31	-1.77	-1.39
IGSF6	206420_at	immunoglobulin superfamily, member 6	1.54	1.38
IL13RA1	201887_at	interleukin 13 receptor, alpha 1	-1.25	-1.53
IL1R2	205403_at	interleukin 1 receptor, type II	-2.05	-1.36
IL1RN	212657_s_at	interleukin 1 receptor antagonist	2.20	2.12
IL1RN	216243_s_at	interleukin 1 receptor antagonist	1.63	1.56
IL1RN	212659_s_at	interleukin 1 receptor antagonist	1.48	1.51
IRF2	203275_at	interferon regulatory factor 2	1.49	1.24
IRF7	208436_s_at	interferon regulatory factor 7	3.24	3.65
IRS2	209184_s_at	insulin receptor substrate 2	-1.45	-1.54
IRS2	209185_s_at	insulin receptor substrate 2	-1.67	-1.69
ISG15	205483_s_at	ISG15 ubiquitin-like modifier	6.16	6.76
ISG20	204698_at	interferon stimulated exonuclease gene 20kDa	1.92	2.54
ISG20	33304_at	interferon stimulated exonuclease gene 20kDa	1.65	1.79
ITGAE	205055_at	integrin, alpha E (antigen CD103, human mucosal lymphocyte antigen 1; alpha polypeptide)	-1.42	-1.57
ITM2C	221004_s_at	integral membrane protein 2C	-1.51	-1.34
JAK2	205841_at	Janus kinase 2 (a protein tyrosine kinase)	1.55	1.65
JAK2	205842_s_at	Janus kinase 2 (a protein tyrosine kinase)	1.68	1.64
JUP	201015_s_at	junction plakoglobin	2.04	3.39
KCTD14	58916_at	potassium channel tetramerisation domain containing 14	1.49	1.82
KIAA0082	212380_at	KIAA0082	1.48	1.35
KIAA0125	206478_at	KIAA0125	-1.64	-1.57
KIAA1466	222139_at	KIAA1466 gene	1.06	1.64
KLRD1	207795_s_at	killer cell lectin-like receptor subfamily D, member 1	1.27	1.51
KMO	211138_s_at	kynurenine 3-monooxygenase (kynurenine 3-hydroxylase)	1.66	1.77

KMO	205306_x_at	kynurenine 3-monooxygenase (kynurenine 3-hydroxylase)	1.57	1.73
KRT10	207023_x_at	keratin 10 (epidermolytic hyperkeratosis; keratosis palmaris et plantaris)	-1.34	-1.54
KRT10	210633_x_at	keratin 10 (epidermolytic hyperkeratosis; keratosis palmaris et plantaris)	-1.46	-1.63
LAG3	206486_at	lymphocyte-activation gene 3	1.33	1.75
LAMP3	205569_at	lysosomal-associated membrane protein 3	4.10	6.02
LAP3	217933_s_at	leucine aminopeptidase 3	2.16	2.04
LBA1	213261_at	lupus brain antigen 1	1.45	1.52
LDLR	202068_s_at	low density lipoprotein receptor (familial hypercholesterolemia)	1.48	1.70
LGALS3BP	200923_at	lectin, galactoside-binding, soluble, 3 binding protein	2.78	3.99
LGALS9	203236_s_at	lectin, galactoside-binding, soluble, 9 (galectin 9)	1.99	1.88
LILRA3	206881_s_at	leukocyte immunoglobulin-like receptor, subfamily A (without TM domain), member 3	1.96	1.57
LILRB1	207104_x_at	leukocyte immunoglobulin-like receptor, subfamily B (with TM and ITIM domains), member 1	1.69	1.36
LILRB1	211336_x_at	leukocyte immunoglobulin-like receptor, subfamily B (with TM and ITIM domains), member 1	1.52	1.29
LILRB2	210146_x_at	leukocyte immunoglobulin-like receptor, subfamily B (with TM and ITIM domains), member 2	1.75	1.54
LILRB2	207697_x_at	leukocyte immunoglobulin-like receptor, subfamily B (with TM and ITIM domains), member 2	1.49	1.49
LILRB3	210225_x_at	leukocyte immunoglobulin-like receptor, subfamily B (with TM and ITIM domains), member 3	1.48	1.28
LILRB3	211135_x_at	leukocyte immunoglobulin-like receptor, subfamily B (with TM and ITIM domains), member 3	1.61	1.22
LILRB3	211133_x_at	leukocyte immunoglobulin-like receptor, subfamily B (with TM and ITIM domains), member 3	1.48	1.16
LMO2	204249_s_at	LIM domain only 2 (rhombotin-like 1)	1.56	1.36
LOC161527 /// PML	211012_s_at	promyelocytic leukemia /// hypothetical protein LOC161527	1.91	2.34
LOC26010	222154_s_at	viral DNA polymerase-transactivated protein 6	3.96	4.32
LOC391020	216565_x_at	interferon induced transmembrane protein pseudogene	2.17	2.68
LOC442171	217379_at	similar to ribosomal protein L10	-1.50	-1.65
LOC442215	217142_at	similar to Elongation FacTor family member (eft-4)	-1.49	-1.63
LOC642741	215963_x_at	similar to ribosomal protein L3 isoform a	-1.50	-1.36
LTA4H	208771_s_at	leukotriene A4 hydrolase	-1.36	-1.73
LY6E	202145_at	lymphocyte antigen 6 complex, locus E	3.40	4.11
LY96	206584_at	lymphocyte antigen 96	1.71	1.49
MAFB	218559_s_at	v-maf musculoaponeurotic fibrosarcoma oncogene homolog B (avian)	1.98	1.65
MARCH1	219574_at	membrane-associated ring finger (C3HC4) 1	1.58	1.55
MARCKS	201670_s_at	myristoylated alanine-rich protein kinase C substrate	2.57	2.43
MARCKS	201669_s_at	myristoylated alanine-rich protein kinase C substrate	2.29	2.07
MARCKS	201668_x_at	myristoylated alanine-rich protein kinase C substrate	-1.04	1.93
MARCKS	213002_at	myristoylated alanine-rich protein kinase C substrate	1.48	1.74
MARCO	205819_at	macrophage receptor with collagenous structure	3.20	2.58
MICAL2	212473_s_at	microtubule associated monoxygenase, calponin and LIM domain containing 2	-1.25	-1.63
MICAL2	212472_at	microtubule associated monoxygenase, calponin and LIM domain containing 2	-1.21	-1.68
MRPL42	217919_s_at	mitochondrial ribosomal protein L42	1.54	1.53
MS4A4A	219607_s_at	membrane-spanning 4-domains, subfamily A, member 4	4.27	3.86
MT1A /// MT1M /// MT1P2	216336_x_at	metallothionein 1A /// metallothionein 1M /// metallothionein 1 pseudogene 2	1.58	1.72
MT1E	212859_x_at	metallothionein 1E	1.51	1.74
MT1F	217165_x_at	metallothionein 1F	1.44	1.80
MT1G	204745_x_at	metallothionein 1G	1.42	1.63

MT1H /// MT1P2	206461_x_at	metallothionein 1H /// metallothionein 1 pseudogene 2	2.27	2.28
MT1P2	211456_x_at	metallothionein 1 pseudogene 2	2.42	2.38
MT1X	208581_x_at	metallothionein 1X	2.36	2.49
MT1X	204326_x_at	metallothionein 1X	1.43	1.56
MT2A	212185_x_at	metallothionein 2A	2.05	2.03
MX1	202086_at	myxovirus (influenza virus) resistance 1, interferon-inducible protein p78 (mouse)	4.33	5.02
MX2	204994_at	myxovirus (influenza virus) resistance 2 (mouse)	3.02	3.13
N4BP1	204601_at	Nedd4 binding protein 1	1.69	1.92
N4BP1	32069_at	Nedd4 binding protein 1	1.58	1.83
N4BP1	221867_at	Nedd4 binding protein 1	1.53	1.54
NAGK	218231_at	N-acetylglucosamine kinase	1.74	1.46
NDC80	204162_at	NDC80 homolog, kinetochore complex component (S. cerevisiae)	-1.01	1.51
NELL2	203413_at	NEL-like 2 (chicken)	-1.52	-1.51
NGFRAP1	217963_s_at	nerve growth factor receptor (TNFRSF16) associated protein 1	-1.07	1.67
NMI	203964_at	N-myc (and STAT) interactor	1.77	1.86
NR4A2	204621_s_at	nuclear receptor subfamily 4, group A, member 2	-2.04	-1.61
NRG1	206343_s_at	neuregulin 1	-1.37	-2.73
OAS1	205552_s_at	2',5'-oligoadenylate synthetase 1, 40/46kDa	4.98	4.29
OAS1	202869_at	2',5'-oligoadenylate synthetase 1, 40/46kDa	4.08	3.91
OAS2	204972_at	2'-5'-oligoadenylate synthetase 2, 69/71kDa	3.89	3.50
OAS2	206553_at	2'-5'-oligoadenylate synthetase 2, 69/71kDa	1.99	1.93
OAS3	218400_at	2'-5'-oligoadenylate synthetase 3, 100kDa	4.12	4.48
OASL	205660_at	2'-5'-oligoadenylate synthetase-like	4.88	4.56
OASL	210797_s_at	2'-5'-oligoadenylate synthetase-like	2.90	4.02
OLFM1	213131_at	olfactomedin 1	-1.62	-2.34
OSBPL10	219073_s_at	oxysterol binding protein-like 10	-1.88	-1.47
P2RX5	210448_s_at	purinergic receptor P2X, ligand-gated ion channel, 5	-1.63	-1.33
P2RY5	218589_at	purinergic receptor P2Y, G-protein coupled, 5	1.46	1.24
PABPC4	201064_s_at	poly(A) binding protein, cytoplasmic 4 (inducible form)	-1.40	-1.53
PAPSS2	203060_s_at	3'-phosphoadenosine 5'-phosphosulfate synthase 2	-1.40	-1.73
PARP11	220315_at	poly (ADP-ribose) polymerase family, member 11	1.14	1.60
PARP12	218543_s_at	poly (ADP-ribose) polymerase family, member 12	2.06	2.44
PCBP2	213264_at	poly(rC) binding protein 2	-1.53	-1.16
PDE4B	215671_at	phosphodiesterase 4B, cAMP-specific (phosphodiesterase E4 dunce homolog, Drosophila)	-1.42	1.01
PDE4B	203708_at	phosphodiesterase 4B, cAMP-specific (phosphodiesterase E4 dunce homolog, Drosophila)	-1.39	-1.13
PF4	206390_x_at	platelet factor 4 (chemokine (C-X-C motif) ligand 4)	1.12	1.70
PGAP1	213469_at	GPI deacylase	2.05	2.00
PGAP1	220576_at	GPI deacylase	1.92	1.91
PHF11	221816_s_at	PHD finger protein 11	1.44	1.80
PID1	219093_at	phosphotyrosine interaction domain containing 1	-1.35	-1.96
PLAC8	219014_at	placenta-specific 8	1.57	1.85
PLAGL2	202925_s_at	pleiomorphic adenoma gene-like 2	1.56	1.33
PLSCR1	202446_s_at	phospholipid scramblase 1	3.03	3.06
PLSCR1	202430_s_at	phospholipid scramblase 1	3.09	2.60
POU2AF1	205267_at	POU class 2 associating factor 1	-1.70	-1.51
PTPRO	208121_s_at	protein tyrosine phosphatase, receptor type, O	1.76	1.72
QARS	217846_at	glutaminyl-tRNA synthetase	-1.34	-1.56
RABGAP1L	213982_s_at	RAB GTPase activating protein 1-like	1.13	1.61
RGL1	209568_s_at	ral guanine nucleotide dissociation stimulator-like 1	1.58	1.54
RGS1	216834_at	regulator of G-protein signaling 1	-1.77	-1.43
RHOB	212099_at	ras homolog gene family, member B	1.37	1.65
RHOBTB1	212651_at	Rho-related BTB domain containing 1	1.13	2.50
RIN2	209684_at	Ras and Rab interactor 2	2.24	1.53
RNASE2	206111_at	ribonuclease, RNase A family, 2 (liver, eosinophil-derived neurotoxin)	2.38	2.86
RPL10L	217559_at	ribosomal protein L10-like	-1.52	-1.76

RPL22	214042_s_at	ribosomal protein L22	-1.38	-1.52
RPL3	211666_x_at	ribosomal protein L3	-1.37	-1.51
RPL4	200089_s_at	ribosomal protein L4	-1.45	-1.56
RPL4	211710_x_at	ribosomal protein L4	-1.53	-1.58
RPL4	201154_x_at	ribosomal protein L4	-1.56	-1.63
RPS5 /// LOC392424	200024_at	ribosomal protein S5 /// similar to ribosomal protein S5	-1.45	-1.56
RRAS	212647_at	related RAS viral (r-ras) oncogene homolog	1.59	1.92
RSAD2	213797_at	radical S-adenosyl methionine domain containing 2	11.73	12.78
RTN1	210222_s_at	reticulon 1	-1.26	-2.05
RTN1	203485_at	reticulon 1	-1.32	-2.17
RTP4	219684_at	receptor (chemosensory) transporter protein 4	3.33	3.13
S100A11	200660_at	S100 calcium binding protein A11	1.78	1.34
SAMD4A	212845_at	sterile alpha motif domain containing 4A	2.78	2.77
SAMD4A	215495_s_at	sterile alpha motif domain containing 4A	3.51	2.56
SAMD9	219691_at	sterile alpha motif domain containing 9	3.74	3.82
SCARB2	201646_at	scavenger receptor class B, member 2	1.62	1.13
SCO2	205241_at	SCO cytochrome oxidase deficient homolog 2 (yeast)	2.34	2.28
SEMA3C	203789_s_at	sema domain, immunoglobulin domain (Ig), short basic domain, secreted, (semaphorin) 3C	-1.46	-1.66
SENP3	215114_at	SUMO1/sentrin/SMT3 specific peptidase 3	-1.39	-1.05
SERPING1	200986_at	serpin peptidase inhibitor, clade G (C1 inhibitor), member 1, (angioedema, hereditary)	4.04	4.93
SIGLEC1	219519_s_at	sialic acid binding Ig-like lectin 1, sialoadhesin	15.65	27.14
SIGLEC1	44673_at	sialic acid binding Ig-like lectin 1, sialoadhesin	5.48	6.41
SLC25A6	212085_at	solute carrier family 25 (mitochondrial carrier; adenine nucleotide translocator), member 6	-1.32	-1.57
SLC27A3	222217_s_at	solute carrier family 27 (fatty acid transporter), member 3	1.50	1.24
SLC31A2	204204_at	solute carrier family 31 (copper transporters), member 2	2.20	1.74
SMCHD1	212569_at	structural maintenance of chromosomes flexible hinge domain containing 1	1.39	1.66
SMCHD1	212577_at	structural maintenance of chromosomes flexible hinge domain containing 1	1.41	1.58
SMCHD1	212579_at	structural maintenance of chromosomes flexible hinge domain containing 1	1.34	1.53
SMOX	210357_s_at	spermine oxidase	1.18	1.84
SP100	202864_s_at	SP100 nuclear antigen	1.64	1.93
SP100	210218_s_at	SP100 nuclear antigen	1.73	1.85
SP100	202863_at	SP100 nuclear antigen	1.53	1.77
SP100	210985_s_at	SP100 nuclear antigen	1.42	1.66
SP110	208012_x_at	SP110 nuclear body protein	1.75	1.94
SP110	209762_x_at	SP110 nuclear body protein	1.80	1.87
SP110	208392_x_at	SP110 nuclear body protein	1.64	1.72
SP110	209761_s_at	SP110 nuclear body protein	1.60	1.70
SPN	206057_x_at	sialophorin (leukosialin, CD43)	1.45	1.10
SPTLC2	216202_s_at	serine palmitoyltransferase, long chain base subunit 2	1.68	1.92
SPTLC2	203127_s_at	serine palmitoyltransferase, long chain base subunit 2	1.50	1.56
STAT1	AFFX-HUMISGF3	signal transducer and activator of transcription 1, 91kDa	2.38	2.57
STAT1	AFFX-HUMISGF3	signal transducer and activator of transcription 1, 91kDa	2.70	2.56
STAT1	AFFX-HUMISGF3	signal transducer and activator of transcription 1, 91kDa	2.65	2.46
STAT1	209969_s_at	signal transducer and activator of transcription 1, 91kDa	2.22	2.37
STAT1	AFFX-HUMISGF3	signal transducer and activator of transcription 1, 91kDa	1.71	1.78
STAT1	200887_s_at	signal transducer and activator of transcription 1, 91kDa	1.56	1.69

TAP1	202307_s_at	transporter 1, ATP-binding cassette, sub-family B (MDR/TAP)	1.83	2.09
TBC1D9	212956_at	TBC1 domain family, member 9 (with GRAM domain)	-1.67	-1.51
TCF3	209153_s_at	transcription factor 3 (E2A immunoglobulin enhancer binding factors E12/E47)	-1.54	-1.29
TCF7L2	216511_s_at	transcription factor 7-like 2 (T-cell specific, HMG-box)	1.42	1.43
TCF7L2	212761_at	transcription factor 7-like 2 (T-cell specific, HMG-box)	1.49	1.38
TCF7L2	212762_s_at	transcription factor 7-like 2 (T-cell specific, HMG-box)	1.56	1.30
TCF7L2	216037_x_at	transcription factor 7-like 2 (T-cell specific, HMG-box)	1.44	1.30
TCF7L2	216035_x_at	transcription factor 7-like 2 (T-cell specific, HMG-box)	1.46	1.25
TCN2	204043_at	transcobalamin II; macrocytic anemia	1.57	2.16
TDRD7	213361_at	tudor domain containing 7	1.77	1.64
TFEC	206715_at	transcription factor EC	1.60	1.48
THBS1	201110_s_at	thrombospondin 1	-1.54	-1.61
TLR1	210176_at	toll-like receptor 1	1.61	1.59
TLR7	220146_at	toll-like receptor 7	2.65	2.24
TMEM140	218999_at	transmembrane protein 140	1.73	2.41
TMOD3	220800_s_at	tropomodulin 3 (ubiquitous)	1.29	1.63
TNFAIP3	202643_s_at	tumor necrosis factor, alpha-induced protein 3	-1.74	-1.09
TNFAIP3	202644_s_at	tumor necrosis factor, alpha-induced protein 3	-1.40	-1.16
TNFAIP6	206026_s_at	tumor necrosis factor, alpha-induced protein 6	1.81	2.07
TNFSF10	202687_s_at	tumor necrosis factor (ligand) superfamily, member 10	3.10	2.34
TNFSF10	202688_at	tumor necrosis factor (ligand) superfamily, member 10	2.36	2.04
TNFSF10	214329_x_at	tumor necrosis factor (ligand) superfamily, member 10	2.96	2.01
TOR1B	209593_s_at	torsin family 1, member B (torsin B)	1.98	1.89
TPD52	201690_s_at	tumor protein D52	-1.46	-1.09
TPMT	203672_x_at	thiopurine S-methyltransferase	1.63	1.22
TRAFD1	202837_at	TRAF-type zinc finger domain containing 1	1.91	1.99
TREX1	205875_s_at	three prime repair exonuclease 1	2.17	1.97
TREX1	34689_at	three prime repair exonuclease 1	1.70	1.39
TRIM14	203148_s_at	tripartite motif-containing 14	1.57	1.51
TRIM21	204804_at	tripartite motif-containing 21	1.54	1.64
TRIM22	213293_s_at	tripartite motif-containing 22	1.61	1.72
TRIM34 /// TRIM6-TRIM34	221044_s_at	tripartite motif-containing 34 /// tripartite motif-containing 6 and tripartite motif-containing 34	1.69	1.69
TRIM38	203610_s_at	tripartite motif-containing 38	1.64	1.82
TRIM38	203567_s_at	tripartite motif-containing 38	1.59	1.63
TRIM5	210705_s_at	tripartite motif-containing 5	2.41	1.81
TRIP6	209129_at	thyroid hormone receptor interactor 6	1.19	1.57
UBE2L6	201649_at	ubiquitin-conjugating enzyme E2L 6	1.92	1.91
UNC119B	202365_at	unc-119 homolog B (C. elegans)	-1.31	-1.58
USP18 /// LOC727996 /// LOC728216 /// LOC728438	219211_at	ubiquitin specific peptidase 18 /// similar to ubiquitin specific peptidase 18	5.64	7.43
UTRN	213023_at	utrophin	1.44	1.51
VDR	204255_s_at	vitamin D (1,25- dihydroxyvitamin D3) receptor	1.23	1.60
WSB1	210561_s_at	WD repeat and SOCS box-containing 1	1.35	1.52
XAF1	206133_at	XIAP associated factor-1	3.15	4.25
ZBP1	208087_s_at	Z-DNA binding protein 1	2.29	3.03
ZCCHC2	219062_s_at	zinc finger, CCHC domain containing 2	1.94	2.26
ZNF322B	219376_at	zinc finger protein 322B	1.36	1.46
---	213294_at	Full-length cDNA clone CS0DK002YF13 of HeLa cells Cot 25-normalized of Homo sapiens (human)	2.83	3.34
---	215617_at	CDNA FLJ11754 fis, clone HEMBA1005588	2.01	3.12
---	216297_at	MRNA; cDNA DKFZp564C156 (from clone DKFZp564C156)	-1.40	-1.11
---	212605_s_at	AF034176 Human mRNA (Tripodis and Ragoussis) Homo sapiens cDNA clone ntcon5 contig	-1.15	-1.60

Note: all genes were significantly affected by the interferon at day 3 ($p<0.001$)
